# Gender Inequality Prevents Abused Women from Seeking Care Despite Protection Given in Gender-Based Violence Legislation: A Qualitative Study from Rwanda

**DOI:** 10.1371/journal.pone.0154540

**Published:** 2016-05-06

**Authors:** Aline Umubyeyi, Margareta Persson, Ingrid Mogren, Gunilla Krantz

**Affiliations:** 1 Department of Epidemiology and Biostatistics, School of Public Health, College of Medicine and Health Sciences, University of Rwanda, Kigali, Rwanda; 2 Department of Nursing, Umeå University, Umeå, Sweden; 3 Department of Clinical Sciences, Obstetrics and Gynecology, Umeå University, Umeå, Sweden; 4 Department of Public Health and Community Medicine, Section of Epidemiology and Social Medicine, Sahlgrenska Academy at the University of Gothenburg, Gothenburg, Sweden; Örebro University, SWEDEN

## Abstract

**Objective:**

Despite its burden on a person’s life, Intimate Partner Violence (IPV) is known to be poorly recognised and managed in most countries and communities. This study aimed to explore health care professionals’ experiences of the health care seeking processes of women exposed to intimate partner violence in Rwanda.

**Methods:**

Six focus group discussions were conducted in three district hospitals and three mental health units in Rwanda. A sample of 43 health care professionals with various professions and length of work experience, who regularly took care of patients subjected to IPV, was selected for focus group discussions. The analysis was performed using qualitative content analysis.

**Results:**

The theme *“Gendered norms and values defeat the violence legislation in women’s health care seeking when women are abused”* expressed the health care professionals’ experiences of the double-faced situation which women exposed to IPV met in their help seeking process. Positive initiatives to protect women were identified, but the potential for abused women to seek help and support was reduced because of poverty, gender inequality with prevailing strong norms of male superiority, and the tendency to keep abuse as a private family matter.

**Conclusion:**

Legislative measures have been instituted to protect women from abuse. Still many Rwandan women do not benefit from these efforts. The role of the health care services needs to be reinforced as an important and available resource for help and support for abused women but further legislative changes are also needed. Initiatives to further improve gender equality, and institutionalised collaboration between different sectors in society would contribute to protecting women from IPV.

## Introduction

Intimate Partner Violence (IPV) against women represents a widespread global health problem [1], rooted in unequal power relations between men and women. It is a grave transgression of women’s human rights [2,3]. In most societies, women’s dependency and their submission to or transgression of gender norms contribute to IPV [4]. Although both women and men are exposed to IPV, more women are affected and they face more severe violence [5,6], resulting in mental and physical health problems [5,7,8], serious injuries, death [9], and the spread of sexually transmitted diseases [2,10].

In some cultures, IPV may be regarded as part of life. Hence, the violence is accepted or viewed as a family matter and not revealed to anyone [11–14]. Due to such societal norms, reporting or leaving an abusive relationship becomes less likely as it would result in devastating life circumstances for many women [15,16]. Consequently, many IPV exposed women do not seek help, but choose to suffer in silence [12,17].

In Rwanda, efforts have been made to protect women in abusive relationships. A law against gender-based violence (GBV) was constituted in 2008 [18], followed by a national gender policy [19]. In that law, GBV is defined as any act, perpetrated because of the victim’s gender, which results in bodily, psychological, sexual or economic harm, or in the deprivation of freedom or in negative consequences within or outside households [18]. This definition informs this study except that we do not consider violence outside the household but focus solely on Intimate Partner Violence (IPV). Additionally, in the 2012 Rwandan penal code, marital rape is a crime. Any person committing marital rape is subject to imprisonment and fines [20]. Other protective initiatives include arranging anti-GBV clubs in schools and introducing GBV-committees in communities, aiming to improve people’s knowledge about their rights and to secure the reporting of IPV [21]. Integrated services (i.e. medical care, psycho-social support, access to legal support and aid, and emergency accommodation for a few days) are available in “One-Stop Centres”. These centres were created within the Rwandan Police to provide free services to survivors of child domestic abuse and gender-based violence. In Rwanda, there are currently twelve “One-Stop Centres”. The annual number of visits varies somewhat. In the years 2010–12, numbers of yearly cases were 3427; 3585 and 3444 respectively [21,22].

The 2010 Rwanda Demographic and Health Survey (RDHS) [23] reported that 42% of women who have ever experienced physical or sexual violence seek help from formal and informal sources. However, the majority of women prefer to use informal sources for help, such as family, in-laws, friends or neighbours. This is in line with what is seen in most countries i.e there are various reasons for women not reveal ongoing abuse within the family [12,16,24]. Women exposed to both physical and sexual violence are more likely to seek help than women only exposed to sexual violence [23], which indicates that only the more serious violations are ever sought help for. The DHS further report that only 27% of the exposed women seek help from the police or other unspecified formal facilities, such as the health care services [23].

Moreover, exposed women who want to report or leave a relationship are restrained by socio-cultural factors positioning women in the home, and men as the authority figures who control family finances and take decisions. Women are therefore restrained by economic dependency on their husbands and also by legal and historical factors, such as laws on child custody in case of separation or lack of support related to the loss of family members during the Rwandan genocide [16]. According to the Rwandan civil code, in the event of divorce, the mother can only retain custody of very young children and has to deliver them to their father once weaned. Under this code, child custody is supposed to go either to the innocent spouse i.e the spouse who has been granted the divorce or, in the interests of children, to the spouse who can best ensure their education [25], which is the father in most cases. A Kenyan study [12] presents findings in line with those reported by the RDHS [23], showing that women generally take no action when they are exposed to IPV. If women do act, they initially contact their original family and thereafter make contact with available community structures or hospitals. As a last option, police and legal structures are approached [12]. In this stepwise process of action, women face a number of barriers. These barriers consist of: a) the absence of a link between different authorities and services, b) health services with poor knowledge, skills and commitment, and c) community members who tend to normalize violence [12]. Also, penalties such as jail or fines are inadequate or counterproductive as they often prevent women from acting and reporting due to dependence on their husbands for financial resources [12].

Even though Rwanda has taken proactive steps to protect women from violence and its consequences, most exposed women prefer to seek help from family and friends instead of using available formal services and supportive organisations [12,16]. Despite this poor general utilization of formal services including health services when exposed to IPV, health care professionals receive serious cases of IPV. Additionally, health care professionals are contacted for medical expertise to get the proofs for the legislation purpose; therefore health care professionals have gain a broad knowledge of women’s health seeking process in case of IPV and could be a trustful resource in the future. To get a better understanding of the prevailing situation, the aim in this qualitative study was to explore health care professionals’ experiences of the health care seeking processes of women exposed to intimate partner violence.

This study forms part of a project on violence and other traumatic episodes, mental health and barriers to care among young men and women: The Rwandan Violence, Mental Health and Barriers to Care project (RwVMHBC project).

## Methods

### Study Design

Data was collected by focus group discussions (FGD) [26] among health care professionals, in purposively selected health facilities. The analysis was done using qualitative content analysis [27,28].

### Setting and Participants

District hospitals and mental units (clinic and hospitals) situated within the capital, Kigali city and the Southern Province of Rwanda were included. Three district hospitals were chosen to represent the experiences of health care professionals encountering a variety of health problems in primary care and within the community, including intimate partner violence. The three mental health units (one mental clinic and two mental hospitals) were selected to represent the experiences of health care professionals providing care to complicated cases, where IPV may have contributed to mental disorders.

Thereafter, the Director of each setting was approached by the research team with information on the study and, was asked to facilitate the recruitment of six to eight eligible participants. In this process, the eligible health care professionals, i.e. fulfilling the inclusion criteria were informed about the research project objective. The inclusion criteria for participation were: a) being a health care professional caring for abused patients on a regular basis and b) being willing to share those experiences.

A total of 43 health care professionals (23 females and 20 males) participated in six FGDs with 6 to 10 participants in each FGD. Most participants were specialised mental health nurses, nurses in charge of GBV services, or unspecialised nurses. Medical doctors, clinical psychologists and psychiatrists also participated. The median age of the participants was 32 years (25^th^–75^th^ quartile: 29–38 years). Their work experience ranged from 1 month to 16 years. The detailed characteristics of informants are provided in table 1.

**Table 1:** Characteristics of the focus-group discussion participants: gender, age and profession.

Position	Male/female	Age range (years)	Profession (n)
S1 File	3/3	26–40	Psychiatrist(1)
			Mental health nurses(3)
			Clinical psychologist(2)
S2 File	7/3	29–50	Psychiatrist(1)
			Mental health nurses(7)
			General nurse(1)
			Clinical psychologist(1)
S3 File	1/6	26–38	Mental health nurse(1)
			Clinical psychologist(1)
			General nurses(5)
S4 File	4/3	28–59	General nurses(5)
			Mental health nurse(1)
			General nurse in charge of GBV(1)
S5 File	1/5	27–44	Mental health nurse(1)
			General nurses(4)
			Clinical psychologist(1)
S6 File	4/3	27–46	Mental health nurses(5)
			Clinical psychologists(2)

### Procedures

The interview guide was developed based on available literature and on findings from a population-based survey, performed within this research project [5]. A pilot FGD was conducted to test the comprehensibility of questions. This resulted in minor revisions of the interview guide. The guiding questions were of a comprehensive character, such as: “What happens when a young adult has been exposed to intimate partner violence and what is your professional experience on how they manage or how they handle this problem?”, “From your professional experience, what are the major barriers to seeking professional help or professional health care?” and, “If you were to create an ideal care for young adults exposed to violence, disregarding economical and organisational hindrances, what would this good care of young adults exposed to intimate partner violence look like?”. The questions posed enabled the participants to discuss and reflect on their professional experiences of providing care to people exposed to IPV.

All FGD were performed in an undisturbed venue within each selected health facility. The FGDs were facilitated by a moderator (AU) with the assistance of an experienced note-taker. An observer (MP) took notes of observed interactions that complemented the recorded data. The FGDs lasted between 65 and 135 minutes, were performed in Kinyarwanda and digitally recorded. All recordings were transcribed into Kinyarwanda and later translated into English. The quality of the translation was controlled by back-translation of parts of the material by an independent person, proficient in English, to confirm the accuracy of the linguistic translation.

### Analysis

The translated text was analysed by use of qualitative content analysis (CA). Qualitative CA focuses on the subject and the context, highlighting the differences and similarities within codes and categories, and addresses the manifest and the latent content of the experiences under study [28]. The emerging codes and categories result in an overarching theme that may be understood as a thread of underlying meaning throughout the whole analysis on an interpretative level [28]. Finally, a theme was identified, based on the underlying meaning throughout the codes, sub-categories and categories [27,28].

The analysis was performed in several steps. First, the entire text (all FGDs) was read thoroughly in order to achieve a sense of the whole and to identify content areas. Second, the text was divided into meaning units addressing the aim, i.e. words, sentences or paragraphs related to each other by content or context, were identified. The meaning units were shortened into condensed meaning units and thereafter labelled with codes. This procedure was performed by the authors AU and MP, later with support from GK. Third, all codes were continuously compared for similarities and differences, which resulted in two categories and their six corresponding sub-categories. During this process, the theme expressing the underlying meaning of health care professionals’ experiences emerged. To seek agreement and to validate the findings and context, the final steps of the analysis were discussed in the research group until consensus was achieved.

### Ethical Considerations

The RwVMHBC project was authorized by the National Institute of Statistics of Rwanda (No 1043/2011/10/NISR) and approved by the Rwanda National Ethics Committee (Review Approval Notice No 165/RNEC/2011). Prior to all FGDs, participants were informed of the aims, the procedure of the study and their right to withdraw from the study at any time. All participants signed an informed consent and gave their permission to record the discussions before the FGDs started. No identifying information was entered in the transcribed text to secure anonymity.

## Results

The findings comprise a theme with two categories and six subcategories. The analysis resulted in two main lines of findings in relation to abused women’s help seeking: 1) aspects related to perceived challenges or problems and 2) facilitating factors. The sub-categories and their corresponding categories are presented below (Table 2). Quotations from the FGDs illustrate the sub-categories. Finally, the theme is presented.

**Table 2:** An overview of theme, categories and sub-categories.

Sub-categories	Categories	Theme
Experiencing imbalances between cultural norms and legislation on gender-based violence in a changing society	Challenges faced by abused women seeking health care.	Gendered norms and values defeat legislation on gender-based violence in women’s health care seeking when women are abused
Experiencing women’s financial dependency on men		
Encountering ignorance of sexual violence		
Experiencing women’s low awareness of where to seek care and support		
Experiencing women’s improved awareness of their rights	Understanding how women’s protection is facilitated by community and legal actions.	
Acknowledging women’s improved protection by society		

### Category 1: Challenges Faced by Abused Women Seeking Health Care

#### Experiencing imbalances between cultural norms and legislation on gender-based violence within a changing society

A conflict was perceived between, on one hand, policy and legislation aiming to protect women against partner violence and, on the other hand, gender norms and values embraced by society advocating the superior role of men in the society and in the family. This situation of conflicting standpoints was also observed to affect abused women’s help-seeking behaviour. Gender-based differences in interpretation of policy and legislation aiming to end abuse against women were understood to be a cause of conflict within relationships. Many women saw possibilities for improving their situation based on the new legislation, while many men did not accept the new legislation preferring to hold on to traditional gender values. These men were perceived by health care staff as being reluctant about, resistant to, societal change, and as continuing to abuse their spouses to remain in control.

“*Some men feel that they need to be in control of women*, *so they keep traumatising them day after day*.”

(S5 File)

Some participants noted that wives were expected to be submissive to their husbands. Such expectations caused women to abstain from seeking health care in order to avoid more violence and abuse, and to protect the reputation of their husband and family. One participant said:

“*Whatever violence may happen to her*, *she opts to accept the situation as the fate of a housewife in a family*, *because culturally a housewife is an individual who is supposed to accept all family matters*.”

(S3 File)

The traditional gender norm of men being superior to women was supported by the church and its leaders. Participants stated that the church also consider marriages to be everlasting, which means that a woman has to accept to living with her husband even if he uses violence against her, because divorce is not an alternative.

“*Churches also advocate for an eternal marriage which is the cross women have to bear*.*”*

(S3 File)

### Experiencing Women’s Financial Dependency on Men

Participants encountered women in need of medical assistance due to recurrent violence and abuse perpetrated by the husband. Such patients repeatedly suffered from acute physical injuries and/or long-term psychological problems. Poverty appeared as one major factor which delayed or stopped women from seeking help. Insufficient financial means for household expenditures made medical treatment unaffordable and prevented women from seeking care. Another major obstacle discussed was a women’s dependency on her husband: the head of the family, the breadwinner; and the person in control of family economy and decisions.

*“I’ll give you an example of economic violence; it is when a wife has a husband who does not provide for home needs*. *When he sells their goat*, *the wife is not given a penny to buy*, *for example*, *the child’s school dress*. *Such economic violence influences the wife’s mental health and her dignity is reduced even in her own home*.*”*

(S5 File)

Family matters, including violence and abuse against women, were to be held secret and retained within the family, which delayed health care-seeking. Several cases were described where the actual cause of the injuries or health problems was not revealed until after several consultations, when the woman felt that she trusted the health care professionals. The health care professionals also reported that sometimes they suspected IPV as the reason behind a woman’s health problems, but that many women repeatedly refused to reveal the violence.

*“We often receive cases of wives brought to our emergency service as they have been beaten by their husbands*. *They then try to convince us that they have fallen*. *[…] The wife would not reveal the secret*, *but later when she feels more secure*, *she might tell that she has been beaten by her husband*. *Many women however prefer to keep it as a secret*.*”*

(S3 File)

*“If there has been sexual abuse*, *it will be very difficult to talk about it*. *Because of culture*, *the person will keep it to herself*, *then it will take a long time to get the story out of her”*.

(S2 File)

### Encountering Ignorance on Sexual Violence

Health care professionals discussed not only cases of married women seeking health care due to their husbands’ sexual violence, but also health care sought due to rape of single women or children, mainly girls. Poor awareness of the fact that marital rape was considered a crime led to few married women seeking care. In addition, health care professionals stressed that the possibility of claiming sexual abuse was reduced by the fact that obvious forensic signs of rape were demanded for prosecution. Therefore, women who had been sexually abused often hesitated to seek appropriate care and to press charges.

*“It should be understood that finding sperm in the vagina is not the only sign of rape*.*”*

(S4 File)

### Experiencing Women’s Low Awareness of Where to Seek Care and Support

Health care professionals reported that most women initially turned to their original families when they were abused. In a second step, abused women approached various facilities, such as local authorities, the police force, health centres or hospitals. Organisations working for women’s rights and empowerment were used by some women. The “One-Stop Centres”, created as a resource for abused women, were briefly mentioned in some of the FGDs, but few women seemed to turn to such a unit as this was equivalent to revealing the abuse to the entire community, thus bringing shame to the family. Hence “One-stop centres” did not appear to be the preferred service for many abused women. In one FGD, the help seeking process was described as a long chain of numerous contacts in order to receive the needed support. Therefore, health care professionals suggested that full support services should be made available within the general health care services and preferably within each district hospital.

*“It [the help seeking] is a chain of events*. *It starts with families where they make recourse to local leaders*. *The latter [local leaders] usually send them to community police at cell or sector level*. *The patient is then taken to the health centre*, *from where she is further transferred to the hospital*. *Upon arriving at the hospital*, *the person in charge of GBV checks her case and transfers her either to the mental health service or to the police if the case is to be followed up as a criminal act*.*”*

(S4 File)

As presented above, these challenges forming the category *“Challenges faced by abused women seeking health care”* have its roots in poverty and gender inequality.

### Category 2. Understanding How Women’s Protection Is Facilitated by Community and Legal Actions

#### Acknowledging women’s improved protection by society

Most participants agreed that the government initiatives aiming to empower women and improve their status in society and within families were successful in many respects. Policy making, legislation and police interventions were perceived to contribute to the protection of women. Some health care professionals gave examples of good collaboration between the police and hospitals, for example when securing evidence of violence or rape. However, participants in one FGD discussed weaknesses in the support system for abused women. They expressed the view that many professionals lacked the necessary knowledge and skills because there was not enough instruction or guidance for health care professionals on how best to support and counsel abused women.

*“Patients are followed up both administratively and health wise*. *The police follow up the case regarding the violence and we follow up the case by treating the victim*.*”*

(S4 File)

*“You can do counselling*, *but if you do not guide the abused woman until she has solved her problem*, *you are not of much help*. *So*, *guidance and counselling should both be included in educational curricula for health care staff so that they are more knowledgeable*.*”*

(S4 File)

### Experiencing Women’s Improved Awareness of Their Rights

The participants discussed how support groups and organisations (such as Haguruka, a Non-Governmental Organisation for the defense of women and children’s rights) provided knowledge and support within the community through educational initiatives on IPV. These preventive initiatives made women more aware of their legal rights, and also empowered women to report the abuse they were exposed to. More abused women were perceived as being willing to press legal charges than was previously the case. Women in the community also seemed to support and advise their peers to turn to the police when abused. The professionals stressed that they themselves could play a further role in preventing IPV by providing knowledge and information to all women as they had gained vast experience over the years within their respective professions.

*“Nowadays*, *women are aware of their rights*. *Although they do not express it openly*, *we realise that there has been a change*. *I think that the solution is to continue to increase the awareness […]*. *We [health care professionals] could convince them that if they hide their husbands’ use of violence*, *they later risk being killed by them*.*”*

(S3 File)

The health care professionals reported that some women used threats to call the police as a way of preventing their husbands from using violence. However, the improved community awareness of IPV as a criminal act could strike back and aggravate a woman’s situation. Participants related that some abusive husbands feared legal punishment, and therefore the most severe violent acts would be performed in strict privacy so that neighbours would not be able to overhear and report them.

*“People avoid being as openly involved in violence as they used to be in the past*.*”*

(S3 File)

The theme expressing the overarching experience was titled *“Gendered norms and values defeat legislation on gender-based violence in women’s health care seeking when women are abused”* and summarized the health care professionals’ perceptions about both challenges and facilitating factors in health care seeking in women exposed to partner violence. In the first category, “*Challenges faced by abused women seeking health care”*, all four sub-categories describe various gender inequalities, such as women being financially dependent, ignorant, and with less power over decision-making than men. These factors contributed to abused women’s poor health care seeking because seeking help could worsen a woman’s life circumstances even though she would still have to endure the violence. The second category “*Understanding how women’s protection is facilitated by community and legal actions”* indicated that there was societal awareness of gender inequality and gender-based violence and that policy and legislation to protect women had been instituted, although it became evident that challenges outweighed facilitating factors.

## Discussion

### Summary of Main Findings

We found a prominent disagreement between policy and legislation aiming to protect women, and strong gendered norms or values stressing male superiority and female submission, which affected women’s health care seeking when they were exposed to male partner violence. As women sensed some freedom and improved rights, the violence could even increase because some men felt threatened and tried to maintain the prevailing situation. Despite the appreciated initiatives to protect women, the majority of abused women were perceived not to benefit from available health and support services. Women feared revealing the abuse to anyone within or outside the family as this would bring shame to the family and worsen their overall life situation. Gaps in the involvement of health care professionals as well as in the legislation in supporting abused women were highlighted.

### Comparison with Other Studies

In our study, health care professionals expressed that it appeared difficult to many men and some women to abandon old, gendered traditions in favour of a more gender equal society. In line with our findings, others have shown that some husbands perceive new policies as a threat to their power in the family, while the same policies may prompt some wives to sense some freedom. This disparity may destabilise the family, and lead to more violence [24,29]. A Rwandan study using an ethnographic design points to an ongoing conflict between cultural norms and the implementation of gender equal policies [29]; some of the gender violence prevention programs are influenced by traditional gender norms and beliefs, and as a result continue to maintain gender-based violence [29]. A United Nations report from a Rwandan setting states that poverty, consumption of alcohol and drugs, ignorance and misinterpretation of gender roles are major causes of physical abuse [30].

Further, women’s poverty and financial dependency on the husband were identified as important barriers to care seeking in our study. The association between poverty and IPV has been previously confirmed in Rwanda [16,31,32]. Additionally, IPV and poverty both contribute to increased stress, powerlessness and social isolation among exposed women [33]. Economical violence was illustrated in our study as when women and children are not provided for. It has been shown elsewhere that such violence is often present in conjunction with other types of violence [16]. Due partly to women’s financial dependency on their husbands, abuse within a relationship was described in our study as most often kept secret by women seeking health care. This resistance to reporting family violence is supported by other Rwandan studies [16,29] and other researchers [24]. In addition, some women are found to withdraw their legal complaints after the husband is arrested, which may in part be due to the family’s likely financial suffering if the husband is put in jail. Remaining silent is also a strategy used by some women to protect the reputation of their husband and family, but also to protect their own reputation [16]. Introducing legal complaints against a husband can have a negative impact on a woman’s reputation and on her capacity to care for her children [29].

Societal initiatives supporting women’s protection from violence, such as policy making, legislation and police interventions, were seen by the health care professionals in our study as facilitating factors for abused women’s health care seeking. Earlier studies suggest evidence that initiatives tackling gender inequality and power imbalance can reduce violence against women in low and middle-income countries [34]. However, it has been emphasized that such preventive initiatives demand close collaboration between sectors in order to become effective. Other studies go further and point at the importance of involving all societal levels i.e. individual, interpersonal, community and societal, to reduce gender inequality and power imbalance and thus to reduce violence against women [35].

The health care professionals were familiar with many of the health problems and social problems related to IPV against women. Health care professionals further suggested that they should be more involved in the work with preventing IPV in order to improve the situation of women exposed to IPV. An earlier study also points at the health sector as an important partner in the work of preventing IPV [35], consequently health care professionals’ knowledge of the consequences of IPV on women’s physical and mental health could be used to improve women’s health.

Furthermore, some legislative modifications are needed. Changing the child custody laws as well as establishing special family violence courts with trained judges to prosecute perpetrators and to meet women reporting such violence would improve women’s situation. Additionally, reforms strengthening women’s education and employment need to be reinforced to address the present financial dependency. Also, more knowledge is needed about health care professionals’ abilities in case-finding and how health care professionals can best support women exposed to IPV. Furthermore, more knowledge about how abused women perceive support from health facilities is required. Such studies could be designed as observational studies in facilities caring for women exposed to violence, and as further in-depth interviews with abused women. Likewise, studies on how to improve collaboration between health services, shelters, the judiciary and the police could add valuable strategies for reducing IPV directed at women and for offering efficient integrated services to women and children. Strategies for how to treat men who use violence within the family also need to be investigated and tested.

### Strengths and Limitations of the Study

This study applied a purposive selection of participants working in mental health facilities and district hospitals where abused women could seek help for physical and mental health consequences of IPV. The health care professionals were men and women of different age groups, with differing professional backgrounds working in general care (district hospitals) and in mental health services. Interviewing health care professionals about victims of partner violence may be regarded as secondary data as they are retelling others’ stories. However, it can also be seen as a broad pattern of combined experiences gained from repeated encounters with a number of women exposed to IPV. This was our intention when approaching the health care professionals, selected due to their broad understanding of the factors affecting abused women’s help seeking, gained through in-service trainings and work experience. An additional strength of this study is that the research team consisted of professionals with differing scientific backgrounds, which contributed to a multifaceted discussion through the entire research process. A clear picture was given of how health care seeking among abused women was perceived, and a comprehensive description of the data collection and analysis is presented with citations from the FGDs to strengthen the trustworthiness of our findings. We believe that the perceptions and experiences of the health care professionals in this study represent only views of those who participated in the FGDs, but might be transferable to other health care professionals meeting abused women in similar type of health facilities in Rwanda.

## Conclusions

This study revealed the short-comings of legislation aimed at protecting women from IPV, as the influence of such legislation was found to be dominated by the strong imprints of gendered norms and values. Gender equality should be improved by additional legislative measures and attitudinal change to secure protection against violence and to safeguard women’s health and rights. Not least, the present legislation on gender-based violence needs revision, particularly in relation to rape, where forensic proof is demanded as it constitutes a barrier to women seeking care and taking legal action. The health care professionals have a responsibility for prevention, case-finding and treatment of women exposed to violence. Health care professionals should be better prepared through further education, and guidelines on how to handle such cases. Collaboration between health care professionals, police and the judiciary to facilitate women’s health care seeking, in line with human rights principles and the right to health, should be improved. To make women less dependent on their husband, women’s education and employment opportunities should be improved as this is shown to make marriages more equal and less violent.

## Supporting Information

S1 File Interview with health care staff from Kigali trauma center/ CHUK department. (PDF)

S2 File Interview with health care staff from CARAES Ndera. (PDF)

S3 File Interview with nurses from Kabgayi hospital. (PDF)

S4 File Interview with nurses from Nyanza hospital. (PDF)

S5 File Interview with the medical staff of Remera Rukoma hospital. (PDF)

S6 File Interview with nurses from CARAES Butare. (PDF)
