# Supplementary material for: Gender Inequality Prevents Abused Women from Seeking Care Despite Protection Given in Gender-Based Violence Legislation: A Qualitative Study from Rwanda
Source: PLoS One. 2016 May 6;11(5):e0154540. doi: 10.1371/journal.pone.0154540 (PMC4859471; doi:10.1371/journal.pone.0154540)
Supplement: S1 File — (PDF) [file pone.0154540.s001.pdf]

SUPPORTING FILE 1

INTERVIEW HELD WITH HEALTH CARE STAFF FROM KIGALI TRAUMA CENTER/ CHUK DEPARTMENT

Date: October 22, 2012

Time: 15h40- 16h33

Participants: 5

Q1: Could you tell us first of physical or physiological problems you mostly encounter? What are the most frequent?

3: What do you mean by physical? Most of our problems are more psychosomatic rather than physical.

Prob: Let me maybe talk about a wound which is may be physical, it is just an example.

3: There are often cases of chronic headache that can last for a long time like 2 years or 5. In my opinion, this is what can be considered as physical.

1: There are also some other problems that can be called psychosomatic, apart from the headache, you can find some other related problems such as insomnia for example, or feeling some pain in the whole body like in the back and stomach, and all this can be termed psychosomatic.

Prob: Others, what do say about this?

Patients we receive here can be classified into three categories: Psychosomatic, psychosis and neurological disorders. There are many disorders in psychosis.

Prob: Others, what do you think about this? Is there any other problem in addition to what has been just mentioned above?

1. There is depression; I think it was not mentioned.

4: What I can add to what has been mentioned is that we most often receive some other cases related to anxiety disorders such as PTSD, panic, these are mostly cases we have received.

Q: When you look at this professional life you live in, do you find any difference between men and women with regard to these problems or mental issues?

1: Yes, there is a clear difference. There are some illnesses which affect more female patients than male such as PTSD, likewise in these psychosomatic problems and depression. But with epilepsy, their number is almost equal.

Prob: Others, what do you think? Are there specific illnesses for women or men or simply specific to men?

4: You mean if there are specific sex related illnesses. Looking at the number of patients who come to us, many of them are women. But also with reference to the general population census, there are more women than men. Also, with reference to our culture, the number of women is greater than that of men who come to us.

Prob: Apart from the fact that the number of women is higher than that of men, is there any other reason why women are affected by PTSD? Do you think there are other reasons apart from their numbers?

1: I hear people saying that women are more emotional than men; this can also be a factor.

Prob: What do others think?

4: In my view, some of the reasons that push more women than men to come to us are rooted in our context whereby women are different from men in terms of how they behave when they have problems. For a man, a request for help means acceptance of failure to solve his problem. This means that accepting the failure means trying many possible ways to get rid of the problems. Some still try these many ways by doing other things. This does not mean that they are not ill, but rather they make recourse to other things like drinking beer. They use other mechanisms that help them to survive. As for women, they come to this centre in big numbers. But this does not mean if you go down to the community level you cannot find many men with mental problems.

Prob: So, does it mean that illness is considered as a weakness in the Rwandan cultural context as if one has failed a case?

5: In my opinion, women easily come to hospitals either for mental or physical problems, unlike men, they do not like to go for treatment, and they are always reluctant. They sometimes go for routine examination.

Q: Now, we want to know, if you dig deep into these problems, do you see any link between these problems and related consequences of genocide which you had not mentioned before? Do you think many PTSD are related to genocide?

3: PTSD, pain of the whole body, sometimes people has stomachache, backache, aches in many parts of the body. This is what our colleague said, that a patient may come with psychosomatic problems that appear physical, but which are in reality psychological.

Prob: Do we have many of these cases?

3: These cases are very frequent.

Q: As time goes on, do not you see that there are only cases of PTSD? Or else are cases of mental problems in general increasing or decreasing?

5: This is a real problem, because you can see many people coming and think that problems are increasing, but in general, this is due to the fact that we do not have a baseline investigation for us to know how things were before. But I think they are not increasing, rather thanks to the increased level of information, people use our services, especially when we follow cases of trauma, we cannot say that they are increasing, especially since even those we see are those who have problems related to our history. We notice that the number is not increasing; rather it is awareness or accessibility that increased.

Q: Others, what do think about it? Do you agree with him? Is it the same? Let us move to the second part. What you have so far told me concern what you see here as employees working for the service treating or helping in these mental health related problems. When you look at the community around you at the place where you live, what do you notice? Do not you receive cases of people coming to you telling you that their children have this problem or so and so has this problem? How do you perceive this in your community? What kind of problems do you encounter in your community?

5: I cannot speak on behalf of others in the community, for example no one has ever come home and knocked at my door to tell me that there was a problem. But I think there are some cases, cases of serious psychiatric conditions where patients walk with funny loads on their heads, or you find them seated under a tree, or very dirty, or on the street. However, even these cases have decreased because of police intervention. When a person is spotted in a place seated alone, for the sake cleanness and security, the police take them to the hospital.

Some few cases can be identified in the city, you can be walking in town and find one. But people do not come to our homes, except health counselors.

Prob: But you have an idea of how the situation is like in the community?

3: I do not say there are no problems related to mental health in our villages. When there is someone who knows that you as a medical Doctor, they most of the time come to you for advice. I often see epileptic patients. Sometimes a person comes to you and tells you that his/ her neighbor's child or his own who half die and therefore requests for advice. I saw many cases of epilepsy in my place.

Prob: You, have you seen such cases?

I have never seen them but heard of. There are some phone calls I receive, and because I know that person with the problem, I help him/her, or when I go to train people in the province, they often tell me their problems in their homes. No one came to me with a child, but some cases I often see, are parents whose children have psychological problems who cannot afford to go to school, cannot live in the same way as other children live. Others present depression; these are the ones who come to me, and after my investigation, I most often find that they are in the category of teenagers who take drugs.

Prob: People in general?

4: Teenagers

1: I often see people suffering from epilepsy at my place, but they take medicine. I see patients with epilepsy and schizophrenia.

Prob: But there are some you may know?

1: Yes, there are.

4: In addition, it is not only here, one day I was working a community mobilizer in mental health in the Southern Province. I noticed many problems during the time we were participating in the community work with the population. Many of these problems were related to domestic violence. I saw that they have many problems related to domestic violence. Because of time constraints, I did not manage to carry out an investigation, but when you refer to what they tell you, you can understand. These are problems based on or related to the way they live in their homes.

Q: I would like to know, according to what you have just said, is there any correlation between domestic violence and mental problems? Is there any correlation?

4: Yes there is, because if someone tells you that he will kill you tomorrow, in this case people can commit suicide, and others can suffer from psychosomatic disorder, chronic headaches, pains in the belly, patients I receive have these problems. So, you cannot say that there is no correlation, but there is. How can you be mentally healthy if you are always in trouble? If someone tells you that he will kill you tomorrow, you can finally commit suicide, homicide or develop depression. Others develop some psychosomatic disorders like chronic headache, pains in the belly, and high blood pressure. In my view, you find that the major problem they our patients have is related to the way they live in their homes.

Prob: We want to know how others understand this issue. Is there any link between the two?

5: There is something many people like to ask, we often see it when we do forensic tests, I do not know if you asked whether the person committing this act of violence or the behavior of violence act is the one who has mental illness.

Q: I need to know, unless you have something to add, if these cases of violence you always see, are found within people with PTSD, do all the people present PTSD because they have been violated, or subjected to any kind of violence in their lives?

5: PTSD is developed when a person has been subjected to violence to the extent that he or she does not have anything to save him/her. Genocide is one of the causes of PTSD, there are other people who can be victims of violence or trauma, and immediately get PTSD.

Q: Do you want to add something from your experience? I want to know, some kinds of PTSD result from genocide and others result from household conflicts. How important are those related to household conflicts compared to those related to genocide? I am not asking figures, which ones are more important in cases you treat. Now we have started talking about the link correlation between PTSD and violence, sorry the correlation between violence and mental health programs?

1: Many patients I have are mistreated at home; this is just an example since most of time they are women, after a long time living maybe in this horrible life, and this culminates into mental problems.

4: In my view, I can divide these problems into categories, for example those I receive have problems related to their family. Based on the fact that they get domestic violence, there are

many cases that are related to intimate violence, their consequences aggravate these problems even though the patients may have had these problems during their childhood. But when you look at the youth, many of them are those who seriously experienced the genocide. Trauma which is manifested or other psychological problems are related to genocide, this is the category of many patients.

Prob: You said much about children and women, what about men?

4: I did not receive many men, that is the reason why I did not say anything about them.

Prob: Others can tell us, which cases are many.

2: In my view, all the people who have faced violence do not necessarily develop PTSD. But most of time, people we receive have chronic headache, insomnia, backache. All these symptoms have psychological cause. I have not yet received a patient with PTSD from any psychological problem before the war and genocide. Many are in this category that have been victims of violence or weakened.

Q: Let us see, we want to know the management of mental health problems in the community, in the families of those patients with these problems, how do you see their management in the community or their families?

2: We often receive patients who have been forced indoors without being seen in order avoid to spoil the honor of the family, but as the situation improves, people get to understand that these mental problems can be treated as other illnesses. With trainings of health counselors and nurses in the Centre, little by little I can see improvement.

Prob: Others, can you add anything on how mental health problems are managed at community level? At family level? How do families manage health problems?

2: What I can add is that families which have a patient with mental problems, let's say epilepsy or any other psychosomatic illness, when we explain them, they are understand, and take care of their patient. We often do this during the consultation.

4: What I can add to what my colleague has just said is that the community intervenes. But the question is how it does so within the limits of its knowledge. When you have a patient, you can take care of him/ her in different ways depending on the knowledge you have. You give what you have. Indeed, the community does not do much because if you see those patients who come to us, most often it tries with prayers; others consult traditional healers, etc.

depending on the community's feelings on the patient's illness. But while talking about the domain of health at the community level, for example health facilities, there is still a lot to do regarding mental health. I always ask people how long it took them to come to us, you find that it took a long time like two or three years taking paracetamol from a Health Centre, they do not have enough skills to know when and how to process the transfer. This is a big challenge, indeed, no technician in the domain of mental health. However, some efforts are being made through discussions, sensitization, campaigns, decentralization of mental health activities at the community level, this is done but we still have some more steps to be made.

Prob: What are these steps in the community?

5: I think people are not informed in the community, unless it is a too obvious thing, it happens that a person starts with depression. You find that the community and families are unable to know what is happening especially regarding mental illnesses as we said earlier. Except for a well-known illness like in the case of a patient who is very aggressive, and in some other situations, family members can know. There is a case I received one day, a husband who had a wife suffering from a headache but he did not know. This went on until she was taken to the hospital. When we asked the husband how it started, he replied that he thought it was the women's caprice. So, you understand that if one does not distinguish behavior change, like if a wife cooks from the living room instead of cooking from the kitchen and the husband thinks it is women's caprice, you understand that there is still a long way to go both at the family and community level.

Q: Let us now discuss, there might be people in the community who have not yet gone for treatment yet they are ill. Let us discuss the reasons of not going for treatment at the hospital even though 90%, if not a 100% of the population, have health insurance. What does prevent people from going to hospital when they have mental health problems? The first is what the Doctor has just said, they do not know the problem they have. There is also something you said earlier that your environment cannot know your problem if you yourself do not know that you are ill, then you sit down and keep quiet.

2: Another thing I notice is like epilepsy, whereby patients are said to be poisoned and taken to traditional healers or when this fails, in the church as my colleague has said. Another thing, although you said that health insurance is accessible to all the people, it is not true because the community health insurance is expensive today, so this can be a factor that prevents people from going to hospital. So people are not able to spend three thousand, indeed if it is

a big family of more than six for example, they are to pay eighteen thousand. Poverty is another factor.

Prob: Is that true? Is the lack of access another problem?

Another thing I saw is that when I go to the field to visit people as facilities providers, sometimes this can be inefficient when a patient gets drugs and do not get better. The question is actually how we treat patients. If a patient comes to me and I help him/ her, goes back to the community and gets better, then when he/ she meets someone with the same problems as his/hers, tells him/ her that he/she had similar problems and got treatment. That is how people market our services, and most often we are not the ones to do advocacy. Therefore, when a patient does not get better this spoils our market. This means that when you talk to someone and tells you that whenever he/ she goes to hospital, they give him a paracetamol and he/ she does not get better, this means that services delivered at the lower level with regard to mental health do not encourage people to go to hospital. Another important thing as highlighted by the Doctor, he said when you recover from an illness, you are boastful; and a dying person never knows the cause of his death. Most often when you do not know what you suffer, you cannot know your treatment. That is why we advise people not to take drugs and finally they end up having a serious illness while at the beginning they merely had insomnia. For example, some days ago I received someone, an intellectual with a University degree, he came to me, and when I was explaining him how therapeutic settings are, he told me, "oh, I thought one session was enough to get better". I told him that it was impossible, that he had to come at least two, three or four months. He said that they were too many. He made a good step of asking, but he still needs some basic knowledge about his illness. This of course means something.

Q: The thing is people do not have some knowledge about various illnesses; do you have anything to add?

1: As my colleague has said, poverty is key factor that prevents people from going to hospital. If a patient is lucky to find where he/ she can be treated or helped with his/ her health insurance, he gets tired by the fact of coming many times for treatment. Another thing is that with a patient having a mental problem and at the same time being responsible for the family, this brings about various problems. Also, once at the hospital, providing medicines knowing that the patient will not eat or will take insufficient meal becomes problematic. And this problem makes them to be abandoned. A patient can come at the first time, but impossible for the second time or until he/ she gets better. In addition, the issue of health insurance,

even when it was given free of charge because he/ she is poor, most often it is not delivered timely. The fact of not having it on time delays him/her to respond to the Doctor's appointment, and as a result his/ her health gets deteriorated, he/ she gets tired, and maybe people in the society discourage him/her. Then, the whole process is stopped. So, poverty is one the factors that prevent patients with mental illness from coming to hospital.

Prob: I would like to know if there are specific reasons why women or men do not come to hospital.

1: What I can add on that is that when people talk about the hill of Ndera, there comes in people's mind an idea of "madness". When you tell a patient that you are going to transfer him/ her to Ndera, in neurology services, he/she refuses to go there fearing that people who will see him/ her at Ndera will think he/she is mad. That is also a problem.

Q: I would like to know specific problems for women and men that prevent them from going to hospital. Are there some specific problems for men and women that prevent them from going to hospital? We earlier said that men do not like to go for treatment, what are their challenges compared to those of women, are they specific to men or women?

5: But my colleague has talked about it, especially when it comes to trauma or depression whereby men have defense mechanism and many of them start being busy. There are some who come to hospital, and you find that he has started developing insomnia, and most of men often start drinking beer.

Prob: They have alternatives at hospital and other alternatives

5: They do, but they act like that

Q: Regarding violence or gender based violence (GBV), why don't people go to hospital for treatment and support when they encounter such problem or do they look for other ways they can be supported when there is any violence based problem, either psychological or physical?

5: These days, serious measures have been taken regarding domestic violence or gender based violence (GBV). These cases are followed up by the police, it does not go through channels of going first to hospital for treatment once there is a case of rape, you find that the case of domestic violence is an exception. Most often victims of violence do not come to us, they first go the police, I do not know if you have ever gone to Kacyiru at police one stop center. That is where they go, we only receive cases with complications of gender based violence.

Prob: So, you do not receive this kind of cases unless there are complications later?

4: Yes, only when they have complications. I think that as the Doctor was saying, those who go to the police one stop center are not many; let us assume if we have two one stop centers like that of Kacyiru, and health centers will help to sort out these problems. It has been noticed from research that most cases of violence are against women based on statistics from patients who often visit the hospital. But when they arrive at the hospital, they do not say that they have been victims of violence, rather they come for treatment of chronic headache; most often it is the Doctor or nurse who can suspect the origin of the problem. Another thing is that we are not in hospitals, so it is not like screening of gender based violence so that we can place it within the package of primary care. Another thing we can add is the context of our culture, most often, going to hospital means revealing the secret of your household. I always say it, when I read some literature, you find that even people working in the medical domain are victims of violence, and keep quiet because of these traditional factors. We sometimes receive a patient who has been in this status for a long time, and when you try to help him/her, you can be surprised to learn that he/she does not accept to acknowledge violence against him/ her. Barriers to go to hospital are the culture and the fact that those who help patients at the lower level are unable to screen the problem. In addition, there are some people who do not know and understand that violence can spoil their lives and think that once they are aggressed, they can go to the police if they dare, but only very few cases go the police, while others prefer to keep quiet over violence with resilience.

Q: Let us continue with the next question, remember we talked about people aged between 20 and 35 years, what do you suggest to be done for health programs available either in the community and centers to be improved?

5: I was wondering why we can plan for these services, why do we need to link or propose this group?

Prob: The why reason we thought about that specific age group is that research carried out by Dr Simberson et al. in mental health in Rwanda has shown that this age group is vulnerable, this is the reason why our primary research was on the youth, bearing in mind that the youth are the future of a country, so our focus was on this age group.

5: You remind me of something, because of the research done that I have read, do we talk about them knowing their age?

Q: Maybe he did not only take that group, he took the whole population, but he realized that this specific group is vulnerable in mental health problems. This may be due to consequences of genocide or PTSD, because he worked on PTSD mainly, but the suggestion we are going to make is for that group, but you can also work on mental health programs in general, there is no problem. So, looking at the prevailing situation in the country, what can you suggest on what we said above?

5: I would suggest a special treatment, because when I look at this age group, for example on average these people are at universities, again, they are looking for jobs, others are still developing their careers. This is a group of people that I think can be helped to continue to be stable. So, I think there should be awareness when it comes to psychological conditions, people should understand what it is and what they think it is, and where they should go. This should be a must since there some who even complete their university studies and start taking drugs, which is ignorant. They should know reasons why people take drugs, whether they have some problems and what else leads them to do so. So, there should be awareness on how these symptoms appear, and the youth can then be guided.

Prob: Others, what do you have to add?

Another thing I can add is that there should be in the community social support to people with mental disability in order to facilitate especially observance of drugs, if they have means to live, most often they do not have jobs, to provide them shelter, and access to medical treatment. If a family member has a mental disability, and his/her family is poverty and unable to get a health insurance, in my view this is a serious problem. There is a need for social support to people with mental disability or in their early stage of illness in order to get early treatment and on a continuous basis. In my opinion, since we most often receive people in need of help but due to the social condition factor that is not favorable and poverty of the patient, all this constitutes a hindrance to his treatment and improvement of his mental status. This means that if a patient with mental disability is poor, social support should be first provided from the Sector level, I don't know, for us to be able to help him/her as well.

Prob: By social support, do you mean health insurance?

1: Either health insurance or shelter, etc. i.e. health insurance, house, and food.

4: What I can add is that in health sector, the referral institution like that one should not only be here because health is integrated in primary life. This should not only be at the level of providing drugs but also at the human resource level, whereby at a health facility we can find

qualified staff who can screen, and help patients to get drugs, because as my colleague has said, we are at the District level, a District is a very big entity, so covering thirty Districts is very difficult. This is similar to the past when all the people used to have their problems handled at Nyanza. It is like someone in need of medical service and walks more than twenty kilometers, when you think of twenty kilometers, cost of ticket, all this makes it still hard. Another challenge concerns the methods to be used in health centers that are not yet well designed, which means there is still a problem. The second point I want to raise as our colleague has said, mental health cannot be improved as long as social factors are left behind. In my view, it is somehow impossible because if someone comes to me and gets all the required treatment but with some barriers of social lives, it will not work out properly; so we need a strong society. As long as we do not have a strong society which will insure that a patient has the basics to live, his/her needs are not satisfied, and this will not help me much as a professional even when I have to transfer him or her to another facility. So, the country's social life should be strengthened. Another thing is that the family should be supported as many problems come from the families; so, the country should help in restructuring the family such that we have a real family.

Pro: What do you mean?

I want to mean that these institutions either the Ministry of Gender or partners should revisit the value of the family. You see what I mean?

Q: I got you but I did not understand well what you meant?

I mean, let me give an example of my family, as a stable family. The government should help families to be stable. Here we need enough psychiatrics and psychologists in referral centers and District hospitals and even at health centers because patients pass by there before coming to us.

Prob: In relation to mental health?

4: Yeah!

Prob: You mean that because families are not stable, they are not fine? You said that in health facilities, it would be better to have staff in health centers like those in referral centers, what do you mean?

4: I mean, we can have for instance mental health nurses that graduated from within, we can also have psychologists, I think the country should see their necessity and make a budget for

that, because if I am in Kigali, when is a patient from Cyangugu going to reach me? Even upon arrival, I won't be of much help.

Prob: Do you have any other suggestion?

2: What I would like to add on the social system, in my view, it does not matter to have an employee at Sector level in charge social affairs, where people with mental problem will go, get money, have a house built, briefly get everything. In my opinion, the Government in collaboration with the Ministry of Health can fund a vocational training center, in the way street children were taken to a centre and taught different vocations, and afterwards they leave the center with skills and knowledge to do some jobs, you understand that it is not the same as giving money, rather you provide some support in some important income generating activities as you cannot always recourse to social systems available at District or Sector levels. The latter indeed sometimes get tired of them and start rejecting them. I think that they can be supported in creating small projects that would help them survive.

Pro: You mean for example what is done in HIV project? Any other comment ?

1: Another thing, as my colleague has highlighted, it is true depending on the mental problem someone may have, the functioning of the system can be a problem. Before teaching a profession to a patient or taking him/her to a course, there should be a program of rehabilitation. There should also be trainings depending on the patient's capacity in order to find something that can help in self-development. But in case this fails, people can find a way to help the patient to survive. Those who manage to recover can be taken to vocational training centers as earlier said to help them improve their lives, otherwise they are forgotten forever.

Q: Is there any other problem you always encounter here that we left behind? Is there any?

1: I think we covered all

Q: Did we cover all?

Q: Are there cases of suicide or attempts to commit suicide do you have here?

1: Especially in depression, some patients do have symptoms.

Prob: We did not talk about it, I have just remembered right now but someone had discussed about it a little bit

4: There is a problem here because the hospital is far, and a transfer to CHUK is not easy; so, the process to go through for you to reach here is not easy, they turn into chronic cases.

Q: What do mean by chronic?

Patients might have attempted suicide, and then were admitted to the hospital, then after they come to us to for psychological support or any other help.

Prob: Do they bring these patients to you?

2: Sometime they treat them at the hospital and bring them to us for counseling and psychological treatment. Last time I met a case of a patient who was first treated at Muhima and later we treated him psychologically. He wanted to commit suicide due to family problem but he did not succeed. He was followed up at Muhima, and it was necessary for us to know exactly what the problem was.

Prob: What do they say as their main reasons when you talk to them?

Prob: Others, you have not yet met such cases?

1: We saw many of them

Prob: Many of them?

1: They come, I personally received one who was the head of the family, he had many problems, looking after his younger brothers, and so many other problems, he was unable to solve them, decided then to commit suicide, fortunately he failed. When this happens following the status of the patient, he/she is taken first at the ordinary hospital for physical treatment, and then afterwards transferred to us. Usually many of the cases we receive are marital conflicts whereby husbands are unfaithful, and have many suicide attempts, or can have a suicide idea, and most of the time after recovering, they give up this idea. Many cases I always encounter are women, I have never seen men; and when men try to commit suicide, they succeed. A man plans well, but a woman can hurt herself or drink some poison which does not kill her immediately, while for a man, he implements his plan and puts an end to his life.

4: Another thing is that suicide is caused by many factors, there some who commit suicide because of history, because they have been rapped, you know, suicide derives from depression, they try but sometimes they fail. The majority are females since they constitute the population we receive, because suicide for men does not fail most of time.

Prob: In short you do not receive them? Do those who have suicide attempts succeed? Many of them are women and their problems were caused by men?

4: Except what we have heard, but when you read the literature you find that many husbands with problems, often commit suicide because of violence.

Prob: I was wondering if there is any other issue not covered here, then we landed on suicide, is there any other issue we did not cover?

4: None

Thank you for your time and contribution, we shall make a report of all recorded ideas so that people are informed on research findings.

THANK YOU.
