# Supplementary material for: Gender Inequality Prevents Abused Women from Seeking Care Despite Protection Given in Gender-Based Violence Legislation: A Qualitative Study from Rwanda
Source: PLoS One. 2016 May 6;11(5):e0154540. doi: 10.1371/journal.pone.0154540 (PMC4859471; doi:10.1371/journal.pone.0154540)
Supplement: S2 File — (PDF) [file pone.0154540.s002.pdf]

SUPPORTING FILE 2

INTERVIEW HELD WITH HEALTH CARE STAFF FROM CARAES NDERA

Date: 16/10/2012

Hour: 03h7-05h20 p.m.

Respondents: 10

Prob: Basing on what you observe in your lifework activities, what are the psychological, physical and trauma problems found among the youth aged between 20-35 years?

7: For me, I observed what is called in English flashback symptoms during the mourning period, where a person starts seeing people resembling genocide killers who are hunting him/her and the person would be trying to hide himself as if it was during genocide. This is what I mostly notice during the mourning period. Another symptom is a great fear and grief which make people weep. Then, there are dreams connected to what individuals have experienced during the genocide, bad dreams. This is what I wanted to say and then let others talk.

6: Another thing that I have noticed among individuals aged between 20-35 who are still at school is that they are generally ill-healthy because probably of the psychosomatic diseases they suffer from which are related to the trauma they experienced. They get easily sick because their immunity because of their immunity system has declined or because they prefer to lead a lonely life as the medical caregiver said, thinking about their history, their problems, thinking about people they lost who are supposed to be helping them today. They fail to concentrate when at school which may result in failure and this situation causes them to lose hope and leads them to depression in connection to the trauma they went through. There are also physical illnesses such as a stomachache, a permanent headache, or joint pains that cannot be explained and even when asked to undergo complementary medical tests in order to detect what they are really suffering from, nothing could be detected. But when you start talking to him or helping him you find out that the main problem is the trauma he has previously experienced.

4: Another thing I can add is that more often those children do not have families. And as has been said they are among those children who have lost all hope. And as there is no other solution they start abusing drugs including the cannabis, and heavily drinking alcohol. Therefore, they would come here several times because they have neither other help nor other way to solve their own problems and they get involved in consuming drugs. These are the possible consequences from their trauma.

Prob: When receiving such people or the youth, how do you differentiate those who have a mental problem due to drug abuse from those with a mental disease due to any other causes? How do you distinguish them?

4: We differentiate them by means of interaction. When we talk with them, we do not start dealing their problem, we rather start talking about their life in the past; how they lived, how they grew up, then we try to see whether it is an illness caused by drug abuse or it is the impact of what happened to him.

4:I think that they have said almost everything but one more thing to add is that people who are in that age range, are used to returning to their past life called the reliving period, maybe it is what had been forgotten. They seem to relive the events that are at the root of their trauma. In addition to what my colleague said there are other physical related issues such insomnia which mostly occur among that category of people. They suffer from sleeplessness because of sorrow, melancholia, and the fear that the medical caregiver has mentioned. They are unable to sleep because they are constantly thinking about himself. And this lack of sleep is a big problem that most of them have. When they get out of the crisis, they would tell you they had not been sleeping for a very long time even for many weeks. It is an important symptom I just wanted to add.

1: What I can add to what was said is that, these people with trauma problems frequently relive the past. Even when they start getting better, these problems can recur especially among people who have no families and you find that many of them keep on having mental problems because they lack social and family support.

2: To support my colleague about social or family support: we often see that such children or young people always change families in which they live. They go because of the problems they have. They do not understand or are not understood by the families in which they live. Today they live in this family, tomorrow they live in that family, they continually change families.

3: A lot of things have been said, but what could be noticed is that there is a factor which worsens the situation of people with trauma problems: lack of assistance when they are at school and have nobody to pay school fees for him or have no scholarship. Such elements contribute to aggravating their precarious situation of hopelessness. So those are the worsening factors and even when the commemoration period arrives, they revive the trauma they experienced during the genocide in addition to other ordinary problems of life like poverty, not having brothers or parents and they eventually that their life is absurd.

This aggravates the situation they live in, they lose hope in their future ss years go by, their trauma becomes more severe but when you talk to them, they would tell you trauma is at the root of all their problems. The genocide and its consequence on their life with no family to rely on are the factors that contribute to worsening the problem.

8: Another problem we always face especially with those children is that today it is difficult to explain to them those psychological issues. More often this goes hand in hand with the general mentality of those who have mental diseases. So the problem is to know what happened to me. In our common mentality, to be called a mentally ill person in our culture is considered as a heavy burden which is difficult to bear. A person would wonder whether he/she has got a mental disorder or something else. Even in the words we use, we tend to separate such illnesses from others but for professionals like us, we use the same term PTSD.

There is a problem for the patient to understand that the symptoms described are those of a person having a mental disorder. In an expression that is commonly used he/she would say:" have I gone insane? how is it possible? Therefore, it is sometimes difficult for us to explain to him/her and then we fall into the trap telling him that he/she is not suffering suffer from mental disorder but only from ordinary trauma.

Another problem we sometimes encounter is really to find words to use when explaining to him in our mother tongue where most of the things are expressed in foreign languages which complicate the way he could understand what happened to him. Another thing I have noticed is that during the commemoration period, you find that more often this event becomes a trigger for illness recurrence in some people who have not been well-prepared to face such a hard period or it is a real problem for us to identify people who are likely to suffer from trauma more than others in order to take care of them.

Those mechanisms are nonexistent and people may come to take part in the mourning ceremony and have a trauma attack and be brought back to hospital. There is a problem of accompanying them in this hard period whereby they revive the events they went through and we see many of them coming back to our service. Even at the end of that period, no other follow-up is done because when we give them appointments, many of them do not come. Those are some of the problems we often face.

Prob: I see that you talked about people who have problems related to genocide, do they have only such problems only or do they have others?

7: Many have got that problem related to genocide which are at the root of their trauma but there are other people who have the problem of having been sexually abused like women in the family and those who have been raped. They also have characteristics of trauma. Another thing I could add is that people who have trauma or who are in state of severe trauma crisis do not like to listen to radio and television programs about the events they went through, instead they like to attend mourning organized in many various areas during the commemoration period. They undergo a trauma crisis and the following day they move to another place and have a crisis, etc. And it eventually becomes like an addiction to commemoration ceremonies.

Another thing I could say is that there is something which is similar to a pathway from a trauma event to a serious mental disease. Nowadays, we receive serious mental diseases like psychotic disorders but I came across a case which clearly shows that pathway. That person went through the genocide, then the period of anxiety disorder which is called generalized anxiety disorders with all symptoms like dyspnea, palpitation, and after he will enter the somatization chronic headache disorder period and abdominal pain for a long time. It looks as if someone is fighting against something which is inside him and which is aggravating. And it looks like someone is treating a wound which is inside him through those somatization symptoms. Then after, when that defense mechanism of somatization is over, it leads to psychotic disorder.

In any case, I have a case of someone who went through all these four phases and it seems like pathway can help us carry out an early screening before PTSD is complicated and we can

diagnose psychotic disorder at the phase of generalized anxiety disorders or we can well treat the somatization chronic headache in order to prevent psychotic disorder which is very difficult to treat.

One more thing which can be very interesting to deal with is those crises. Frequently, it seems as if we wait till the commemoration period when emotional crises occur, when some people engage in weeping and collapse which can lead them to loss of mind and they are taken to hospital for treatment and they recover.

But between two commemoration periods, many people are not well followed up. It is true that we give appointments to many of them but they do not come. But if there could be groups of people who could monitor them between two commemoration periods, this could alleviate the frequency of crises and their complications.

6: Another thing I could add is that people who have trauma are those who have had problems during the genocide but there are also girls or women who are sexually abused. There are also others who are aged between 20 and 35 whose parents are imprisoned maybe because they participated in the genocide.

They may also show symptoms which are more like symptoms of trauma. About that youth, one of them could have a trauma crisis during the commemoration period then, he would ask himself: "why have these problems happened to me?" He/she would accuse him/herself and feel guilty of having had those problems. Mainly, because he has had those problems when at school or where there were a lot of people who know him and he/she would think that it is a crime to be the only one to have had a trauma crisis. Or a colleague of his/hers had the same problem last time but today it is his/her turn and he/ she asks him/herself the cause for that. And this can lead that person to despair because he/she realizes that he/she has no resilience force against the trauma he/she has enduring.

10: On the same point, I want to add something that we find in some persons of that age range. When we say people who faced the genocide we mean mostly those who directly experienced it being aged between 20 and 35 among this age range, we include other people who indirectly experienced the genocide who have either heard of it or seen it on videos or seen its consequences when they came back to Rwanda and then later they developed a trauma. Thus, this is a secondary source of trauma as they manifest trauma symptoms without having been directly exposed to the genocide but because they have heard of it or seen its consequences

7: There are women who were pregnant during the genocide and their babies manifest trauma symptoms. They look like people who have trauma through the eyes of their mothers. They lived the trauma experience of their mother and it is like the generation transmission of the trauma event.

Prob: If you consider boys and girls who suffer more from trauma problems?

7: Frequently girls because of their sensitivity. Girls are more sensitive than boys.

6: According to the statistics established after the commemoration period that show the number of people with trauma we receive every year, it is clear that females represent the larger number of affected persons. As the medical caregiver said, this may be due to the female's sensitivity. But if we would try to explain the reason why males are less affected as females, we could say that it is a matter of self-control which is culturally bound as is said that men's tears flow in their abdomen. It may happen that young men have trauma crises at a memorial site, but they would try first to self-control and hold back their feelings and finally they will end up having flash backs because their strength and defense mechanisms will have decreased.

10: I want to add something on what was said: it is true that females are more affected because they are more emotional. But there are other two factors that should be taken into account to explain that state of affairs. The first one is that statistically, there are more females than males in Rwanda. So the number of females affected by trauma is higher than that of males. We cannot ignore this factor. Secondly even we can do a census of people who have trauma or those who are exposed to trauma, we find that women are more affected than men because most men were killed during the genocide and a larger number of women survived. It is easy to understand why the number of women is higher than that of men. Those are other two factors that explain why more females suffer from trauma than males.

Prob: Why don't you say anything? Do you agree with him? Is it the same?

3: Many things have been said but another thing is vulnerability in one word. I could add that the female sex is emotional. It is simply vulnerability as I could say and children may be included in the group of people who are vulnerable.

Prob: Considering the place where you live and where you work is there any difference between these problems?

7: It is quite understandable that we should receive more people here than where we live because it is here that they bring all people who have mental problems in the whole country.

10: It is what I was about to say, I can say that here we supervise all the situation because we are taken to wherever commemoration takes place. It means that we receive many cases and we receive them here in the hospital because everything is dealt with here. Therefore we receive many cases. There is something that I just to emphasize: living conditions are another factor that is the cause people to have trauma problems.

In our Rwandan society, there are more women who have bad living conditions than men. When looking at the problem of poverty, we find that a larger number of women abandon school, lack homes than men. I do not remember well the year when I found out that the great number of people I had been treating was made up of house workers. Then I asked myself why more trauma problems are found among this category of people? And when I talked with them, I realized that the reason behind is that they come to work as house workers with trauma problems as they have nowhere else to go. In other words, living conditions remain the problem. You find out that if they had better living conditions, maybe they would have a defense mechanism that which would allow them to resist and be

protected from those problems. Therefore, this is a factor which affects more females than males.

6: One more thing to add is about the question that we have been asked of comparing here at work and the place where we live, as my colleagues said here we are more likely to meet many people because we are the referral hospital. But we cannot deny that there are no trauma problems where we live there but as we said it at the beginning, those problems mostly occur during the mourning period. During other months, there are less of such problems. It means also there could some trauma cases during the mourning period but almost all residents from go to attend the commemoration ceremony at the stadium. It is therefore possible that some of our neighbors could have trauma crises at the stadium. Then it is not easy to know that any person living 500 meters away from my house got a trauma crises at the stadium.. We cannot deny that such cases may exist but they are not many as compared to the cases we deal with in our daily lifework.

1: Going back to trauma issues, we talked more about people with trauma due to the genocide and its consequences but other forms of trauma may occur or arise from difficulties one may encounter such as family conflicts which cause family members not to get along well with one another. They sometimes manifest symptoms but concerning these cases, it may be difficult to identify them because they may not reveal things such failing to sleep or having permanent headaches due to family related conflicts so that they could be provided with assistance. Those are the cases that could be found in our surrounding where it becomes challenging to identify such cases as they have poor understanding of mental health, they have to go to hospital for treatment. You would find that they will have been living with those trauma symptoms for a long time which may result in more serious illnesses because those symptoms were kept locked inside themselves.

Prob: Why do not they go to hospital?

1: I think this is a problem of poor understand among some people. Someone could go to the hospital be helped with the situation in which he lives in all possible ways. Instead, he/she feels that he/she has been settled into that problem forever and had no other choice. It is mainly a problem of mentality.

Prob: What do you suggest that should e done in order to change people's mentality?

1: Nothing else can change people's mentality rather than sensitizing them, so I think we should set up some sensitizing programs for ill people we receive here or go to the field to train people about mental health and how to ask for assistance.

Prob: Are there such activities here at your hospital?

1: There are such activities because we have programs of going to the field to do sensitization in schools, in places where there are groups of individuals.

2: There is another program of visiting families of some people with symptoms who have reached us, we go and teach the leadership, neighbors and families in order to help those cases to live a better life in society.

6: Always on the same point, why does a person manifest symptoms but he does not go to the hospital. Sometimes in families a child may have such a trauma problem but they would not take it seriously and would say: “why do you behave in such a way?” Do these happen to you only? Thus they seem to prevent him from expressing his attitude. This can worsen his illness or problem. Therefore, what should be done as stated by my colleagues? I think there are other activities that could be organized to help many cases at the same time, or some groups made up of the persons with the same problem could come and be treated at the same time. Therefore I think that recommendations could be made. We know quite well for instance because the AIDS problem of is of paramount importance, it has become necessary to set up what we call anti-AIDS Clubs which make people better understand how to prevent from AIDS. So I think if there should clubs in school institutions that would help children to understand and to take measures to fight against the remembrance of events that happened to them in relation to trauma .This may make it possible to prevent from trauma disorders especially among those people who are aged between 20 and 35.

Prob: Who should be the first to take action?

6: Perhaps, there should be collaboration between the Ministry of Education and the Ministry of Health. I do not know who could take action first but I think students from secondary school and university could take the lead helped by the leaders who are in charge of that category of population. There will be recommendations but I think that if they are implemented, this could help us in preventing many crises in the years to come.

5: I want to complete what was said, but there is another thing I can say related to males. Males often show the symptom of drug abuse. They may often engage in that behavior because they have family conflicts. And because they do not like to come here and they lack refuge and when they are overwhelmed by problems they engage in consuming drugs. Such cases with such problems are mainly found among the male youth population we receive here.

Prob: Do you receive many people who have trauma problems due to conflicts within families? How many are there people who have family problems?

5: They come but it is not easy to say the number but we see one of the factors which push them to come because people who have family problems do not often state them: these could be due to a conflict between a husband and a wife, general torture, harassment or beating, and any kind of conflict.

6: Because we said that a person who has trauma is sensitive. If a man talks to you angrily, it is easy to connect the problem you have encountered to what he has told you. This may be a trigger for someone to feel worse

10: There is another thing that I said before which I find most challenging in treating or helping these people with trauma who are aged between 20 and 35 years. It is the problem of stigma. We talked about it but I want to emphasize that there are two types of stigma.

Firstly, there is the self-attached stigma due to the way the person with trauma thinks he/she is or considers the mental disease but also how he/she is viewed by society. Like this person

who was given an appointment and told to return later so that we could have another talk with him, he/she would go and decide not to come back so that he would not be treated as a mentally ill or a mad. He/she may stigmatize him/herself.

But as my colleague explained it, there is even stigma that may be attached to someone by the family. So, the family in which he/she lives may prevent him/her from seeking assistance because they do not realize the role that the assistance may play.

You find that it is a stigma they attach to a person with trauma. There is no other treatment than counselling them, explaining to them but I would like to add that research could be one of the ways which may help find a solution to that problem. If no research is conducted to clarify the nature of the problem itself, nothing in what we are doing could be effectively achieved.

Firstly, research is needed. In addition I would like to make a recommendation of carrying out research on stigma itself without mixing it with other subjects. In your research, see what it is, how it is in family, how it is among people who have trauma? Then you will analyze the findings because it can be useful for us, medical caregivers, and for those people who have the problem.

Prob: Are there any people that you receive who say that the cause for their disease is stigma? It is one of the problems they have?

10: Stigma is not the first issue but through the talks that we have with them we notice that they have experienced stigma. "I was prevented from coming or I refused to come and consult you because I thought that if other students saw me coming to see you, they would say that I totally became insane" they would say. Then you discover through those exchanges that a person has attach stigma to him/herself or he/she could tell you: "I did not return to see you because the family for which I work, my mistress told me that this is not a serious matter which should not be harmful to me.” Through those talks, you realize that problem exists. You find out that the problem of stigma comes later while the person has already experienced it but it is not the stigma which is the cause of the problem.

8: Stigma exists but it is not easy to know if a person comes back because it is the stigma which is hindering from his/her recovery or, recuperation or continuation to live his/her ordinary life. The disease that I can call a chronic disease whereby someone can look okay because of he/she is under treatment and he can leave a normal his life, especially when he has a family that supports him/her he/she could recover quickly. Therefore, stigma and mentality are the most important challenges to be dealt with

Maybe what I could recommend that the first strategy is to set up policies, because what we are doing is like a drop in the sea, whereby all stakeholders work hand in hand and put all their efforts together from the top management. We could take an example of other diseases like transmissible diseases where the policies which are being set up are easily understood but we cannot leave this problem of mental health aside. I think that this field could be better addressed.

Concerning research, the recommendations from that research may result in insuring that higher authorities understand that the problem is really the problem. I think that here at the hospital we try to give a good example but when you go to CHUK and see how they think of mental problems while they are the ones who are supposed to be helping us, they may be the cause for stigma because they have poor understanding of mental health. We cannot blame them nothing is done. And sometimes we do not cooperate because when a patient goes there, he/she shows some symptoms and he/she I immediately transferred to Ndera. And you find that stigma is everywhere.

I think that you know that when a student gets a mental problem at school he/she cannot continue to study while nothing can prevent him from studying because he can continue being treated and leading his/her normal life. This is a challenge because when we are treating him/her, the society which is supposed to be accompanying and helping him/her does not take care of him, attach stigma to him/her instead. You understand that he/she will have serious problems and it is difficult for us to treat him/her because we reach a point where there is a barrier and need help from other people but nothing is done.

7: There is a problem of commodity existing between HIV and PTSD. Many young people engage in consuming drugs and fail to protect themselves and end up being infected with AIDS and this aggravates their situations. There is another serious problem: because of much grief the youth try to commit suicide.

Prob: By estimation, how many are there those young people you receive who have such problems and who are HIV positive?

7: Here we have a service in charge of them and they are the ones who could give you verifiable data, because they have statistics of cases of PTSD and HIV/AIDS.

6: If we could come back to is stigma and poor understanding, etc., I think that one of the solutions which can alleviate the problem of stigma or poor understanding is what I can call decentralization or delegating, so that people could be easily informed about these problems we are talking about which are sensitive i.e. which seem to affect individuals. For example if someone is transferred from Cyangugu to Ndera, he/she would think that it is very serious. Maybe that it is his/her first time he/she arrives at Kigali to be transferred to the important Hospital of Ndera, this means that his/her disease is a major one. But if things could be dealt with from the grassroots level, people's mentality may change and they may minimize the importance of the problem and take trauma like suffering from malaria. I do not remember what they are called; I think they are called community health workers who now treat malaria. So nowadays malaria has become a simple disease, it is no longer as serious as it used to be. I think that dealing with mental problems at higher levels cause people to think that it is a very serious problem. If we could improve this situation whereby everybody could get treated, could get a trauma counselor or get an expert in mental related problems, this can help in preventing many problems in general.

4: Another thing I can add is that stigma is linked to people's understanding whereby most prefer to resort to prayers first instead of going to hospital. They fail to accept themselves or what happened to them. If prayers do not work they look for witchdoctors thinking that they

have been poisoned or things like these. They would then delay coming to get help from the service in charge and this may result in worsening their illness or they would come at the last minute when they have no other choice at their disposal wishing to be kept in hospital but if they had started approaching people at lower levels, they could have been given quick help and would have avoided going through such hassle.

Prob: Are there any people who refuse going back to their homes and continue to be treated from the hospital?

4: We do not know but it is possible. There are some persons who may be lucky if it is not serious they start telling you that they got healed thanks to prayers. However , there are some cases of people who come at the last minute and tell you that they went to all private hospitals, attended all kinds of prayers meetings and even they went to see traditional witchdoctors but nothing changed.

Prob: Going back to what you said, what do you think it may be the reason for people not to come to get treated? One of the reasons is that they probably go to other places like praying meetings or witchdoctors or elsewhere than hospitals. You even said that parents or the family may not allow people to go to hospital; others say that it is linked to their understandings which are poor in general, family conflicts. Are there any other motives which prevent people from going to hospital if they have trauma problems?

4: I think as a matter of mentality or as we said at the start. Somebody may have headache or trauma symptoms which may turn into somatic disorders resulting in stomachaches, headaches and paralytic disorders and many other symptoms. Then he/she may not understand that it is a trauma disorder thinking that it is an ordinary illness. He/she could get treated by several different persons but could not get better. He/she could have all tests done with negative results because he/she does not know that he/she is suffering from trauma.

2: Others remain in the countryside or we just meet them wandering around on roads. We found out that most of people with trauma disorders have no more families. Orphans, old women, widows also may be called mad persons and go wandering on roads. It doesn't mean that they have no one to take them to the service in charge for assistance. Another thing concerns those who may be ill and have been rejected by their families because they have caused tremendous problems to the family who may therefore be discouraged from helping him get treated.

6: There are other cases that we see. We talked about someone who may have trauma and his colleagues tell him/him that he is not the first one to experience such problems. There are also other cases of those who have trauma but show other types of symptoms because their body tells them that they cannot be affected by trauma, so they are not allowed to have trauma due to their personal history. Then you will find out sometimes that the problem of trauma looks as it does not exist anymore because the symptoms of trauma that we know from books like flashbacks, event reviving and others no longer exist and have been transformed into other symptoms that procedures and family prognostics are showing.

Prob: Is there any difference between married and single individuals as far as those mental problems are concerned?

7: The problem I mostly met is concerned with the youth, especially among girls who have been sexually abused and who have the problem of getting married late because they bear in mind that every male is bad for having sexually abused by a male. This leads them to get married late.

And others who are already married get problems in the family because they are too angry, do not trust their husband, are too sensitive or do not show any feeling of love and happiness and cause other family problems and these come to worsen the side effects of the violence they experienced before getting married such as the frigidity problem or lack of sexual satisfaction. In this sexual life between a husband and a wife, the latter is not satisfied because of what she went through. These are consequences of trauma in the family.

Prob: Let's depart; you said that there are people who have problems within the families. Is it because they got married late?

7: (They laughed) getting married late can lead to stress and anxiety disorder in future. She may come with somatization of a chronic headache and when you try to treat her you discover those psychotherapy problems that you are supposed to deal with.

6: Let's come back to the question of comparing trauma among married and single people. It is difficult to give a specific description of the situation for each category but basing on what could be noticed among the patients that we received such examples of the patients that I treated. You could notice that females are more likely to have trauma than males. However, a girl may have had trauma in the past when she was single but after being married to a nice man who loves her and let say that this girl was an orphan without any assistance as my colleague said, and after getting married to a man who provides her with much affection and care, all of these could constitute an efficient treatment against the trauma she had been suffering from. There are many different cases that we had which are similar to that one. But on the other hand, if she gets married with a bad man it can be one of the causes which can aggravate her problem because she is likely to transmit to bestow to the children that she will give birth to.

This is true. What I want going to say about this question of the difference between married and single people is that no research has been carried out and there is no statistics on the spot unless you allow us to go and find them. If this happens during the commemoration period we have significant statistics, I don't know how to express it but there are people whose occupations may influence their living conditions or worsen or alleviate their problems. Of course the scar resulting from trauma still exists, but what will the future life look like? How do you see the future? What are his/her living conditions like? Do they worsen the problem as we said or has he/she lost all hope for the future, his/her self-esteem, his/her dignity? Does this help him/her to recover or to degrade him/her so that he/she may go away by him/herself and live in loneliness because he has no more self-esteem or dignity? This is what may cause somebody to stigmatize him/herself mainly due to his/her living conditions. It may also happen that the married person to make a bad choice and lack support which could

be a factor that could worsen her current problem. This makes his/her situation to retrograde considerably. It is not surprising to see somebody who tells you that it is the first time that he/she has experienced such a problem for since commemoration exists. What was it the situation before? He says that the problems he/she is facing make him/her think hard about him/herself and go back to his/her personal history. You understand that this is another important factor.

Since you have statistics, how many are they? He/she would tell you that it is the first time and he/she is aged between 35 and 40. Asked how he/she felt or feels about that, he/she would reply that if he/she had had somebody to help I would have felt all right but his/her life was degrading gradually. You see that everything depends on living conditions, how a person lives and how he/she considers him/herself.

Prob: Briefly, in your day-to-day activities is there any big difference between married and single persons concerning trauma? Is the difference due to their living conditions? If I have got you well.

3: There are many influencing factors that could worsen or alleviate the situation for those with mental scars. This is what I meant when I said that there are no statistics or sizeable figures but the number of married people may constantly increase and one may have trauma or not or may be able to manage it. It depends on his/her living conditions.

10:In summary, even though research has not been done so far, there is a big difference between married and single persons but it is clear that, maybe after everybody has mentioned every case they have received, in my opinion, I see that there are more cases of single persons than those of married . Without getting into details of factors it is clear that if you count, you will find more cases among single persons than among married people.

Prob: Obviously everyone is talking about their experience, the cases that he encounters in the service he works in.

7: But the findings from research show that vulnerability among single persons is very high in terms of trauma. Trauma affects more single persons more than married people. It seems like getting married is a protecting factor.

Prob: Moreover, basing on what you observe in your activities, where do the youth aged between 20 and 35 years who have mental problems go for treatment?

7: When you talk to nurses who are in districts, they tell you that there are cases of people who go there to get treatment; at the psychosocial service they tell you that they admit such cases, and even here we receive such cases, at Icyizere Center they have such cases. So it will depend.

4: They go to their nearest hospitals where they get assistance. Many people go to prayer meetings, to traditional practitioners' but most people go to their nearest hospitals.

10: That's what I wanted to say, practically among those who have trauma problems there are some who have information about where they could get treatment such as hospitals and go there, others go to local grassroots health facilities such centers, district hospitals then the

district hospital can bring them at the reference center. But among those who have no such information, as has been said, there is a part of them who do not get treated and we think that even till now they still have those problems but they have not got any assistance yet. There are also those who go to prayer meetings and till now they have not got treatment of their real problem. There are also cases of those who are being given medicine such paracetamol against headaches, for whom investigation is being done to see if they have typhoid or other diseases but they have not found yet where the problem is. It means that there are different places where people could go and get assistance depending on the problem they have.

Prob: Is there any particular person that they consult?

10: There is what we call a trauma counselor. There are people who got trained in what we call counseling in traumatism. They live within the community: one part of them who have been trained and have expertise will go and help those people. There are also other people from RCT Ruhuka. RCT Ruhuka is specialized in helping such kind of people. Then those people may go there but I cannot specify their names or their places of residence but they may exist.

Prob: Are those people found in all villages?

People who have been trained in counseling with the aim of assisting people who have trauma could be found in many areas of the country,

Prob: Have they already brought you some persons from villages?

10: I don't know whether my colleague may answer if they have seen any trauma cases in need of assistance brought by trauma counselors or people from RCT Ruhuka. I think there may be some cases. Frequently counselors send them to district hospitals, health centers. They would come here after consulting different places. But, at least they have been told by a counselor where they could get assistance.

3:I mean that even at hospitals and health centers there are cases with trauma or other trauma symptoms they have tried to deal with and have been unable to. Those cases are then brought to our hospitals either by RCT Ruhuka or by many other organs. So we have such cases.

Prob: Are there counselors in health centers or is there a counselor for every health center?

3: I cannot confirm that there are in all heath centers but there are in all district hospitals.

10: In health centers, there are people have been trained in mental health related areas and work there.

All: yes

7: Now there is a module prepared by the Ministry of Health which aims at training those community health agents in how they could provide assistance in terms of fighting against AIDS and malaria and at the same time helping those having trauma and psychological disorders. Thus this will contribute to helping the community at large.

PROB: What is the difference between young men and young women in terms of seeking treatment from health services? How do they differ in terms of seeking treatment?

6: I can say that the number of those who seek treatment is still low; we do not often see many cases of patients who come alone, because many patients that we receive who come for the first time, I mean new cases, come when in crisis. Few cases come alone without being accompanied. This factor of being accompanied shows immediately that sometimes seeking treatment is not from somebody's will be it young man or a young girl. It implies that somebody has urged the ill person to go to hospital. The boy or the girl will then come accompanied either with his family or the police. It may be difficult for me to say that there more young women who come to seek treatment than young men. But we said that the number of females is higher, it is possible that there will be more women who go the hospital and state their health problems frankly. They will then receive more assistance than young men who try to refrain themselves and try to hide their weaknesses in front of others. Because of that, it may take them a long time to decide themselves to come and get assistance. This is how I can clarify that point.

3: I could add that that statistics could be of some help. We could say that they might be a lot of surprising factors. You could wonder why there are only 5 husbands and 10 wives who have come to hospital, is it because there are 5 men in the community who have not come. But all of these require a detailed study in order to know the patients who have come to be treated and those who remained at home compared to the people that we were waiting for. Of course we consider the number of people we have received but how about those who have not come? Are they sick or healthy? I think that is too long but we could see the number of people we have received but to make data analysis requires us further research. We need statistics estimates to know whether the number of patients we have received is high or low based on a given indicator. But the number of people we treat is higher than those who are ill. This is the estimation that reflects the reality.

PROB: Seeking treatment is a personal matter. You have talked about a person who is ill and is still in the process of seeking assistance, is there anything you would like to add?

10: I understood seeking treatment in terms of frequency such as how many females seek treatment compared to the number of males. Without considering statistics or any other factors, one could say that that there are more females who seek treatment than males. Looking at statistics, you may probably find that around 10 women out of 8 men come to be treated, so there are more women than men, I mean as far as the trauma issue is concerned not in general. Probably also there are more females suffering from trauma than males.

Prob: We have talked about how persons with trauma should behave. Let's talk about how they and families should behave when faced with that problem.

8: In terms of trauma? To many people, when we ask them when they started having such problems, you find out that they have had them for some time. When you continue investigation, you find out it was not easy for the family in the beginning as they did know what to do, whether it was an illness or not as others said. Many people start thinking that the person has been poisoned. They try to relate the problem to the work the person does. By

mentality, others may start resorting to prayers to see whether there could be any tangible results; still others may go to trade-practitioners. The lucky ones will get advised by their neighbors to come to hospital. The family has a challenge of knowing what the problem is and what could be done. And they spend their time pondering over that. In a few words, we can say that it is not easy for the family to understand what has happened before they could to take the relevant decision to go hospital.

6: I think that a person is not affected by trauma just like that. More often, there is something that I may call a trigger, either he/she is watching television or listening to the radio, maybe listening to others' testimonies or to songs which revive the events he/she went through in the past. So those factors seem to be like a trigger that causes him to manifest the signs we talked about. Therefore, this may happen to people individually, such as young men or young women who live alone or in hostels or somebody who lives alone in a room. How will he/she behave? Well, I think there is no other way except that when his/her colleagues notice the signs of the trauma they will promptly offer their help to him. If the same situation occurs when the person is with his/her family, most of time he/she is taken to people who are empowered to offer help to those suffering from trauma problems. But as many years go by, more and more are getting better understanding of mental health thanks to leaflets and brochures which explain how to help persons with trauma. Because people are gaining good understanding, they would start assisting the persons with trauma themselves and would bring them here when they fail to. When the patient is becoming aggressive and attempting to run away or breaking things, they know how to calm him/her down. We cannot say that the crisis will not occur again but at least neighbors and family members know how to handle such cases and sensitization has had positive effects on people's understanding of how to assist persons with trauma problems.

Prob: May be there is something you would like to add to this, is there any shame in a family having a person with a trauma problem considering what you could observe in your lifework ?

4: There is some shame; even the so-called intellectuals feel such a shame while they are considered to be the model to the illiterate lower class people. They will go to a number of private health services for fear of approaching to services entitled to provide for adequate assistance. Families are also shameful and when the patient has another trauma attack again he/she would feel shame and say: 'what would somebody think if he/she sees me entering there? What would he/she think I am going to do there? They would say that I am mentally ill. Because of the feeling of shame, they would prefer to go to other places than Ndera which deals with people with mental or psychological problems.

10: There is shame in the family but I want to distinguish this problem of trauma that we have talking about from other general mental diseases. There is a lot of shame especially for the family who has someone who suffer from a general mental disease; there are many different mental diseases more than people who have trauma mainly because they are totally different both in terms of symptoms and how these people could be taken care of. A person with a trauma problem lacks sleep and has grief as we have been saying it, but he/she does not kill or eat rubbish or go naked. So the types of shame are different. Let's get back to our topic of

trauma. The lower the level of trauma, the lower the level of shame. But when it reaches a higher level, the family feels shame.

Prob: What about the friends of someone who has that problem?

6: I think that we could relate this to how Rwandans view the mental health issue. We can probably say that as years go by, people are gaining better understanding of it but we cannot say that the situation is satisfactory. We often see cases of young lovers, boys and girls, who separate because one of them has had a mental problem. Today I received a patient who has got the same problem: now he keeps coming to see us because his friend has abandoned him since when she knew that he had a mental disease. They were about to get married but now all the preparations have stopped. It means that the friend felt shame as it is not easy to go with someone and introduce him/her to people who already know that he used to be mentally ill. This is linked to the Rwandans 'mentality who think that a mentally ill person is the one who is used to fighting, going naked or who shows other symptoms that characterize people with a mental disease that we meet on the road.

Surely, they may not know that there many different types of mental diseases, and all mentally ill persons are all put in the same category. And this may lead to the increase of shame. "How will they take it when I tell them that this is a friend of mine while they already know that he/she has had a mental disease? You feel that your dignity will be affected by having such a friend, living with him/her and wishing to marry him/her when he/she has a mental problem. They are quite different as my colleague said it, because this problem of trauma it is something which is known and accepted in our society in which we live because of the history that we have been through. For sure, shame could be less depending on what someone could do in front of his/her friend has who has a mental problem.

8: Another thing I could say about this friendship issue is that there is friendship which will lead to marriage. There may be shame as you may sometimes want not to reveal everything immediately to your future partner. It becomes difficult if you doubt whether he/she would agree accept to marry you with your mental disease. You would then refrain from telling him/her about your disease. It would even become worse when your partner learns it from elsewhere because you felt shame to be frank with him/her. The lucky people will decide to tell the problem to their partners and the latter may accept the situation not. So various strategies could be used. I think that you should tell your problem to the partner you live with who in turn will give you advice.

As far as ordinary friendship is concerned, if I have for instance a class friend, he/she will be the first person to be informed of my problems as we usually tell each other everything we do. So I think I would immediately feel free to tell him/her my problems such as my lack of sleep and my friend could advise me about that.

I would like to add another element to the issue of shame and trauma in the community in which people live. A genocide survivor could be living alone or with few relatives. This survivor may still be living among those who tried to exterminate all his family but failed. Although he/she has a trauma he/she would do all he/she could not to show it to them. This is the problem that some people I saw or I talked to have. "What would do they say? I would feel

shame if they knew about my problem?" The survivor would then keep the problem to him/herself and the situation will become worse. I could call it shame as the survivor is hiding a problem that he/she is enduring so as not to be the laughing stock of his/her neighbours who otherwise would wish him/her to have trauma. Many people we interviewed told us that they first tried to handle their problems by themselves and hide it from the public. I think that it is shame that is at the root of not showing problems and not going to hospital for treatment.

Prob: Based on your day-to-day activities, what are the major challenges those people face mostly which prevent them from coming to hospital?

4: They face many challenges. There are some people who live far from the health center and they are cannot to come and get treated. Others have no financial means, no mutual health insurance because they have not enough money to afford the mutual health insurance so as they could easily have access to health services. There are also the poorest that remain inside their homes because they have no means at all.

1: Other people could have medical care but are not followed up. Persons suffering mental disorders which are no more just trauma problems need to be closely monitored and the person in charge of this category of patients finds the follow-up as the most challenging issue

Prob: Why is the follow- up difficult to do?

1: These are the problems of little assistance which is provided because of lack of adequate financial means to help people to accede to health services. The patient's surrounding does not either facilitate him/her to go on that follow-up process. Some people still have the mentality that even though they went to hospital and got treatment, they may continue getting treatment from the nearest traditional medical facility or they may go to prayers meetings as they live with neighbors having the same mentality.

Prob: Do you mean the follow-up at the hospital level or at the family level?

1: They may be related. A patient may be getting treated for the chronic diseases that he/she fail to come and take medicine or talk to the caregiver to how the situation is evolving. People living close to him/her may not be able to ensure him/her the follow-up. I think that treating a mental disease is not just a matter of giving drugs only, it is global: at the social as well as at the psychological level. All of these should be combined for a patient to be helped efficiently. But in some places there is no one who could facilitate him/her to continue that follow-up.

3: I will complete what my colleague said. There are many different elements in the follow-up process. If a person comes, is given assistance, gets better and goes back to the community society, there are other things which constitute obstacles: as he/she should come regularly, he/she may fail to do so because of he/she has no financial means to pay for transport or nobody to take care of him/her, then the illness worsens as he/she has no the family to support him/her. Another obstacle is the hospital, we has no means to visit patients at home. We give a transfer to a patient so as he/she could get treatment from a facility near his//her home but we are not sure he/she has gone there or not. It is true that we could get information from the hospital but that is not a long-term solution. The problem is that those

patients end up coming back to us with a more serious mental problem than just a trauma he had before.

So we try our best and sometimes go to the field in the community to discover that that patient has no relative and when given an appointment he/she does come. We have no capacity of paying visits to patients at home in order to know why the patient did not come. Most of the time, he/she comes to hospital when his state is very serious because of all the elements mentioned above.

10: It's true. I see challenges in this way; the first one is mentality as we have been talking about it. Mentality is a big challenge at individual and community level in general. The mindset which is still poor remains the challenge. Secondly, the Rwandan culture and here I want to focus on individuals: we Rwandans want immediate results. The nature of this problem itself is mentality-related. A disease requires to be treated for a long time. If it is an illness which requires to be treated for a long period it is a challenge because a person with trauma may come once or twice and then say that he/she has recovered.

And when he/she is told to come back five times, ten times, twenty times, thirty times he/she finds that it is also another challenge. This mentality related nature, the problems of poverty, family and community support and the distance to cover are challenges that hinder people with mental problems from getting appropriate assistance. So far in Rwanda there is no top-bottom well-structured network system of helping people with mental problems. This is also a challenge especially when doing the follow-up as it has been pointed out. That means in a well-structured network system, somebody is sent to follow up the out-patient who is at home. As a recommendation, well organized system should be put into place to help people with mental disorders especially by focusing on trauma problems In that system, if well set, a person will be in charge of monitoring if such patient has come to hospital or not. If he/she realizes that the person has not come, he/she encourages him/her to go to another place and the process continues until person feels to be in the system that he/she must not be late or should be assisted at any time or respect the appointment given to him/her.

Prob: Are there any challenges specific to young men and young women?

6: Maybe before I get to that point of comparing challenges specific to young men and women, I think there is another element we haven't talked about: taboo as regards trauma and mental problems in Rwandan mentality. People do not reveal that they are ill or the illness they are suffering from, in addition to their poor knowledge of how to get treatment and to describe symptoms of their illness. I mean those who come alone.

We have said that as years go by, trauma symptoms also go on taking other forms and this is one of the challenges that we are facing. A person may come with other types of symptoms so that it becomes difficult to establish that he has a trauma problem. I would like to come back to challenges or to our limit as workers in this career. In order to know that a person has had a stomachache, a headache for two weeks has failed to sleep and has a trauma problem, you need to see him/her at least three times. Then you would detect the problem and start dealing with it, a process that should have taken place earlier at the grassroots level. This is the limit we have here. This is a reference center which receives many people and we have a

time reserved for each patient which does facilitate us to quickly discover somebody's problem. Therefore, sometimes due to that challenge, patients may not be treated "properly" because the problem has not been well identified known due to the patient' mentality or his compliance is not easy because the drug given has not immediately solved his/her present problem. That is why a week later he/she would go and see another caregiver if he realizes that his health is not improving. This is the time you see a patient moving from one caregiver to another looking for people who can easily help him.

Thus, I suggest as a recommendation that everything should be done to establish system my colleague put it including some form of decentralization so that a person is able to be received by people who have been empowered to solve his/her problems from the lower to the higher level, namely at our level. In this process, the problem could have been identified earlier and when it reaches us it could be handled properly as the preliminary work will have been done at the grassroots level in that system.

Prob: Do these challenges differ from married people and single people or are they the same?

8: Before I talk about married and single people, I would like to emphasize the fact the culture is the most challenging issue as my colleague said. It is a matter of openness. In our culture it is not easy to trust someone and tell him/her. It will even take long to professionals to create a climate of trust so that a patient could tell you all about his/her life.

I found out that with girls it is different. We who help them, we find that when it is a boy helping a boy it is easy, perhaps when it is a girl helping a girl it could be easy but you find that it takes a long time for girls to tell us about their lives. Moreover, I found that it is not easy due to the events a person has gone through. If there has been sexual abuse, it will very difficult to talk about it and because of culture the person will keeps it to him/her, then it will take a long time to get the story out of him/her.

Even with married people it is the same. You find that talking about the sexual act mostly for those who have been sexually abused is not easy and it takes a long time for them to tell the story. From experience, I think there is a difference. To help those people, you understand that there is a need for using psychotherapy. But in our culture few people acknowledge that verbal interaction can heal and that is why they do not come back when they are given appointments because they do not understand how conversations could heal them instead of being given medicine or using any material or equipment to consult or diagnose them. There are some people who would ask us: "you have not x-rayed me, what is my illness?" This is a mentality and culture based challenge.

Prob: No one else who has anything to add?

All of them*:* Nothing to add*.*

Prob: If you could set up services in charge of people who are aged between 20 and 35, who have mental problems without considering the challenge of financial capacity of the center, what is the solution to those mental problems of those people we have talked about? What are the measures that could be taken?

6: I did not get the point but it is maybe the services of caring for those people. Then what is the problem? That's what I did not understand.

7: Establishing counseling services in all hospitals. Those services exist here. There is a service for children, adolescents so there is a unit of the youth and there is a service that receives others and a service of control for people who are aged between 20 and 30.

6: I think that if the center had the capacity, it would be convenient to build a service which would in charge of helping those people from that age range; this center could resolve many problems, because the age of 20 is the age when people are getting out of adolescence and young people start consuming drugs which is one of the reasons they suffer from mental diseases. Therefore I think that, without considering the capacity of the center, there may be some donors who could help in implementing services aimed at helping those people. I think that qualified people could be hired in order to cope with the problems that are arising today. If we go back a little bit to that problem of trauma and mental disease, I think that one contribution that could be taken into account is improving the way of those diseases or problems are taking care of because you told us about the inadequacy of the capacity to deal with those problems. So there the capacity, we could see what is needed so that people could be successfully treated. I think that we must identify the problems? If we take an example of the problem of trauma, what are the problems which occur today? In which age range? Me, I can talk about the problem of drugs.

10: Me, I have understood the question in this way: if we establish a specific service to care for people aged from 20 to 35 who have the problem of trauma, although this center takes care of all people including people aged from zero age to any age, what problems could that service solve? If it is what I have understood, I think that one of the problems it can solve is that of preventing people from suffering from trauma to mental diseases and people could encouraged to be well treated and cases of complications of schizophrenia or other diseases could be prevented. There may be what I can call decentralization which could encourage people to be better treated than what they do in general. It may encourage those who are afraid of coming to dare to come in a great number. That is a contribution that made by that special service if it could come into existence.

8: Another thing to add is that the reason why we can focus on that age range is because people of that age range are very active. Maybe they are the power of the country. I think the aim of this service may be of following up those people. It may also receive other kinds of patients but it will particularly deal with the cases of people studying in secondary school, at university, and those who are newly employed.

About the prevention you were talking about, it is we follow them up so that they could continue to active. Some of them will get married. I think the specificity of this service is that we do follow-up of these people and then we bring them here. Maybe there would be a need for sensitization for those people to know that these services exist. Or we would set up other ways of recognizing them by using the community health workers who would identify them for us on the basis of given symptoms and bring them to that specific service but the service must be enhanced so that the person could be followed up.

Another issue is that we see those people of that age range but we do not see them any more during mourning period and we know where they are. Some are in schools, others have not had a chance to go to school, and still others are doing vocational training. You find out that most of them are engaged in drug abuse and the most of them are orphans. So what are the strategies to help those persons so that they could regain hope and have individuals who will be able to work in years to come.

3: I think that my colleagues said a lot, but what the service could solve the problem we face during the mourning period. We usually receive people in any hall of any infrastructure we have and they would have the feeling of being in a hospital reserved for persons having mental problems. He realizes that he is not a mad person, allow us to use this term even though we abandoned it. It is the term used outside to specify such persons with mental disorder. Then we meet such a person in April during mourning period and we know his problem, we have talked with him, we have assisted him and he/she has got better and has accepted his/her problem. But where does that person live a year later? Now I propose that the service should exist and do the follow up of those people we have identified. We provide statistics everywhere, to the ministry, etc. What happens next? We could suppose that it is for the first time or the second time that such a person has a trauma attack, maybe he/she may come back to us five times gain. Then, what could we exactly do for that person between two commemoration periods. So it is like a vicious circle as he/she continues to bring you the same problems which may be getting worse. So if there were that such a service, which would be separated from the psychiatric hospital , the person would feel more free to go there because there is a daily service and consult and to talk a qualified staff and later he could leave. There is a colleague who said that those people from that age range are dynamic, study, work, prepare for their future life.

Instead of carrying that etiquette that he/she is always in hospital, his image would change from outside people's mind. He/she would be going to a given center and meet a counselor at a given time and go back to his/her normal life. We could suggest that the period between two commemoration periods be assessed to see to what extent the assistance provided has been fruitful. Has the situation improved or worsened? I think that there is a gap. I cannot say that nothing is being done at the community level but it is still insufficient, it is like a drop in the ocean, but the persons in the service, the people totally devoted to service of the patients having mental disorders, I think they would encourage them to go and get therapy, to get appointments and later to go back home.

That's it. Because there are people when given an appointment would say: "what if anyone sees me entering the psychiatric centre? They will think that I have a mental disease or I am mad".

Now we have a center which is at Icyizere, but people do not feel it as a psychiatric centre. It is a hospital, yes where they refer us to but it is a center whoever could enter with his/her car, and even if a relative gets out of it, nobody knows what has happened. Because it is a center which takes care of people who consume drugs and other things but it is a specialized center. and I think that service could help people to go and be consulted instead of staying at home.

That is what I think. Let it be a day center so that patients won't have the feeling of being hospitalized or where the so-called patients with mental diseases are followed up.

Prob: Is there any other problem you have noticed in your work that we did not talk about? That you think we could talk about which is related to this disease that we were talking about?

8: About the problem we were talking about, it is clear that effort has been made in terms of training people who work in this domain but there are still few social workers. There is a need for such workers. The number of doctors specialized in psychiatry is still low. We still have few nurses and there is a need for more nurses who could go and work at the grassroots level.

As far as psychotherapists are concerned, I can say that we have some but they are not employed because I do not know a number of people who have graduated but you find that many of them are not employed in that domain, why? because there are no structures where they could be needed. Psychotherapists could work as counselors as well. In secondary schools, they do not understand the importance of hiring a psychologist in their staff who can help those students who have those mental problems. I think that training is needed but "what do you train these people for?" "I will need them." We have such problems as we said in all sectors but measures should be taken to set up a policy which establishes the needs but without forgetting to use our available resources.

1: Another element we have forgotten to mention is cooperation among sectors which have a link with people who have mental problems. We have many stakeholders: there are patients who are brought by the police but you find that the working relationships between us and the police are not determined. It could be better there were a close collaboration between those people who bring patients here and medical caregivers. Lack of collaboration between organs and hospitals could lead to challenges in the follow-up process.

Prob: What must be done so as to improve this collaboration process?

1: I think strategies could be set up if they could all meet and assess the situation but the best solution would be to establish a network system with a place where they could be meeting to discuss the problems and the challenges they face in their lifework.

2: We noticed that bad living conditions are among the factors that influence the trauma in people. I think that this element should not be forgotten. Even though a psychologist or another person who works in the mental health domain may come and help a patient but his living conditions which makes him have trauma attacks over and over again. Because he/she is homeless or has no food he/she will always have trauma attacks because it is not easy to assist him. I think what we could do for such a person is to help him/her plan for his/her future life and I think something should be done especially for persons with trauma.

6: Another thing that I would like to add: my colleague talked about collaboration among sectors, it is true and necessary that the district and the police should work hand in hand so that the patient could be treated. Right, I took that example in order for us to understand better but as we have been saying, one of the major problems we have is the problem of the follow-up.

He talked about patients living conditions but the follow-up is also a problem. We know that we have a patient, he/she has spent many days here but “where will he go when he/she leaves here?” especially when he/she has been brought by the police, for example. There are also financial problems in the hospital which make it difficult to organize on field visits and see the patients. How do they live? What are their problems? Those we talked about stigmatization, etc. how are we going to explain it to neighbors if we are not able to get there? I cannot say that we do not do it, we do it but if any other capacity could be found so that all in needs could be visited; it would one of solutions which can help in treating health mental diseases.

Thank you.
