# Supplementary material for: Gender Inequality Prevents Abused Women from Seeking Care Despite Protection Given in Gender-Based Violence Legislation: A Qualitative Study from Rwanda
Source: PLoS One. 2016 May 6;11(5):e0154540. doi: 10.1371/journal.pone.0154540 (PMC4859471; doi:10.1371/journal.pone.0154540)
Supplement: S3 File — (PDF) [file pone.0154540.s003.pdf]

SUPPORTING FILE 3

INTERVIEW WITH NURSES FROM KABGAYI HOSPITAL

Date: 10/10/2012

Hour: 10h45'-12h13'

Respondents: 6

Thanks. Let's start our interview. We are going to talk about the youth who are between 20-35 years old.

Prob: In your day to day lifework, what kind of mental, or physical or psychological problems do you observe among the youth aged between 20 and 35? Do you get the point?

4. Repeat again.

Prob: In your day to day lifework, what kind of mental, or physical or psychological problems do you observe among the youth aged between 20 and 35?

4. In the ARV service where I work, the trauma-related problems that we especially encounter concern the youth who are under treatment. They sometimes stop taking medicine and say that they do not want to take them anymore and you notice that the child is engaged in self-reflection. When we talk to him, he says that he has no future. He likes playing football, but a teenager who is seropositive has no occasion to go out to play because he is HIV positive. He says that maybe he will not be a man like others. That is why you find him behaving badly. Another young man would say that he will no more take medicine because his fellow children may laugh at him. These are the problems we often face. Another young man from secondary school would say that he no more takes drugs as he is at school. I am not sure that you can class this in the category of trauma but it is a trauma because he has already started self-analyzing. When he was younger, he accepted to take drugs. Married adult people that we have may have trauma because of their serological status and you find that this is not due taking medicine. But little by little, when these individuals including intellectuals get used to their serological status, they first accept their condition and later on you notice that they are suffering from a mental problem. We work together with people from psychological health services. There are some cases we transfer to Ndera but when they come back, they still have psychological problems. You find that adherence is good, but when they are sent back to Ndera, they still have the same problem.

Prob: If I understand you very well, are there people who have trauma due to the fact of being HIV positive and not because of drugs who cannot tolerate their illness?

4: There are some who cannot tolerate their illness; they used to be healthy and suddenly they get mentally ill. Then we transfer such cases after having noticed that their illness have become

critical. We ask ourselves if that is due to the drugs they take or to an existing illness they have. We do not do a follow-up and when such a case is transferred to Ndera, we think that the case has been taken care of properly and we can conclude that it has not resulted from HIV. These people could talk to you and because of their sound ideas you could think that they have recovered from their illness. But when it is a younger person, we cannot know whether that is due to HIV or family problem.

Prob: Do you think that those who stop taking medicine when being at school fear to be seen by their fellows and not because they have trauma?

There are some who do not have trauma but who fear to be seen by their fellows. But even when you counsel them, you see that they keep on feeling anxious. And you wonder whether that is due to the fear of being seen but because there are other students who continue taking medicine, they accept to take medicine. I do not know whether I could classify this in the category of trauma or not.

Prob: You said that trauma may be caused by HIV. What kind of people from other services that could have trauma due to other causes than HIV?

2: In the gyneco-obstetrics unit, there are cases of minor girls who get pregnant and you see that both the minor girls and the family do not know how to deal with such a situation, so you notice that the child is traumatized. There are traumatized cases among minor girls who get pregnant.

5: Those minor girls who get married are aged are between 20 and 35.

2: There are even some who are not minor but who get unwanted pregnancy. There are even adult girls who could get unwanted pregnancy or they get pregnant in agreement with their partner and are later disappointed by their partner. You could see a girl coming to hospital being some months pregnant and you wonder why she did not come earlier so as to be helped but she comes when she is already disappointed. Even when she comes to hospital, she wants to accuse the boy who made her pregnant. You wonder why she did not do so before. This means that her trauma seems to be due to disappointment.

Prob: Now I am wondering whether she comes to the hospital for pregnancy consultation or trauma reasons seeking help from the hospital?

2: She comes for pregnancy consultation purposes or legal purposes in order to accuse the boy who made her pregnant.

1: In addition to what my colleague said, even though each case is individual but there is a case that came here not for consultation purposes in the gyneco-obstetrics unit. I do not quite remember how old her pregnancy was, but she requested to have an abortion. I do not know if she came for consultation because she had an unwanted pregnancy but she came requesting to have an abortion which was her primary intention. So she was transferred to our service. That is another case.

7: Another thing to mention is that the gynecology service is not often visited by adults aged between 20 and 35 but there are some who are not happy with the sex they give birth to. And

during interactive care, they would say: “Doctor, which sex did I give birth to?”, “You gave birth to a female baby” he replies, “Throw her into the bucket” she requested. You immediately realize that she is not happy with giving birth to a girl and would say that she already has five girls and that her husband told her that if she gave birth to another girl, she would go back to her family. “Where shall I go now? It would have been better if I had not come here,” she says. You understand that she has not an exaggerated trauma but there is a case of trauma.

Prob: Are there many?

7: There are not many. You find that when she gives birth to one sex, the husband imposes her to give birth to another sex. Meanwhile, the husband also invited to come here and get counseled. More often, this happens to women who give birth to female babies. “I do not want her. Keep her with you. Or throw her away or do whatever you want with her,” she says.

Prob: You who work in mental health service, what kinds of trauma do you notice?

1: In the mental health service which receives individuals with trauma problems, I can say that there are many different types. The trauma which mostly occurs in Rwanda is related to genocide during which someone lost many members of his family. We receive traumatized people including genocide survivors and those whose parents participated in genocide. There are also few cases of students and children who show signs of trauma especially among the youth who go to school. Although they are aware of the need for medical care, they just do without it.

Therefore, there are cases of genocide orphan survivors, cases of whose parents are in jail, cases of whose parents are doing activities of common interest (TIG). We have cases of those who have tried to escape the Gacaca Courts. There is another section of youth who show trauma disorders called psychosomatic that is related to their identity.

Based on that, that person is then classified among that category of children born from unwanted pregnancies. Those signs mostly appear during the adolescence period. We have many of them and I think that there is a need for sensitizing the female population on having the address of their sex partner when they engage in sex affair by accident or by consent. It is not easy because of the weakness of the human nature but this could be done as is the case in developed countries. It is said that she who gives birth to an illegal child must not take charge of him/her. At least, knowing the partner’s address may prevent identity disorders observed around here as it is sometimes necessary that a young mother tells you that she does not know the partner who has made her pregnant because of the culture of hiding such kind of information. She may also know him but unable to trace his address. In this case, children are the most victims of such situations.

Prob: What do you mean by identity?

1: To know the family of the father of the child. She may know him but the latter could not recognize the child. It is as if women have not the right to indicate the person who has made them pregnant. I do not know if it is a shame or a matter of culture. But there is a great number of people who suffer from such state of affairs.

5: This is a serious problem. Imagine a beautiful 20 year old university student girl, who does not know her father and says to herself: "I long for knowing my father, getting to know each other even if he does not want to care for me."

1: This is a prevailing serious problem among many people. If you do a research, you will find out such many cases. If possible, conduct research on that.

Prob: Do you mean that you receive those children who do not know their families?

1: When they become adolescent, they face the problem of not knowing their families. We have a case of a 35 year old married mother with one or two children who had such kind of trauma due to constantly thinking about her parents.

Prob: She did not know her parents.

1: She was married and one day said that if she had parents, she could be visited by them like other married girls. I realized that she was shocked when she arrived here; she told us that she thought about her father but she did not have a chance to see him.

Prob: Nothing else? No other cases that you receive which have problems caused by other things?

5: As for me, we used to have some cases when I was in the emergency service. It is not easy to know domestic problems but a wife who would come and tell us that she has a headache.

The results show that she is healthy, and we give her place to rest, we counsel her, then we transfer her to the mental health service, and after we realize that she has some problems with her husband. Maybe she is mistreated by her husband.

You will find out that when there is a misunderstanding in the family, someone is the victim. I cannot conclude that this happens to women only, maybe that men also can be victims but we receive young women only.

A wife can have problems due to the way she lives with her husband and how she is harassed by her husband. A wife would come here and we would transfer her to the mental health service for help but I do not know how the service deals with such a case. Whether the service has a talk with the husband or not, I do not know. But we received such cases.

Prob: Why can't they tell you that they have family problems in their families to help you know the cause for their trauma?

5*:* May be they tell them those problems, I do not know how they treat them (laughs)

1: In our service, we often see that female persons suffer more from depression than male persons. When we have enough time, we listen to wives who come and tell us about their situation, but by experience it is not easy to bring their husbands.

When you ask her to go and bring him, she tells you that he would kill her if he knew she had come here to talk about it. We would then tell the kind of strategies she should adopt as a mature person who has some rights. You ask her if she prefers to have a headache or to go and take a decision which will save her life.

Prob: *Do those problems often occur?*

1: They mostly occur with wives but there are not many with husbands.

Prob: There is a specific time when they mostly occur. The above discussed trauma problems are general. For the last years there have been special cases due to different causes such as family problems, HIV, or other individuals' problems. Do these problems vary according to someone's living conditions?

1: As time goes by, I think that people start understanding the importance of the treatment provided by the mental health service which is in charge of taking care of them. We often see some people who come and tell us: "talk to that individual, she has a child and needs to be talked to. " You realize that the population starts to understand. But we should not forget the role of the living conditions. It is also another element that cause trauma to people.

Someone may come and tell you that, just as my colleague said about the headache, that sleeplessness is related to the state of the family economy. He may even come and tell you: "I have spent two terms without being able to pay my school fees and I am terribly afraid, I walk on foot, I have no shoes, no school materials and all of this stress me." Considering the prevailing living conditions in general, life is getting more and more expensive and there are many unemployed people. These are some of the causes for their trauma. Some individuals work and study at the same time, and this situation also stresses them. So, I cannot say we receive many people in a given period. But we receive more and more people as time goes by. It means that we will need to hire more employees in the coming years.

Prob: Why do they increase?

1: On the one hand, I think that people face problems and do not find people to whom they address those problems and they keep them to themselves. Nowadays people are getting civilized, medicine is developing and people are coming to hospital. Also, the services in charge of such people are being created. On the other hand, as living conditions are becoming harsher and harsher the number of people with trauma is increasing.

Prob: Number three, can you tell us something about that?

3: Yes! Thanks. As he said, most cases could be identified because of the mutual medical insurance system which allows more people to go to hospital for treatment than before. Frequently, they consult traditional medical practitioner having in mind that it is a traditional illness while it is psychological problem.

But nowadays, as my colleague said, people are getting better understanding of their illnesses. They have access to means of treatment. That is why more cases are identified than the previous periods. Concerning the trauma cases that we mostly face in the emergency service, they come randomly and are made up of people who are HIV positive and take ARV, and other special cases are found in the maternity.

We often have trauma cases during the mourning period including many cases of PTSD, emotional disorders resulting from commemoration period. Then we transfer such ill persons to the mental health service for further treatment.

Prob: Do those cases occur in April or before, or which month do they start, how long do they last?

3: When you observe, trauma is an individual matter but most trauma cases appear during and after the month of April until June. But trauma could be related to a personal history: something could happen to someone at a given time, he/she will later show signs triggered by specific events.

Prob: Among the youth, who are the most traumatized?

3: Mainly young girls, especially, during the national mourning period when they are on holidays after the mourning period or burial ceremonies. We often have many cases after students return to school. We have many cases either when learners are at school or when there is a commemoration in a given district, especially among girls.

Prob: Briefly, more girls have trauma. Why do you think that there are more girls having trauma than boys? Even in ARV service among people with HIV, do more girls have trauma than boys? We mean other trauma problems related to other causes than the genocide.

4: Here, we manly deal with adolescents. Among adults, especially in disharmonious couples, the most affected by trauma are women. Some of those couples may come back requesting to learn how to use the condom so as not to get infected. A woman would say: my husband refuses to use the condom and forces me to have sex without it. So, those women are always in a state of trauma as they are struggling to get through that situation. Others say that they even tell their husbands that they are infected and they are not happy when the condom is not used. And they would ask us what to do. And when you test them again, you find that they are seronegative.

You continue counseling the wife but her husband continues to refuse to come here and know his health status. Although she is seronegative, she tells her husband that she is seropositive in order to live in peace with him. So women are the most trauma affected. On the other hand, there are husbands who have trauma thinking that they have been infected by their wives.

There are many cases where husbands prefer to be away from their wives. They would look for night jobs for themselves and day jobs for their wives so as to avoid being together at the same time at home. More wives have trauma problems than husbands but there are also husbands who have such problems. A woman would also say that she has been infected by her partner. We have a variety of those sorts of cases.

There are also cases of wives who agree to follow the counseling and testing programme but their husbands refuse to follow it. It means that the one who refuses is suspect of being infected. There are husbands who categorically refuse to follow it. Although the wife is on the programme, she has a trauma problem when she says “as my husband has refused to come, what should I do?” You then intervene and ask the husband if he will come. He accepts verbally but he will not come and when you go to their home to urge him to come, he resists. The wife has trauma as she

continues to think that although they were seronegative before they got married, she has been infected by her husband who must be seropositive as he has refused to follow the programme.

Prob: Considering your place of residence and here at your place of work, are trauma-related problems the same or different at your place of residence and at your place of work? Are trauma cases where you live different from those found here where you work? Compare.

5: What we find where we live? We arrive here at 7 early in the morning (they all laughed) and leave late in the evening.

Prob: When you live in a given place as a caregiver, community members submit you their problems? What is the difference between those problems and those observed here?

2: There are more problems here. We spend a little a little time where we live.

3: Those who comes to see you have biological problems such as fever but not psychological or trauma problems.

5: By the way, trauma cases found where we live would mainly be family violence based. But because of the development, women especially housewives provide counseling to one another. They advise their colleagues to call the police when they are beaten by their husbands. This problem has decreased. I have been living here for a long time, but I do not hear any more abuse-related complaints as it used to be the case in 2007 and 2008. Probably, wives could be undergoing other forms of abuse. And Rwandans do not like such cases to be known. Everybody wants to hide their own case. Here at hospital, we receive cases which have been beyond control.

Prob: What about cases among friends? Don't you have some cases of friends involved in violence or trauma related problems? Has violence decreased in your region in the same way as in other regions?

3: Those cases are not frequent.

Prob: Why? Is it because of our leadership or …?

3: It is because of the leadership. This situation is due to GBV sensitization. People avoid being openly implicated in violence as they used to be in the past and says: "if I do it in front of this person, he could spread it out and I could be punished even though my wife could keep it as a secret". The government policy against sexual violence has reduced the problem of such violence.

Prob: Do you think that wives hide their husbands' faults or vice versa?

6: Both wives and husbands hide their faults. It is a question of culture. They think that they may blamed by their children.

3: We often receive cases of wives brought in our emergency service for having been beaten by their husbands and they try to convince us that they for instance fallen down etc... The wife would not reveal the secret even in the presence of leaders, but later when feels more secure she would tell you that she has been beaten by her husband. And when asked her why she did not say it, she replies:" if my husband is jailed, it will be difficult for me to supply food for him". Many people prefer to keep it as a secret.

Prob: What should be the solution so that a woman could reveal that she has been beaten by her husband, who could eventually continue to beat later? How could women be helped to disclose the harm done to them so that they could save their lives?

3: Problems among spouses are difficult to solve.

5: There is a project like Care that seems to be addressing that issue. Care uses people who invite village housewives with no jobs to places like the sector office and sensitize them about their rights, about domestic duties they are to fulfill and they congratulate those housewives on the duties they carry out but which are not under their responsibility and advise them to put an end to that situation. Nowadays, women are aware of their rights. Although they do not express it openly we realize that there has been a change. I think that the solution is continuing sensitizing them. Like those projects, we can sometimes meet our fellow neighbour women and talk about our families. It is not right to talk about everything that happens in the family but things which matter most. We could show them that they can sometime hide their husbands' faults and be later killed by him.

My colleague here has pointed out that wives even fear taking their own responsibilities and those of their husbands. And we show a wife how she could defend herself in case her husband wants to beat her. We advise her not to make a call anytime her husband shoots her an angry look but when necessary she should cry for help so that we could come and save her by having her husband put in jail for at least a week where he would learn to behave more humanly. The solution is continuing sensitizing our neighbors. No pill will be given to them, no syringe will be injected to inject to them but we will just offer them advice.

3: Another challenge is related to culture. Everything could be done but could also be hampered by the Rwandan culture. A wife thinks that she has specific responsibilities within the family. And whatever violence that may happen to her, she opts to accept that situation as the fate of a housewife in a family. Although there are things like sensitization whereby we show her their rights are and what we could do for her in case of violence. But she will always think that she is kidding herself because culturally a housewife is referred to an individual who is supposed to accept all family situations. I think that culture cannot be completely changed but as has been said, sensitization will go on, and although things are not easy to change by now, they may be changed in years to come.

2: I think that in addition to sensitization, women should be financially empowered. Here a woman may be subjected to her husband because she totally relies on him. She accepts to obey all her husband's orders because the latter is the one who provides means to tend to children. I think that if the wife had her own money she could easily divorce from her husband as she would not be depending on him.

Prob: Could she accept to be beaten or abused?

7: Another thing is that churches also advocate an eternal marriage life saying that it is our cross we have to bear. When asked what she could do so as not be killed by her husband, she would then reply that she would do nothing against God's will. She thinks she should not leave her husband as he is her cross she has to bear.

Prob: So, do you mean that the church plays a role in tying together spouses?

7: Yes, the church plays a role in spouses' life because the wife fears to talk about that situation or leave her mate. It means that churches tell them that they must be together forever whether they are ill or healthy. If a husband is aggressive the church makes the wife understand that that aggressiveness is an illness and that she has to live with him forever.

5*: Does it mean that you will accept to be killed?*

1: It is important to highlight this issue of the role of churches our friend has just talked about. We have a case of a wife who left her mate. At that time it was necessary for her to change church which would pray for her. As her parents were not there, she went to live with her brother where she changed church and they prayed for her convincing her that she should forgive her husband and later she was brought back to her home. Once there, they found the door locked. But her husband was not far from there so they called him to bring the key so that his wife could get in. Imagine a husband who has not tried to go and get his spouse back, who has not had an opportunity to discuss the problem with his wife and suddenly he sees her coming back accompanied by churchgoers. The couple immediately started fighting in the open. We immediately received her in the mental health service.

Those cases involving churches are frequent. There is a colleague who talked about culture. Nevertheless, the Ministry of Culture should have helped us with such issues. The youth should be taught the culture without separating boys from girls or men from women, all sitting together. They advise the husband to say it right away when his wife behaves badly, and they tell the wife to do the same when she does not get along well with her husband. The husband is not allowed to do whatever he wants to you. This may be one of the solutions.

Prob: You said that the wife, after having fought with her husband, came to the mental health service, what was her problem?

1: You see, when she came she was in what we can call bipolar disorder. Someone, before being in bipolar state, they start going through a depressive period and then they behave violently, start telling her problems, uttering unrelated words, weeping and suddenly she laughs, dances; we refer this to mania. What is the cause? it is because she felt that she had to go back to her husband accompanied by churchgoers in daylight.

Prob: According to what you said, is there any connection between disorders or mental sicknesses as far as someone has been abused by a partner, be it a husband or wife? Is there any other connection between violence and mental disorders?

1: Of course, there is a connection. I do not know, maybe it is physical or psychological violence as you said before. it is what we said about Rwandan culture. We have things like repression where a person suffers from a long repression until it externalizes itself. Someone said: every suffering held back has its proper time to be externalized in any specific way, but every suffering held back is externalized.

Prob: Are those problems frequent? Are those problems resulting from any form of violence be it physical, sexual or psychological frequent? Those we mentioned by now are mostly psychological and

physical. Do you find people who come to hospital because of sexual violence? Did they come with other supplementary mental problems due to sexual abuse?

5: Maybe I do not know if that one encounter faces such cases, but as for me I once came across a case of a young adult girl who had been abused by more than one person on a street. When she came she was unable to talk to anyone. Everybody who saw her immediately wept. We even failed to know what had happened to her. It is those who picked her who told us that she had been raped by more than one person.

We asked ourselves: "how can't a mature person speak out but keeps weeping only? is this a psychological matter? She said nothing. We led her down on a bed, a specialist in counseling came to talk to her and finally they got all the information from her, but she was quite traumatized. It was difficult even to fill in her medical records because she was unable to say anything.

4: You will even find quite young children who have been raped and who fear every male person they see. We sometimes receive such cases of young children here.

Prob: Does it mean that they will fear males for their entire life?

4: Maybe, We actually keep them for a short time. When they come to our service, we give them prophylaxis drugs. And after that, we cannot know what happens to them as when they finish taking their medicine they do not come back but the problem does not end for all that. You may even follow-up those cases to find out that they are still avoiding any contact with males and rush to her mother for protection when they see one. Why? because of the rape?

Prob: Concerning the problem we talked about, do you find any difference between married persons and single persons?

5: No, I do not know if this happens with married persons, I have never seen mental ill cases due to sexual abuse among married people. But there are many trauma cases due to physical abuse among married people. I just have seen such cases only. There are more married people in that category than single people.

2: Single people are likely to come after having been sexual abused whereas married people are likely to come after having being injured and psychologically traumatized.

Prob: Does this mean that there is no sexual abuse among married people? Is it what you want to say?

1: No, may be it can happen. I agree that such abuse could sometimes occur but it is due to what we have been talking about so far, the culture. When you converse with someone who undergoes violence at home, she would just tell you something related to property, how she is being beaten, how infidelity is committed in front of her, but when you try to ask her about the situation in bed, she says nothing and sometimes she could start crying, she could cry so much as if you are describing her real living situation.

Another one could tell you that she accepted that situation and that that you are interfering in her own business and you have no right to ask her such a question. It is just as if you are wasting

your time. So, it means that if people do not share their relational life, they cannot share their sexual life.

There is something that we did not talk about related to suicide; this form is like an alert sign. Here at the hospital we have many cases of attempted suicide. But this is one of the signs of trauma. When you converse with them, you find that there is a cause for that.

Prob: Are there some causes which make those people commit suicide that you know?

1: Sometimes a child who would tell you that his parents do not treat him like others. I do not know what kind of violence is that one but he insists that his parents tend to favour one of them. Thus, there are many different causes for violence-related issues. There are also cases of children who are not told where they were born. So they find that to be a non-sense.

There are also cases of who start having suicidal ideas. When we ask those suffering from depression disorders if they have never had suicidal ideas, about 70% would tell you that they think about suicide or they tell you that they may end up committing suicide one day. They say" You have talked to me about and I may resort to suicide one day" they say.

5: There is a problem that seems to have decreased but I do not know well if all cases succeeded, I do not see any more wives committing suicide due to their husbands' unfaithfulness. Probably the problem has decreased but in the past there were many of such cases. As my colleague said, there is a difference between an employed and an unemployed wife. A wife who is better off would say that if my spouse is unfaithful it's his business I could also be unfaithful to him. She really does not care. But a woman who has no means, and waits for a cloth, and anything from her husband when she hears that her spouse is having sex with other women, she becomes overwhelmed and thinks of committing suicide. What could push a business woman to commit suicide? She also goes home with the persons that she has spent the day with.

Prob: *I see that our colleague is too quiet; it would better when he could say something.*

6: We said that sexual violence is not found in families but it may exist but is not often displayed. There is a case of a mother who was about to give birth to a premature baby. During medical questioning, she was asked if there were hard chores that she was doing or how she was living with her husband in bed, and she told us that it is her husband's sex behaviours in bed that was the cause for possible abortion. When asked why and she replied: "if possible, you can write a letter to him telling him that if he performs the sexual act once more, the pregnancy will be aborted". Therefore, there is sexual violence in families and among spouses even though it is not displayed. It is kept as a secret.

Prob: A small question before we leave the violence issue. Are there are maybe people who are physically, psychologically and sexually abused and keep quiet? What is the reason behind that? Some people talked of culture, they even say that maybe the wife relies on her husband, are there any other reasons which prevent them from saying that they have been abused?

1: I have a question of curiosity related to what you have asked: we hear people saying that in some developed countries there is the so called marriage contract. Even though there are things

which are against spirituality and even against nature, I do not know whether the Ministry of Health or the Rwandan Constitution say something about it.

There are some disharmonious couples that have problems among them, they often fight with each other every day and security organs have to intervene such as local leaders but when they are friends of yours you would tell her not to leave her mate anymore or not to call you to solve their problems but they would rather handle them themselves. I think, even though it not easy, what do people think about marriage contact?

4: I can give you an example. There are many problems among disharmonious couples that we often meet. There is a woman who told us: "I have reached the stage of telling my husband that I am HIV positive for him to give me a break". They were supplied with condoms but she told us that the first time her husband refused to use the condom, "When I refused, and he bit my cheek and even she took a knife and cuts my thigh". I then had to tell him that I was infected so that I could live in peace with him. Even though she is healthy but she tells him that she is not

5: Developed countries have a way of managing their children's property. They agree on the following terms: "we are getting married, we will spend a given number of years together, and this is the property of the baby that we will give birth to".

Here in Rwanda, we live together as a wife and a husband relying on the property we possess without thinking about our children, what about our child when we separate?

1: This question is sound. We have to know how to make savings for our children and say that that if I divorce Jean Pierre, Tony and Cleo will remain with this and that.

Prob: I don't know when the law is to be passed, so far a husband must engage to one wife. talking about violence, I would like to know if you find abused cases where you live, where do they go to? You told us that you have some cases here at the hospital. Do you think there is another place they go to?

7: They go to the police station or village level.

1: I sometimes hear that there are some women who go to *Duterimbere*.

2: *Haguruka*

4: Problems are solved first at grassroots levels and within families, then by the police and *Haguruka*

Prob: Apart from here, where do other young men having psychological problems go? Where do they go? How do they deal with this?

1: What kind of psychological problems do you mean?

Prob: Whatever trauma problems of any sort. Where do they go? Where do they get help from? I mean young people aged between 20 and 35.

1: Some of them come to the hospital by themselves; others go to see their friends for counseling. Others would come to request for medical care for other persons. This means that maybe they

have to go there first for later orientation. Those people may be friends, an instructor, or a prayer mate.

2: There is even a youth centre for advising youth on reproduction and HIV/AIDS.

Prob: But, do individuals with trauma problems go there?

2: They go there to seek advice.

Prob: Do you think there are others who go there? Someone has mentioned that they used to go to consult witchdoctors, by now there are traditional healers; some treat mental diseases or help mentally ill persons.

4: They exist but no one will accept that they have been there. We may see some of them, as we are Christians, there is a great number of people who go to pray. Someone would say they went to see traditional healers but the Lord had mercy on me. I got healed by witchdoctors but the Lord had mercy on me. And you learn that from there. There others who do visit witchdoctors but you cannot get informed about that.

Prob: Does the church play any role in that? Are there any people who go to church?

All of them: There are.

Prob: Why do they go to church?

4: They tell the problem to their prayer mates and urged them to help them and pray for them or they are visited by their prayer mates them in their homes who reconcile them by using prayers.

Prob: Is there any difference in seeking medicine between ladies and gentlemen who have such problems? It means between ladies and gentlemen with trauma when it comes to seek medicine, is there any difference?

4: Gentlemen delay, males delay seeking medicine. They go seek medicine when things get worse, but ladies, females go there very quickly.

Prob: Why?

5: Generally, men do not seek treatment like women. Even for physical diseases we find more women and children than men at OPD who have come for treatment. Men do not seek treatment as quickly as women.

Prob: When a young person has a mental problem, a mental disease, how does the family handle the problem?

5: The family he/she is from? There are some who have them treated and you find that the family really disturbed, they take good care of him. There are also those who come poor families who cannot do anything to them. There are others who have distant families who not take care of them.

Here in the pediatrics service, we had a case of a mentally ill child whose maternal uncle was at Kivumu, but he spent more than a week without being visited by anyone. We, hospital nurses

who work in the service, were responsible for finding food and treatments for him. He was abandoned because he had a mental disease. It was said that it is his uncle who had brought him. He put him in the car and brought him here at Kabgayi, and later we came to know he had a family at Kivumu. One of our colleagues went there and talked to them face to face and they told him that they would come the following day and we waited for them that day, the following day after and a week later but we did not see any of them coming to see the child.

Prob: Why do you think they have abandoned him?

5: What I was saying is that the situation is not caused by close families. If it had been his father or his mother who had brought him, one of them would surely have taken take of him. This is probably due to poverty. There are also some poor families who are unable to take care of them, others who do not understand the problem because of ignorance thinking that if someone he has a mental disorder, it is his/her own business.

Prob: Are there any other possible causes?

7: Another factor is related to culture, they think that a mentally ill person is worthless to society. This is the way they understand it.

2: Even if he/she comes back, he/she is not given the dignity he/she deserves as he/she is seen as a burden to the family. They even feel ashamed to accompany him saying that it is shameful to live with a mentally ill person.

5: This is poor understanding.

Prob: How do his friends take this?

4: His friend may feel unhappy or

Prob: If maybe one is engaged to someone, how does the fiancé or the fiancée behave? Or even when it is an ordinary friend, how do they behave?

4: There are some who tolerate them but most of them abandon such individuals. They think that it is like bearing one's cross to live with a mentally ill person.

5: But some friends do not abandon them. You could find cases of a fiancé or a fiancée who keeps taking care of his/her partner until he/she gets better and live like spouses. Whenever he falls ill again, the partner will takes care of him/her.

Prob: Young persons, who have trauma problems, how do their families or their friends behave towards them? What happens to the community in which they live?

3: Is it at the hospital?

Prob: When he has been affected, how do his/her friends or family behave towards him/her? When someone has such a problem, how do they deal with it or how does the young man behave?

3: In my opinion, the first thing to do is to take him to hospital. If the family see the symptoms of the illness, they bring him to hospital then, other persons such friends may visit him/her at the hospital to show that they have not abandoned him/her.

Prob: Do all of them get help from their families?

3: Yes. Those who come, they come together with their families or brought their classmates and then followed by their families later but most importantly they are brought where they could get helped

Prob: As for the role of the church in people who have mental diseases, do you find any role played by the church or pastors?

3: Mental or trauma problem?

Prob: Trauma problem and mental disease.

3: Particularly about trauma, I myself do not see any role played by the church but concerning mental diseases we can take an example of the Catholic Church. There are some people that are brought here through Caritas and we realize that they are suffering from mental diseases. They are brought without any family accompanying them, and we treated them in terms of the general trauma.

Prob: What about prayers? Does it mean that there are people who pray for and heal them?

3: Yes. There are, they exist. The day before yesterday, here at Kabgayi Health Center, they called us and told us that they have someone who is unable to collaborate. We went there and we found that he had a psychological problem. He said: I spent some days in evening prayer gatherings where it was revealed that my illness could only be healed when I am in a praying chamber not in hospital. There are cases of ill persons who hope their problem would be solved by prayers. That does not mean such a church or pastors have asked them to come and be prayed for to be healed from their mental disease but because that is the way the ill persons understand it.

Prob: I think that this is one of the causes of their delay as they start with going to the prayers. No other causes?

3: Another thing that we see: there are cases that we used to receive in the emergency service in terms of trauma. Someone would come aware that he/she has a trauma and he/she would be ashamed of being treated. He/she would come late and when you ask him why he/she came late, he/she would tell you he/she asked colleagues to bring him/her to hospital and they refused saying it was fake. So, he/she got discouraged by the ones who should have helped him/her him. And he would become angry and say: "if they say that it is fake, what they will say when we'll arrive at the hospital?" And coming to hospital is a shame in itself. On his part, that is not a disease that should be treated. Sometimes he/she is calm but anytime he/she could be overreacting.

Prob: Do those challenges differ from women and men?

Many cases we often receive are of girls. Sometimes boys also come but more often we receive girls.

Prob: Are there other factors which prevent people from getting treated trauma problems?

4: Poverty. You find that when they lack means of pay for the mutual health insurance, they cannot come to hospital immediately.

Prob: Does it mean that those who do not have the mutual health insurance are not received?

3: They are received but they think that when they do not have medical care fees they will not be treated, so they prefer not to come.

6: Another thing concerning symptoms. An ill person begins to manifest symptoms but he/she does not know where they will lead him to. Someone suffering from fever or from other general health problems could think that he has a headache which will disappear soon or he/she may start keeping too quiet and too calm until things get worse and would then be taken to hospital. They would have failed to take him/her to hospital earlier because of their poor understanding of mental health.

Prob: Regardless of the means of the mentally ill persons what services would you propose they should be established in this hospital? What should you do so that they could all have access to all the services and that those services could be improved?

5: We would request to have an adequate number of staff in our service. We should have for example two staff members so that when one of them leaves, he/she is replaced by another one and the service continue to function. They should each have a working day without not waiting for people to come to his home or they may go through other services because there are symptoms of diseases that could be confusing. We would talk about them in internal medicine while it is the issue dealt with by psychologists. We would like to have a psychologist who would visit any of the services; we would then need at least three psychologists working in hospitalization instead of having two persons working in the mental health service. So, those psychologists would help us to detect disorders timely because we sometimes receive individuals and treat them for disorders they do not have and they go back home without meeting the person who is able to help them more efficiently.

They live far away from health centers that are distant from the hospital. When that individual goes back to the countryside and stays there for a long time, he/she will end up developing mutism as my colleague said. She/he will not be able to speak anymore, what should have been noticed before. If we had an adequate number of staff we would go and see people in villages, advise them on how to behave, how to deal with a given health problem instead of coming after they have developed a serious illness.

Prob: If I understood you well, do you mean mental health workers or clinical psychologists?

5: There should be an adequate number of clinical psychologists are in the hospital.

4: We do not have clinical psychologists here in the hospital; there are those mental health technicians. It could be helpful if we had psychologists in our ARV service. There are very

complicated cases of mentally ill adolescents I told you that we cannot handle. Those mental health technicians cannot help me and it requires that we transfer them to TRAC services to consult psychologists. That requires transport means. We had a child that his grandmother had prevented from coming here. She would say:" You have not been infected. How could an infected child fetch water, or do anything else? Do not let your mother take you to the hospital, where you are given drugs." Afterwards, we went to visit the family and counseled the child who agreed to come here and we returned back here together as I had requested for a car. If the child was put on a public transport bus he might escape and run away. To get him to the hospital was not an easy task either. Luckily, we have in one service a nurse who studied clinical psychology, who went to visit him and brought him in the service by surprise. We had a conversation with him finally he agreed to enter the car but after having been given some nice gifts.

They taught him and finally the child agreed. But we had spent about four years trying to convince the child , he even hid himself and when he arrived here at the hospital, he would go back home. He would run away from other children. We just wish to have a psychologist who could be of great help in general.

Prob: We should insist on what action should be taken so that those who remain at home could come and be consulted.

3: What could be done for those persons is to insert a mental health programme in the programs of health mobilizers. They should add something related to trauma assistance and illness because community health coordinators are the ones who are closer to the population than all the rest. Therefore, they can early detect if anyone has a trauma problem and they can help bring them to the health center.

Or, as the nursing chief said, because there are psychologists they should go to the field to meet people and these coordinators may move as they have transport means, they may even come to the hospital and report if there is any problem in their region. They can even go and see the population at their place of residence and talk with families. Mainly, community health coordinators must be involved in mental health services.

4: Another thing I can add: I do not know if training sessions could be organized for medical staff and IEC sessions to sensitize people about trauma so that in addition to seeking treatment for other diseases they would help sensitizing their neighbours about seeking early medical treatment for their diseases

5: Or when they talked about his/her little sign, he/she would say: "if I passed over there, that may of help.

Prob: Maybe I can add something before we end up our interview, we have talked about violence, what do you think could be done so that there may be improvement in terms of taking care of violence cases. What do you suggest that could be done either in dispensaries or in the community?

3: I think we have firstly to do sensitization as he said. I think sensitization campaigns should be improved by putting public notice boards containing messages along the roads.

So I think that more sensitization should be done so that more people could be aware of their rights and I think those community health people should be operating in the mental health service. They should continue to carry out other health related duties but I think that it is not too demanding if they were involved in mental health programs where they will be dealing with trauma and violence related problems and would accompany health mobilizers with whom they live for most time. So I think that this will help spread out information and they could offer help to trauma persons telling them urgent things to do. Given the large number of cases we receive here, instead of dealing first with a trauma problem they would start going to justice. The first thing they do is to go to the police station to submit indictments. They would be told to come back. They would go to court instead of going to hospital to be treated or for other help. Therefore, I think health coordinators know how to get information about trauma by making them understand what a priority for them and they would help them before it is too late. I think this would help to deal with about trauma-related issues.

I think you have been asking about what could be done for health training or in the hospital. Perhaps, sensitization campaigns should be organized at the community level but what I could suggest is that in health center and in hospital the Ministry of Health should set up a fund for gender or sex related violence. When they would come, we fulfill their basic needs, we treat them and tests are for free, because there is a fund that pays for them mainly for persons who undergo sexual violence.

I wish the Ministry of Health could integrate in the system people who undergo physical violence because we have many of them and they also need expensive drugs. When someone has been beaten by a fellow country person, he is brought to us with cranial trauma and drugs we give to him are costly. We the lend money to him/her as he/she has no financial or we make contributions with the aim of helping him/her. It would be a good idea it there were such a fund that could be used to cover expensed for the treatment of persons who get abused physically or otherwise and have no means to pay. Then we should continue sensitization as caregivers

7: Another thing I want to add is that hospitals should increase the number of those one stop center services in all district health hospitals where someone who has been sexually abused may consult a gynecologist. But you find that a medical record is sent to the emergency unit and where he must be transferred. But if it were a single service to go to, there you would easily find a counselor, a social worker or a doctor. We wish they could establish these services in all health district hospitals if possible

Prob: The last question, when observing people with trauma, what do you think should be done in terms of the follow-up? Is there any follow up, is it functioning, is what is being done enough or what must be done?

5: There is a follow-up is because this service has welcomed the ill person and has counseled him/her and even when it sends him/her back home, it do not cease to help him. There is a follow-up that is carried out by our mental health technicians in health centers. "We could say that today we are going to this health center." They keep on taking care of after patients both at the hospital and those sent to health centers.

Prob: And those who are at home.

5: They are not been in the homes yet because they are not many. We do not think that two people in the district health hospital will be able to take care of those who stay in their homes.

Prob: What do you think must be done so that the person who did not come to the health center can have access to that follow-up?

5: It requires means so that the person who has received him/her may be able to come and visit him/her. It requires means to be able to reach him/her, to have other people replace him in the service and take care of other patients that he has left behind. That is all about it.

Prob: No one else has anything to add

6: It is again the point we have made about inserting that program in health mobilizers' programme. Because if the health mobilizer knew about the case, that the patient has not respected the appointment, the health mobilizer would look for him/her and bring him to the caregiver for treatment.

Prob: In your daily activities, are there anything else that you have found on people who have mental and trauma problems and which we have not talked about?

4: What we are observing nowadays is that the Ministry of Health has implemented the policy of coping with trauma and mental diseases. But among patients we receive here who are suffering from trauma, there are some who do not have the mutual health insurance so that they could be treated.

As far to the service in which I work is concerned, it would be better if there were any assistance so that all people could have a mutual health insurance at least. Yes, there is assistance for poor people but they do not have access to it in time so that they could get treated early. And the number of those that we receive here are quite limited because they have no mutual health insurance.

Prob: No one else to add something
