# Supplementary material for: Gender Inequality Prevents Abused Women from Seeking Care Despite Protection Given in Gender-Based Violence Legislation: A Qualitative Study from Rwanda
Source: PLoS One. 2016 May 6;11(5):e0154540. doi: 10.1371/journal.pone.0154540 (PMC4859471; doi:10.1371/journal.pone.0154540)
Supplement: S4 File — (PDF) [file pone.0154540.s004.pdf]

SUPPORTING FILE 4

INTERVIEW HELD WITH NURSES FROM NYANZA HOSPITAL

Date: 22/10/2012

Duration: 11:05-12:40

Respondents: 7

Q: Let us start. We would like to know psychological and physical problems which are often encountered here in your hospital. I mean problems related to physical or psychological illnesses.

6: The gentleman at the entrance door and this one can tell us what they often encounter.

3: Thank you very much. I think that these problems are unavoidable in our life. We receive such cases on daily basis. Some are related to GBV. In GBV service, we do not only deal with gender based situations, we also have cases of physical violence. These cases are often found within married couples where for example, you may find a husband who has violated the rights of his wife, has beaten her such that she is seriously wounded. These are cases we often have or case of people who have fought, when you examine the reason for their fight, you can find that most of them are from the same family, and the bone of contention is the family wealth. These results into mutual injuries, and such cases are many.

Prob: Do you often observe these cases?

3: We receive these cases on daily basis. No single day without encountering such cases.

Prob: No single day without encountering such cases. You mean cases of people who have fought due to family problems or a husband who has fought his wife or vice-versa?

3: We have such cases here.

Prob: What are other kinds of cases received by other services?

5: In my service of internal medicine, the many cases we often receive nowadays are related to people who attempt suicide by taking chemical products. Within a week, we can for example have 5 cases, 10 cases that have been admitted to hospital due to attempting suicide by using rodenticides, herbicides and insecticides. We have many cases of this kind. We receive like three or four cases of this kind a week. They cannot be less than that.

Prob: Do you try to investigate so that you can identify their causes?

5: It means that we immediately transfer these cases to mental health service for help. I think the person in charge of mental health service is also present. He will tell us about this because he is the one who discusses with these people a lot, but the causes are often rooted in family

problems, a husband and his wife, feelings of depression which lead to despair whereby the victim feels that life is very difficult for him/her, things like that. All these cases are encountered here.

6: About that case, frequencies are many but those who are fortunately recuperated are transferred to mental health service; however, 80% of them having been suffering for a long period of time at home are not recuperated. Therefore, these are cases that come abruptly.

5: Other cases of people admitted to hospital are from drug abuse. We have cases of some people who take drugs, but mainly beers. You find that he/she drinks for example too much beer but after investigating his/her case you may find out that there are other problems which led him/her to much drug-taking. Other cases of people admitted to hospital are related to PTSD. We have many cases of this kind and therefore people always come to us for help. For example, a patient may come to us and get admitted to hospital twice a month and goes back home and later comes back. We have those cases. We transfer those cases to mental health service.

Prob: I would like staff from mental health service to tell us cases they receive.

7: In the mental health service, we receive many cases, especially the ones transferred from internal medicine. We receive cases of people who attempted to commit suicide, cases of PTSD, depression, schizophrenia, maniac-depressive problems, maniac cases, various cases of depression including people with both depression and mania. We receive people having family conflicts, and many of these are the ones who often attempt suicide. There are people who have conflicts over wealth, mainly land conflicts, that arose especially during land registration, resulting into poisoning one another. We receive all these cases in the mental health service. We also conduct psychotherapy, counseling and if need be, we conduct chemotherapy, and we give them a prescription. We receive many cases.

Prob: What are the most frequent cases?

7: The most frequent cases are PTSD and suicide attempt. These cases are the highest. We have many cases.

Prob: Using your knowledge in the mental health, what are the causes of suicide attempt?

7: It is mainly due to negative events which they have been in during their lifetime. Let us take an example of the person who was born and immediately faced various poverty problems in the family and grew up until she got married, had conflicts with him and later, attempted suicide. Let us say, for example, she divorced while she had two children and failed to take care of them because of poverty. All this may lead her to commit suicide. Domestic problems mostly affect women and children.

Prob: Do we observe these cases mostly in men or women?

7: Mostly in women.

5: People who fail to bear problems include mothers or women in general. Coping with those problems may sometimes go beyond their minds but we also receive cases of men these days.

For example, we have recently received 3 cases of children, including one child who has died of sumicombi he had drunk, that's suicide attempt. But in many cases they are of women. They take chemical products called sumicombi.

6: No, these chemical products are used for many things and in coffee plantations. It is a chemical product, I do not know its chemical radical but it is mostly used by farmers. Most people can afford it. For example, the product is found in a veterinary pharmacy. When a farmer is going to clean his/her cattle or to protect his/her coffee plantation from insects, he/she use these products. So, people have easy access to this product. They can easily take them once they have a difficult problem in the family. It is a rare case of young children. A young kid may also crawl and find the product in the storage and drinks it thinking that it is milk. For example, we rarely receive such cases of children but adults of over 25, 30 years old take this chemical product in order to put an end to their lives.

1: Orphans mostly take marijuana due to poverty. This can also take their time and lead them to experiencing psychological problems.

Prob: Do those children experiencing such problems sometimes come to the hospital before committing suicide or taking another decision?

6: Most cases come abruptly and when a person plans to commit suicide he/she does not often make it public. If someone fortunately consults us and tells us he/she has an idea of committing suicide, we recuperate him/her. But most of these cases which come abruptly, recuperation is almost impossible for a person who has made a decision of attempting suicide once he/she has taken that chemical product.

4: I can give you an example of a case of a person who came here yesterday. He told us that he had taken sumicombi, and he further revealed that he did not even want people to take him to the hospital. He was spotted by children, and did not like to be taken to the hospital. However, he was brought by an ambulance, but he had refused to come. He told us that he did not want to come to the hospital since he thinks that he is more intelligent than nurses. Therefore, he fought there was no reason for coming to the hospital. Indeed, he only wanted to die.

7: Still on this issue of suicide attempt, there are good mechanisms we are using mental health service. We are working together with ARV service. Then, those who come for ARV are counseled about daily despair and problems they are facing. There are also questions we ask them whenever they are transferred to our mental health service. They include questions of whether they are at the level of feeling that they need to commit suicide. Once they tell us that suicide is their only last solution they have to resort to, we recuperate them. When it is the case, we make a serious follow-up; we conduct psychotherapy to the extent that they cannot commit suicide. We give them weekly appointments or twice a week if they live near the hospital, we counsel them and try to convince them that committing suicide is not the solution, and that life goes on although they are HIV positive. We are currently recuperating many people experiencing these problems since the mental health service has started working together with ARV service; we really recuperate people planning to commit suicide.

We will not perhaps receive cases of people who have committed suicide we will rather be providing help them to prevent them from having suicidal ideas.

Prob: Do you often have many cases of HIV positive people who attempt suicide?

7: We have many cases. Because they think that if they are HIV positive, they will die and leave their children. They think they would better die instead of seeing their children suffering, starving, etc. But as we counsel them, we notice that it will be over. For those who are HIV negative, we do not know much about them; we often know their cases after they have committed suicide, but HIV positive people nowadays use ARV service, and therefore we get a chance to counsel them before committing suicide.

Prob: Is that a preventive measure to suicide?

7: Yeah.

Q: We would like you to tell us, when you compare today to the past, do you think that all these cases can be observed? I do not mean suicide cases only; all cases you have told me and PTSD including depression. Do you think these cases increase or decrease? How is the situation according to you? Do they decrease or increase with time?

5: Ok, in my opinion, these cases will increase if I look at people admitted to hospital and reports. Patients who use mental health service and other related services for example as well as outpatients, it is clear that statistics have increased.

Prob: Others, do you think it is the same case for other services? There are cases of HIV positive people who can be experiencing other problems and you may treat them by giving them drugs but in addition to that, they may tell you that they have additional problems when you talk to them. Does their number get bigger with time in your service?

2: In the past, in our service, we did not go into details of the problem. I cannot say that cases increased because we were looking at them superficially; we were not going into all details of the problem and therefore, we were not asking them a number of questions. Nowadays, we are into details of any patient's private life who consults us. Therefore, I cannot say that cases have increased, simply because in the past we did not go into details. It is however obvious that when the person is HIV positive coupled with poverty, the situation gets worse. Moreover, it even worsens especially once they are insulted by their families or neighbors.

1: You may also find that even children experience the same problem when they are HIV positive, they are more and more saddened. The parent prefers leaving his/her children and committing suicide instead of seeing them suffering.

Prob: So, what do you think it is the reason? Is it because statistics have increased, is it because they have access to health insurance, is it because the community sensitizes them to come to the hospital? Why do statistics increase with time? What do you think might be the reason?

5: In my point of view, reasons are not many. I cannot talk about one point only. Firstly, we can mention poverty and secondly, we can mention the disease itself. A person can contaminate an illness unconsciously for instance HIV/AIDS or may suffer from schizophrenia

through various ways we cannot go into detail here, but also we cannot ignore domestic problems out there. There are some families which quarrel and most of them fail to cope with the situation. Cases of people experiencing those problems consult in a great number the psychotherapy or clinical psychology service.

3: There is another point I would like to highlight about how they come to the hospital. Most of the time, the patient comes without knowing the real problem he/she has. Let us say for example that he/ she may come and say that he/she has a chronic headache. As he/she is transferred from the health center believing that it is a chronic headache because nurses at the health center are not sure of the illness. Upon arriving at the hospital, we immediately transfer them to the mental health service in order to assess their problem. I think that they immediately sensitize them and this justifies why our statistics have increased. Patients themselves leave the community and come to the hospital without knowing that they have a psychological problem. Upon their arrival at the hospital, we start assessing real problems they have. You notice that it is a problem linked with what we are talking about.

Prob: Basing on what you have just said, do not you think that it is related to the fact that you did not have the mental health service before? Do not you think it is one of reasons why the service can now analyze deeply and identify the problem?

7: That is the point I was going to make. Having the mental health service is very useful because when they come to the hospital, it is an opportunity to analyze and discover the real problem they might be experiencing. But there is another element which helps us to get to these statistics of many patients. In the community, they did not have enough knowledge about mental health. Many people did not know mental health. Now make regular visits to all health centers in Ntunga region. Trainees at the health center do not keep the knowledge for their own; they have to inform the population. They tell the population in general that there is a mental health service that can help them. We inform community health workers, who have information, and this makes the public believe that there is a place where their problems can be solved. They tell them that there are some qualified people who can help them for a given mental illness such as epilepsy. Some people did not know that epilepsy is treated at the hospital but nowadays, I believe that all Rwandans know that the patient can consult the mental health service for assistance. He/she goes for x-ray and is treated even though he/she may not recover completely, but he/she can have a good impression. We notice that sensitization actually helps a lot.

Prob: You, from GBV service, we have talked about mental health problems, but there are some problems of violence we have talked, I would like you to tell us about the issue. How do these cases increase or decrease with time?

3: It stands clearly that they increase. The way our health systems are structured in Rwanda helps us a lot. Because we have community health workers at lower levels, they are aware of the meaning of violence. You find out that people do not start at the village or sector level but they have already seen health community workers. That is the real situation. In addition, you might find that victims were brought to the health center. And after that, health centers can bring them here in the presence of the police. Today, we receive many cases because they are

brought by health community workers or the police or by individuals who have been violated. For example, if the husband violates his wife, she no longer goes to her neighbors like in the past; she immediately files a complaint to the police. The police request us to assess the situation. Therefore, we receive many cases of this kind. Statistics increase with time because people have become aware of the meaning of violence, not because reports say it, but also people who are victims of psychological violence. Therefore, we receive many cases of this kind.

Prob: Are there problems related to consequences of genocide among all the problems we mentioned earlier? What are they? When are they mostly felt? Which problems do you encounter?

5: Yes, there are. Even PTSD cases we talked earlier are mostly related to genocide. Okay, there are also people who come here on regular monthly appointments for advice in the mental health service. But when, the situation worsens during genocide commemoration period in April. We have extreme cases within this period but this does not mean that we do not have regular cases of patients in mental health service. We can give the floor to this one because he is the one to receive them.

7: Thank you very much. On this point related to consequences of genocide especially with regard to mental health, there are very numerous cases. It means that we also have cases beside PTSD cases; some patients come to the hospital after they have developed into schizophrenia. It means that a person might come presenting normal problems from genocide and the situation worsens as he/she gets more and more trauma during the genocide commemoration period. With time, he/she can develop a mental disability; we have these cases in the hospital. There are also many cases of depression such as genocide orphans. Many cases of patients who have problems of depression because of genocide are orphans, without families, they are on their own. We also have those cases of depression from genocide. The number of widows is also very large, they come thinking it is routine counseling, but actually, they are really ill due to consequences of genocide. We have many cases resulting from consequences of genocide.

Prob: In ARV service, what do you have to tell us?

2: For us, all is linked to what you have just been told. There are cases of people who are HIV positive because they were rapped during the genocide. You understand that this is related, during the commemoration, this nightmare comes back, and if there is something which disturbs them a little bit, it resurfaces again. There are also other cases of people who were raped on their way. You understand that there is no difference; they are all the same.

Q: You have just told us cases you receive at the hospital. Now, we would like to know the situation in your community. Are there problems of mental health or violence in your community? For example, cases in which a person comes and tells you how he/she feels.

6: We often encounter cases of epilepsy. You find that a person is not able to identify the problem the child has. But as they live near to us, they consult us since they know that we work at the hospital. They can tell you how the problem has started and you can provide

explanations to them. The main problem that the community faces is drug-taking. Even these cases can be observed in schools which are around the Nyanza hospital. We have many schools but students take drugs, either beer or marijuana. These are cases we mostly have here. In these areas, you may be walking and find students who have deserted their schools, seated in forests for drug-taking. However, the police are helping us to oversee them and to see how those drugs are bought. But these cases are very many. Students, street children and sex workers take drugs a lot. This is a big problem.

7: Still talking about the community, I am going to link GBV and mental health as well as suicide attempt. There are some people for example, in families, where a husband and a wife quarrel. A husband always beats his wife; sometimes it happens to our neighbors, where after some time a wife attempts suicide since she is regularly beaten by her husband. It started as a GBV case where he violated her by beating her every day and after some time she may plan to commit suicide due to this problem. Their children do not feel at ease because they often see their parents fighting. They have a poor mental health growth. They cannot grow well. They grow thinking that fights are normal and as a result, they grow up fighting or develop various mental problems. Those are the cases I mostly observe in the community. They can even change behaviors after a long time due to drug-taking. They always beat people in the area; you find that they are brought to hospital due to drug-taking. That's all in general.

1: In the community, we often have people who mostly have chronic headache. A person may come to you and tells you that he/she has a chronic headache. When we look at those cases, we find that they are psychological and we urge him/her to go to hospital. These are regular cases of patients with chronic headache and insomnia.

6: There are also many cases of syndromes. You find that many students suffer from cephalgia and sneezing. They may come complaining that they suffer from stomachache or backache. Cases of polymorphic syndrome are so many to the extent that you cannot know if initially, patients started with headache, stomachache, backache but if you can guide them well, with the anamnesis, you understand that all those illnesses lead to depression or various problems.

3: There are other cases we have started receiving, cases of houseboys/girls and children they are supposed to look after and violate them. I think that we have received 2 cases of this kind during this week. We have seen cases of house girls who raped kids of 5 years old they were living together. I tried to ask the person who was accompanying the kids if she was aware of the situation and she told me that she did not know the case. I asked her: “How did you know the case?”, “I came to know it after the house girl has resigned. That is when the kid showed strange behaviors and when the mother started touching her child, the latter complained and that she was touching differently from the other house girl.” One mother confined to me before yesterday, and then the kid was brought here. It means that those cases are found in many families, but it is not easy to identify the problem. There is also no follow-up. The fact that we have received two cases means that these cases are available in the families. It means that it is not easy to make a follow-up on these cases because if parents leave children at home when they go to work and return home in the evenings; it is a very difficult problem in the community. Finally, another thing in the community, which I observed in my neighbors, you notice that some prisoners were released and found their wives pregnant or had given

birth to other babies. For that man, it is a problem finding a wife having other kids who are not his or she is pregnant. This case is common for most men. There is another case where the wife committed suicide, another fled, and when the husband returned, he did not know what to do. This case is frequent nowadays.

5: The fact you are doing research is good, but people should be informed of the research findings. Because for me, we still have a lot to do in the health sector. We have a lot of cases while the number of staff is insufficient. You see that in order accomplish well our work and solve the problem; we should have staff in both mental health and psychology. We do not have any psychologist. They have studied psychology, but getting a position which relates to their qualifications, despite they are needed on the health sector is a problem. In order to get enough staff, I think that these research findings will play a good role.

3: There is a point I wanted to stress. We should take measures on health problems which are in the community in the remote areas where we cannot easily reach. There are enormous problems. I do not know how grassroots leaders need to be sensitized or be aware of such problems so that they can help us to make a follow-up on those new problems which arise from the community since they are many. There are more cases which do not reach the hospital in the community.   Even when we conduct field visits, we do not go beyond the health center level. We do not reach the community at the grassroots level; let us say for instance at the village or cell level where we hold meetings, this is not done. This means that there are many problems concerning violence and mental health we are not aware of. There should be new ways to strengthen the community, because today, there is a government policy which establishes a family planning advisor, why should not we have a mental problem advisor as well in the community? The latter can be trained and tasked to provide information as soon as possible because as Rwanda develops faster, the number of mental problems increases as does the number of GBV cases.

Prob: We have not finished yet. There are other small problems. I would like to know the link between GBV and mental health. Is there any link? I do not know if I can call it linkage.

3: You can say that one is the origin of the other because when somebody is violated, the violence does not only affect the body but also the mind. That is why it also involves the mind since even if you violate me physically; I feel pain not only on the body but also my mind will wonder why I was violated. Why was I violated? Is it because I am a woman or a child? Why is that? That is how my brain is affected. When you suffer a lot, a mental problem arises, in case for example, a child who is raped by an adult. There is for example, a woman who tells you that it is due to family problems. A husband comes home from a bar and makes sex with his wife immediately from Monday to Friday. In the long run, the wife gets traumatized, and this can lead her to hate sexual relations. There is a very close link.

Prob: Others, do you have the same ideas?

6: That is how I also find the situation because you also have to think of the mental condition of the rapist. How is his mental condition? You can observe that he is mostly a drunkard. No normal person can plan a sexual violence to a kid of four years old. So, you understand that

we also need to identify reasons which lead rapists to rape children or women. Why do they rape them? Are they mentally fit? They may be having a psychological problem.

4: In addition, you find that some people have conflicts between them. There might be a case of a person who says that his/her child has been raped while it is wrong. And when the child is brought here, the examination proves that he/she was not raped. Sometimes the child itself may be disapproving that he/she was raped, but its family insists that their child was raped, simply because of the existing conflict between families rather than the problem of rape of the child. We have also to try to look at this issue because even the accused is affected by the forged allegations.

1: Telling lies about a person exists but there is a violence attempt the child cannot explain. There might be some cases of violence attempt that the child is unable to explain, but all this constitutes violence. When there has been an attempt, even if the person has touched the child or caressed him/her, which is already rape. Even though forged lies exist but also attempts do exist and finally rape takes place. Identifying these cases is very difficult. That is the reason why we have expressed our worries about the number of staff. We should get enough staff that can help us to discover these cases. Because once the child is brought to the hospital, psychotherapy has to be conducted, counseling needs to be done by a clinical psychologist and the latter tries in many ways to convince the child so that he/she can tell him/her about the rapist, until he/she can discover that the attempt has taken place. So, when there are no employees or even when we are available, we cannot analyze the case deeply due to combining two duties.

Prob: Basing on what has been pointed out, he earlier said that rapists have mental problems; do you think that houseboys/girls also have a mental problem?

1: They do not have a mental problem, it is just a lust. I do not know how it comes, they are weak, and they find it easy for them to rape a child. They profit from the child's weaknesses.

6: We need to be careful about houseboys/girls who are more than 12 years old and commit such shameful deeds. You can ask yourself whether he/she does not take drugs, drinks strong harmful beer known as "*kanyanga*", take marijuana! Because a houseboy with a healthy mind can do the babysitting for the little girl by dressing her, washing her, but a bad houseboy who takes either marijuana or beer cannot. These lead him to committing those wrong deeds. What can you do to identify that a houseboy/girl has such behaviors? Houseboys/girls and cow keepers often have these bad behaviors. For example children who were abandoned, who did not have their parent's pieces of advice, may come looking for a job, and they are given a job while their employers do not know their previous status, whether they were living in drunkard family or in family that takes marijuana. You cannot know all this. When you need a houseboy/girl, you just employ her/him and later on you may often notice that he/she has those bad behaviors; he/she either takes marijuana or drinks beer. And that is the key element which leads them to committing rape.

3: I would like to come back to the link between GBV and mental health. I think that there is a point we need to strengthen. You have seen that people who are mostly raped include children who are under 5 years old. Those raped children who are under five years old in fact,

do not face a mental problem immediately. I am thinking about how their health condition will be after growing up. How will they behave after they know what happened to them? If they are told that they were raped when they were 2 years old, they will present some behaviors. They will be told that they were raped by their uncles; they were here or there, etc. I do not know but in my opinion these are serious cases. Those raped children come to the hospital walking by themselves, and then investigating mental problems is a process. This requires a follow-up, you need to be patient every day in order to understand and evaluate the child's problem. I do not know which measures can be taken. What will be the children's development once they grow up according to you as experts in public health? I do not know measures you are taking for these children once they become aware of their problem. Or they may not suffer from the consequences of the problem once they grow up as the problem will have written off. Indeed, it seems impossible to discuss the issue with them at a young age. But as a teenager, I think that we should do a follow up till the age when he/she can suffer from the consequences of the problem he/she will have transcended the situation. I do not know which measures you will take.

Prob: I think that it is the responsibility of clinical psychologists. Normally, they take measures in accordance with the problem the patient has encountered and his/her age. Unfortunately, I do not have the measures with me but I do not know how mental health service handles cases of those children who have been raped.

7: The system they have been talking about for those raped children has not been established yet. Saying that he/she is now a grown up, he/she was raped at an early age, in fact when we receive them, those with mental illnesses, we investigate their cases from their birth. How did he/she live from birth? In which living conditions? How was he/she born? Was he/she born at home or the hospital? At which school did he/she go? etc. hence, we look at the whole development of the human being. If we find that he/she was raped, there is a way we handle the case, when you have not been told of the case, you cannot know. It means that there should not be any worries because we try to follow-up the person's health from birth to the day when he has come for treatment. When they do not remember, there is nothing you can do but when you are given information, you provide counseling taking into account this information. So, it is good because we start from the beginning, starting from the pregnancy: how was pregnancy like? From pregnancy to 56 years old for example to the day when he had a mental illness? We make a follow-up of all those cases. That is what we do.

1: Normally, rape is a secret. It means that when a child grows old, he/she does not often dare to reveal that he/she has been a victim of rape; it is a shame to him/her. Even his/her family would also keep it as secret although today families have been sensitized to speak out the truth. They used to keep this as a family secret. But it would be better if the child knows it at an early age because it can forbid him/her to get married or to have sexual relations when he/she grows up, and he/she does not have envy due to the pain resulting from previous violent actions. That is why people should sometimes conduct field visits in the community to sensitize the parents not to be shy over rape cases. There should also be staff to make a follow-up of these cases. They should benefit from follow-ups and forget the cases as they grow up. With a follow-up, the victims can grow up and forget these problems, and get to

know that they have had problems like anybody can have them, and then try to forget slowly this with time, which can help them to continue to live their lives when they grow up.

Q: We would like to know, whom do people with a mental problem or victims of GBV go to? Who is that? From your experience in this hospital when people come in your service right from the community? Where do they go or who are there to provide any help?

5: It is a chain. It starts from families where they make recourse to grassroots entities. The latter usually send them to community policing within the community at the cell and sector level. The patient is then taken to the health center which transfers him/her to the hospital. Upon arriving at the hospital, the person in charge of GBV checks her/his case and can transfer her/him to the mental health service or if the case is to be followed up by the police, the latter do it. We have a policeman here who is based at the hospital and he is in charge of dealing with such cases. Patients are followed up on both sides, administrative and health wise. The police follow up the case as regards the violence against the victim, and we follow up the case as regards treating the victim in general.

Prob: Beside consulting grassroots entities, taking the patient to the health center, let alone violence or mental health, does a person with a mental problem only go to the hospital? Are there any other places? Is there any other alternative?

7: As you are saying, in the community, there might be people who do not know this but most of them are aware. There are some people who do not know that a patient with a mental disability goes to the hospital immediately. First, there some who take their patients to sorcerers. And they say that sorcerer X is good at healing this, it is a case of poison or daemons, and others go to churches. So, some go to sorcerers whereas others go to churches, while those who are informed come to the hospital. Not all the people with mental problems come to the hospital immediately.

5: Some patients also come to the hospital at the last minute when prayers or sorcerers have failed to find out the problem. Some come to the hospital when other options have failed. There are cases which came late to the extent that it is very difficult to deal with those cases.

Prob: Some of you have mentioned the hospital, sorcerers, churches, and prayers. Do you think that prayers play a certain role in the patient's health to be in the direction which it should be or they make the health worse? Just tell me the role of prayers.

4: It depends on one's religious beliefs but for us, prayers make worse the health. They make worse the health in our opinion.

5: As you can know, when the patient is praying, he/she thinks that God is saving him/her but prayers do not heal him/her at all. Though there are some witnesses who say that they went to churches and got recovered, but in fact, you cannot say that something which is in blood or brain can totally be removed by prayers. It is an off and on situation.

Prob: Where else do you think they can go? Nowhere?

Those who still have families can sometimes consult their family members. When a person with mental disability is born in the Rwandan family, our culture says that it is a shame. This constitutes a taboo in the family. What the family can do is just to keep this child in the room to the extent that no one can know that the child lives there. This often happens in the families where you can find a person of 30 years old whereas no one is aware of his/her existence. Even when neighbors know that there is such a person in the family, they make it a taboo.

Q: This question goes with the one I was going to ask in the community. How are mental health cases managed? What is the community's mitigating behavior in these cases? One of the cases has been mentioned.

3: Ok, let me start with GBV. How are those cases in the community managed? By the way, let us say that it is sometimes good to bring these cases in the hospital before we manage them there. But nowadays, the problem we still have as I have said is lack of sufficient financial means. We do not carry out follow-ups of those cases after patients have returned home just after some short time in the hospital. I also blame myself on this and I ask myself: "How does this raped young girl live in the community after being subjected to rape?" We do not have financial means to go there and make follow-ups of those cases. This disturbs my heart because these cases are not followed. Patients just come here for counseling services but no follow-ups are made afterwards. We are also disturbed in our hearts wondering how the patients feel after returning home. We do not carry out follow-ups of these cases and neither do health centers manage them. Even at the community level, it is a problem. So, this constitutes a real challenge, we still experience problems in this domain.

Prob: What about mental health? How is the management of these cases in the community? Even the HIV/AIDS service will also tell us. When you have a patient with a mental problem or illness, how do you manage the situation?

7: In the community, it is the same as GBV cases. There are still problems for patients with mental illnesses because there are still some cases of patients we receive, we give them a full prescription but when they get home, they do not have anyone to follow them. There is no one to even check whether patients take medicines at the correct time to the extent that they may fall into a crisis before finishing them. They come back and we may give them another prescription. This is indeed a problem because even I myself who work in the hospital cannot go to the community in order to follow every patient because I have many patients. It will not be possible. That is the reason why in my view, we should take measures which include teaching the community and families in their houses how patients are taken care of. Patients should show medicines to their family members who should make sure that a patient needs to have shower, eat, and so on. Because some patients sometimes come back and tell me: "Doctor, do you know that I live alone? They say for example that they were abandoned by their elder brothers/sisters or their mother because they do not do anything at all." We always teach the community so that it can manage mental illnesses in the community. As regards mental illnesses, sometimes community members do not know well their illnesses. Sometimes community members do not understand that a patient may be in a crisis to the extent that they may beat him/her because he/she has broken some household objects. There

is one case of a patient we have recently transferred to Ndera. He/she was kept in a house and beaten rather than taking him to the hospital. After being beaten, grassroots leaders were able to identify that he/she had a mental illness, and then he/she was brought to the hospital. I discovered that he had a more complicated case and I immediately transferred him to Ndera. This means that we will be treating both wounds resulting from sticks and a mental illness. In my opinion, we should teach especially the community so that it can be able to manage patients of mental illnesses.

3: There is another thing I would like to point out about GBV. I have many examples. For instance, there is an HIV positive man who has raped 2 young girls. One girl is 11 and another one is 9. It was noted that that man had been abusing and raping them for a long time. When the children's family knew about this, they attacked the man in order to kill him. So, this is one of the many ways the community deals with violence cases. People do not think about going to the hospital rather they immediately think of taking revenge on the culprit. For cases of houseboys/girls or animal keepers, the family of the victim also threatens them or takes revenge on them. I have another case. One cow keeper raped the child and the former was savagely beaten to almost death. Before yesterday, we had another case of an HIV positive house girl who was breastfeeding the employer's child. When the family discovered this, she was savagely beaten to revenge on her. Therefore, the community does not understand that the victim should be taken to the hospital rather than keeping this secret. You understand that these are challenges in the community.

2: We also have those cases in our service. To reduce the consequences of the problem, when the patient enters our program we give him/her a person (focal point) who will be following him/her. We also often conduct filed visits to make our follow-up. We visit those people at home. We have many cases of people who even hide that their housemates are HIV positive and that they take ARV. When such people come here, we go into details to the extent that we ask them questions about their family status or through counseling. We try to convince them that life goes on. We also conduct field visit to find some people who may be just drinking beer while they do not know that they are HIV positive. We only provide pieces of advice and follow-ups. We even try to give advice to the person known as *focal point* so that he/she can enter the patient's family because the patient has chosen him/her to tell his/her problems he/she cannot disclose. Unfortunately, there are two people who have not followed our instructions. Dieudonné knows the first person because he brought him/her to us but he/she did not follow our instructions.

Prob: Why did not he follow your instructions?

5: It is a problem. With a person who has a mental problem and is HIV positive, it is not easy to believe that the latter will take medicines correctly without having a family which follows up him/her. The problem is the respect of a prescription that is what he wanted to say. Most often they do not follow a prescription, after some time, he/she abandons to take them. There are many other patients who behave in this way.

Prob: From your experience, in general how is a patient of a mental illness taken care by his/her family, friends and how does he/she take care of him/herself? or a case of a person who has been raped.

5: As my colleague said earlier, in the past, it was a big problem. Patients were isolated by families. As the country develops, there are both health community workers and other people who can take care of such people. The community is taught that patients of mental illnesses are people like others, there are both announcements on the radio and lectures given in different regions which also help. Currently families try to take care of these people; it is not like in the past.

Prob: Others, do you share the same views?

3: I have a different point of view from my colleagues. Frankly speaking, in general, the community or their families do not take care of them. Let me give you an example. Suppose we get out and cross this center. We will meet for instance, two madmen/madwomen. In fact the latter have families, mother or father or both alive. Even when you are walking in the streets of Kigali, you also find patients of mental illnesses. Let me ask you a question, what can you do, what do you help the community to change its mind? Because they tell you that one is just a madman/woman. You can even find him/her on the street naked, walking or begging and so on. "He/she is a mad. Let him/her alone, he/she is known as a madman/woman", some people simply say.

Prob: In brief, you are saying that they have been abandoned by the community.

3: Yes, they have been abandoned. The community often abandons them.

Prob: *There is a bit of a contradiction because it is no longer considered as a shame, they let him/her go wherever he/she wants, without hiding him/her as it used to be in the past?*

3: This is a whole process. They used to take the patient to hospital or use other means. Sometimes he/she is taken to sorcerers, prayers, or he/she may be kept in the house and not allowed to get out. It also happens that the community may stop caring about the patient, and abandon him/her. That is when a patient starts wandering.

Prob: Do you have any other comment to add?

7: I can add is that wandering is one of the signs of this kind of illness. I do agree that there some families which abandon the patients. We have here cases of patients who have families, those who were abandoned and those who quit their families for wandering because you cannot stop them. No matter how you can do, even when they are kept in the house, they can be aggressive and feel that they can wander in the street. There are some who just quit and their families do not know their whereabouts. You may find someone from Burundi here in Nyanza just wandering without knowing what he/she is doing. These are cases that the police apprehend and take them to a given hospital for treatment under government funds. There are many cases of these people in the street.

Prob: You have not said anything about their friends. How do their friends feel? When a friend happens to have a mental illness, does friendship continue or? What happens?

6: There is stigmatization. When a patient tells the truth about the problem, the people around him/her do not value his/her words or when he/she has a crisis which can be over after a very short time, say less than one year or a few days, his/her consideration in the community as a normal person who has recovered takes a long time. There is a feeling of stigmatization.

Q: Is there any other comment you want to add? Let us continue. We have said that some people instead of going first to the hospital choose to go for prayers or to sorcerers, then come to the hospital. What are other reasons why they do not consult doctors whereas they are ill? It means a person has a mental illness or has been raped but does not come for treatment. What are the reasons for this?

5: The first reason is ignorance, then poverty.

6: In addition, there is shame. For example, the mother of 10 years old young girl or less once raped, she chooses to interiorize it, she feel it is a shame, so she hides it. Or if some girls come here and after a pregnancy test we find that they are pregnant, it means that they wanted to keep the case confidential. There are many factors including poverty, ignorance, and intention to hide it.

Prob: Is it the same in a case of rape or depression or any other mental problem? Can parents hide the case even when they know that the child is ill? Others tell me other factors except poverty, ignorance or shame.

3: Another factor is our culture. You find that the latter also poses some problems to the Rwandan community. What I have said, a case of a child who has been raped, after the rape, the victim gets problems, and does not even reveal it, and later she is pregnant. When the family knows about their daughter's pregnant, they ask her what has happened to her. She may say it is mister so and so who raped me when I was from fetching water. "Please keep it secret," the family replies. They ask her to keep it secret and go to the rapist to ask him whether he can marry her. They may even force the rapist to marry their daughter just to hide that problem of pregnancy. Another factor concerns one's religious beliefs. Churches do believe that if the child is from the pastor or a given church, they do not want to let the public know that the child was raped because they believe that it is shame on the family of the victim. Churches also create these problems. They hide cases to the extent that no one shall know that something has happened. That is what I wanted to add.

Prob: With regard to mental illnesses, what are the reasons that prevent patients from going to the hospital? You have mentioned poverty, churches, and ignorance. Can churches forbid patients of mental illness to go to the hospital?

4: Yes, they can. In churches, they say that it a matter of poison.

3: In churches, they even say that these cases result from daemons. You can hear people saying they have a pastor from America who prays for people and they can recover. They take the patient to the church for prayers and stay there for months and months I do not know

how many. As regards our culture, we hear people say that it is a matter of poison. For example we can hear people saying: Nadine was jealous of me, she poisoned my child. Something what they do is to go to a sorcerer for witchcraft, and their little money is burnt for nothing. Another factor is an economic issue. Mental health drugs are very expensive to the extent that they are not affordable to everybody in case the patient has to take them on a regular basis. For example, a parent whose child has epilepsy or depression or other specific problems come to take drugs. It happens that the parent's health insurance has expired or does not have it or was delayed. What happens? The patient just goes to bed and stays without drugs, as he/she will not be able to come for appointments. So, because of those economic problems, some patients do not go to the hospital or stop their drugs.

6: Or some others come from a long distance. It means that we are faced with economic problems and accessibility. We now have health centers which are located at as far away as 15km, 12km or even 30km. Therefore, if mental health service is not decentralized to their health centers, there will always be problems. But still, there is not enough staff in the field, the Ministry has established the program of clinical psychologists but there is no policy governing that makes it smooth so that these people get employed. Out here we have about 3 graduate clinical psychologists but they are jobless whereas they should be working as clinical psychologists. When we see those graduates who did mental health, they can be sent to the countryside whereas others can stay in the periphery. There is also a problem of decentralization and accessibility. They mostly come from the countryside and cannot have easy access although they also have many problems.

Q: Are there specific reasons why both women and men with mental illnesses do not go to the hospital? Are there any specific reasons for one sex?

7: As earlier said, the reasons are various including poverty, ignorance and geographical accessibility, but there are also additional causes. Let's take an example of a wife whose husband does not want the public to know she is mentally ill, the community can say that he is married to a madwoman. So, he feels that if the community becomes aware of his wife's madness, it will be bad and as a result, he chooses to quarantine her. He fears that shame while the victim is kept in the house. This also exists. We hear our patients saying: I have come here secretly because my husband forbade me to go to hospital for a mental illness so that his family members cannot see me. It may happen for example that after a husband knows that his wife is ill, he hates her. When this is the case, the wife goes back to her parents and when she does have them, she can even wander in the street. But when she is lucky, she gets people who can bring her here at the hospital, we follow her case; we are very few as they have said. As we are only two, we cannot reach everybody in the whole community. This is one reason in addition to the shame that most often families fear.

Prob: Husbands forbid their wives, and wives on their turn make their husbands go mad?

7: When there is no other option, she can let you alone in the house and leave.

5: Another general thing is that the community has not yet understood what mental health is. In the past, people knew that Ndera is only for mad people. For them, they knew that anyone who entered Ndera was just mad. Today when you tell somebody to go to Ndera for

treatment, they say that they cannot go there because it is for mad people. They automatically have a feeling that you think that they are mad. This understanding also still obstructs people to consult mental health service for treatment. Unless there is a change of the term and name of the place because wherever they go for treatment, they are referred to as mad people. Most people have this understanding that at that hospital they treat all illnesses related to mental problems, but unfortunately, there are others who still think that it is the place for only treating mad people. Or else, when somebody goes there for treatment, other people may say that he/she went there for madness issue. This prevents some people from consulting this service for treatment. Sometimes I think about that issue because I have received much training in mental health and I lived in Ndera for the period of 3 months. I noticed that many people in Ndera have this understanding that Ndera is for mad people whereas it differs a lot from the reality. If there is a possibility of changing this term or understanding, many people can have access and consult the service for treatment.

Q: Let us conclude by asking you what you think can be done to improve mental health services. What are your suggestions? Everything we are saying is about people who are between 20 and 35 years old i.e. the young generation. What do you think can be done to improve mental health services in general?

1: First of all, the number of mental health staff should be increased because we have very few mental health staff. They cannot receive people and conduct field visits. Another thing which can be done is that mental health should go hand in hand with clinical psychologists. Mental health can treat and give a prescription but clinical psychologists go into details of the patient's problem; they make his/her follow-up since they also conduct a large psychotherapy. That is what I can say.

2: I have no comment to add but we only need a psychologist.

5: Another thing is to change the community's understanding. Firstly, people at the village level should be taught because they have a poor understanding. There should be some lectures either on radios or during field visits. In addition, health community workers should be trained about mental health because once the community understands the problem, it can be completely overcome.

7: Another thing that can be done to improve mental health services in this hospital or elsewhere is that people with mental illness should be given health insurance because there are many patients, when they do not come here after one month; most of them fall into crisis. As a suggestion, all patients of mental illnesses should be given health insurance. I have been working under stress due to people who have been here for 2 months without health insurance. For me, if there is something you can help us is to grant health insurance to these people. They should be given health insurance one by one as it is done for others so that they can be treated on time. When they are grouped in associations, it takes a long time.

5: Health insurance should be individual rather than for an association. This means that people in association get health insurance cards when every member has paid his/her fees to the bank. In case there is one who has not paid yet, members who have paid cannot receive their cards. This means that, for the particular case of some patients with mental illnesses, they can

fall in crisis while waiting for the issuance of health insurance, because they fear to pay hospital services a hundred per cent, they suffer and their statuses worsen, what we are against.

1: In addition, some families have about six family members. Therefore, it is not easy to get the health insurance fees in total; this also takes a long time. This should be a particularity because for us, it is very worrying for patients with mental illnesses.

3: In my view, the mental health problem causes problems for public health. Taking into account our country's history, what we have gone through, the case of mental health gets worse instead of decreasing. In my opinion, mental problems will continue. I would suggest that there should be a focal point in charge of mental health so that at the village there can be at least a person who can explain issues villagers do not understand. Today for example there are community health workers for family planning, why can't we have community health workers in charge of mental health or GBV at the village level? The health system should be built like that because public health has become a public danger for the country. As for the increase of staff, this issue has been highlighted earlier a lot. Moreover, patients with mental problems should have ways of supporting them because drugs are very expensive, hospitals which deals with these illnesses are very few and often located far from the population. Imagine a patient of mental illness from Rubavu or Rusizi to be treated in Butare or the patient's family is told there is a transfer to Ndera, they can choose to keep the patient at home because they already consider him/her as a mad person. This is a problem. There is a problem of insufficient staff and number of hospitals. Hospitals which treat these illnesses are very few. The Government policy of establishing in every District hospital a mental health service is good but the concern is that it was not strengthened a lot. The service should be empowered. Because if a person can get a problem during the night and cannot have any help; these focal points we were talking about, if they are present everywhere, they will provide advice to patients early before going to sorcerers, prayers, this is a problem.

Prob: Any other suggestion?

All: No, there isn't any.

Q: Now, before closing this interview, you can tell me other comments you can add to these suggestions on violence or GBV cases. You have said that the system needs an establishment of a health community worker in charge of mental health. Do you have any other suggestions so that those cases are well managed?

6: I am going to talk about rape. Though the police have started fighting against this to a great extent, the rape disturbs a lot the victim whereas rapists consider this as a normal thing. This means that there are no reasonable measures saying that rapists should be killed as I heard it is done in Uganda, where the Parliament took a decision that a rapist is shot to death. The decision should be applied here because you cannot understand how an adult man rapes a child of less than 5 years. To put an end to rape, the Ugandan system can also be adopted here.

5: I do not support the adoption of that policy 100% because a mental health problem is not something that can be hidden, it is immediately noticeable. I may fall sick and you can know it immediately. That is the reason why both parties should be counseled rather than taking such a decision. Most people think that Dieudonné has raped Y due to the fact that he has an immediate reason. This justifies why we say mental health staff are still few to implement those measures we want to implement. In our families today when a wife quarrels a little bit with her husband, the former intimidates the latter threatens that 'If you continue to disturb me, I will call the police immediately'. In fact, when the police are contacted, they come immediately and handcuff and taken him to prison. You can ask yourself what is going wrong between the wife and her husband. Frankly speaking, there are problems which have not been regulated regarding the above point. Sometimes you can find that there was not a reasonable issue between the couple worth taking the man to prison when you examine their case. When it is the case, one may think of revenge. It is obvious that those cases are connected to mental health. If the husband disagrees with the wife, he may be jailed when the wife calls the police. You understand that there is still a problem. That is the reason why lectures need to be organized for various people.

4: I would like to add that sometimes a 20 year- old girl can lie that a young man has raped her whereas both parties had accepted to make sex. When the problem arises, her family members urge her to say that she has been raped. The accused is taken to the police immediately. Can we also say that the accused of this kind also deserves a death penalty whereas they told lies about him? Such decisions should not be made in case of lies.

3: There is a point I would like to mention again. I would like to talk about education in Rwanda. Starting with what this lady has just said that mental health service is there, but psychotherapy is not well conducted whereas there are psychotherapists. The Government also shows weaknesses as regards making follow-ups. There are other subjects that are taught in education: counseling and guidance. Indeed, guidance is needed. You can do counseling but when you do not do guidance for that person until the end of the problem, you are not of much help. So, guidance and counseling should be taught and be included in the education program so that beneficiaries can know them well. In addition, all the people especially villagers should be taught GBV and mental health. For example, when you read about GBV, you find many definitions of rape or violence but look at its history, you find that you do not know much. A lawyer confirms rape when the doctor has said that a man's sperms have been found in the sex of the child. In this case, the lawyer automatically asks the police to jail the accused. We cannot confirm rape when we have not found sperms in the sex while the child might have been raped. In this case, the accused are released while they have really committed rape. If, for example, a man touched a lady's breasts unwillingly, the police ask for the deposition from the hospital, but no sign could not be provided whereas she was really subjected to gender based violence. Let me give you an example. One day, I was jailed with another man whom I asked why he was jailed. The man told me that he touched the girl's sex with his hands just to see whether the sex had grown. In fact, he had fallen in love with it. You understand that this is a case of rape. The policeman's deposition was attesting that the victim was raped and it is actually rape but how will a doctor discover it? That is how the situation is, a person has been subjected to gender based violence and file a complaint to the

police, the deposition says that X has touched on the sex and the story goes like this. "Dieudonné came and touched with his hands the sex of Martine, this is a case of rape Martine accuses Dieudonné. The former files the complaint to the police who in turn write to the hospital saying that Martine filed a complaint accusing Dieudonné of rape. Therefore, they ask us to confirm if Dieudonné has raped Martine." How will they discover that the victim was raped? What I want to say is that all institutions should know how sexual violence is committed. They should know that we do not only talk about rape when a penis has been introduced in a vagina. In addition, it should be understood that finding sperms in the vagina is not the only sign of rape. All this understanding should end in all institutions; I am talking about judges, police, and grassroots leaders and the community in general especially. Indeed many cases of rape occur in bars. You can see rape being committed but people do not know that they are committing rape. There are many cases of barwomen who face GBV while serving the beer in glasses for clients or you can see a 15 year old young girl entering a bar with an adult. In my view, this should be taught in all areas of the country instead of being only taught to a certain group of people.

Q: Well, before closing, I have a curiosity question that I would like to ask you. You have talked about sexual violence a lot, are there any other kinds of violence here except sexual and physical violence?

3: I have talked about cases of wife/husband beating. They are also frequent.

5: Depriving somebody of his/her rights to education. The fact that one's family cannot pay for his/her education while there are financial means is also a case of violence. When you ask him/her why he/she did not go to school, he/she answers you that his/her family did not allow her/him to go to school.

Q: Is there any other problem you often encounter here that we have not talked about? Is there any other thing we have not discussed you would like to add?

All: None

Thank you.
