# Supplementary material for: Gender Inequality Prevents Abused Women from Seeking Care Despite Protection Given in Gender-Based Violence Legislation: A Qualitative Study from Rwanda
Source: PLoS One. 2016 May 6;11(5):e0154540. doi: 10.1371/journal.pone.0154540 (PMC4859471; doi:10.1371/journal.pone.0154540)
Supplement: S6 File — (PDF) [file pone.0154540.s006.pdf]

SUPPORTING FILE 6

INTERVIEW HELD WITH NURSES FROM CARAES BUTARE

Date: 18/10/2012

Time: 15h11’-17h00’

Respondents: 7

Q: Thanks for having accepted my invitation. We’re going to talk mainly about things you see in your work and how you see patients suffering from mental illnesses. In your day to day lifework, what kind of mental, or physical or psychological problems do you observe among the youth aged between 20 and 35. What problems do you notice?

2: Could you repeat your question, please?

Q: The question is: In your day to day lifework, what kind of trauma-related problems, be they mental, or physical or psychological do you notice among the youth aged between 20 and 35?

2: Among both sexes?

Prob: yes

2: The basic explanation to the first question is related to the fact of being orphaned after losing all close relatives and during the commemoration period when we remember ours who disappeared so abruptly. You could then see a young girl, as I am now mainly talking about young girls who are more often trauma stressed than boys. This trauma state could result from poor living conditions or inappropriate language used by neighbours when talking to her. All of those factors combined will lead to insomnia and she will start wondering why she survived the genocide. Because of that, her mental state and behaviours will be disturbed and she will lose the control of her mind. These are the most prevailing cases

Prob: You have just referred to young girls, why is this situation mainly prevailing among young girls?

2: From my life experience I could say that I am used to receiving mainly girls as the number of boys is rather low, which means that girls are less resistant and more sensitive than boys

Prob: Others.

5: Here at Caraes Butare, we no more receive the category of people suffering from post-traumatic stress disorder but we usually receive those who have the acute stress disorder and who sometimes show symptoms of a serious mental illness. Other cases are referred to ordinary hospitals. We mostly receive here the category of those showing symptoms of trauma and constantly talking about all the events they went through. They will usually show signs of

depression, will refuse to eat and drink as if they would wish to die. Most of those people have usually no place to stay and think that the lack of residence is a very serious problem for them. They think that the fact of having lost their folks is the root of most of their problems. Another challenge we face is that most of those people are drug addicted so that they could have a feeling of peace which constitutes an additional problem to their existing ones.

Prob: Do they indulge in drug abuse before they reach that state or does drug abuse lead them to that state?

5: Most of them indulge in drug abuse after they reach that state of trauma so that they could be able to sleep when they go to bed and forget what happened to them. Another problem is that we do not find families to help us follow them up after we treat them. Once out of the hospital, they have no family support. Without that support, they will constantly be hospitalized for recurrences due to lack of a family to provide them with for proper care.

4: If I could comment on what my colleague said, I would say that there are a lot of cases related to the aftermath of the genocide as my colleague pointed out. There are other problems that are linked to the harsh living conditions someone lives in which could lead him or her to be orphaned because he/she has the feeling of being an orphan.

There are some people who have additional problems such as the problem of identity crisis probably due to the situation in which they were born. They would feel not being members of any family but just orphans. This problem could be the cause for their trauma or or depression. Another element that could be at the root of that situation is the lack of support. Some people could have reached the age of being responsible, but due to poverty and the lack of adequate well-being, they would start showing the symptoms of trauma. They would then come to see him and being in situations where they are incapable of assuming their responsibilities and having abandoned their schooling because of poverty, they might show symptoms that could be associated with illnesses of depression or lack of control of behaviours and mind. Those are some of the hypotheses that could be made from those observations without mentioning their consequences.

Prob: Before making more comments, let us not talk things that might happen but things that we observe, the cases you receive here.

4: Hypotheses that we receive

Prob: I would like to ask him a question about what he has just said: what do you mean by a person having parents but could suffer from identity problems?

4: I just relate the cases we receive here. I may take an example of an illegitimate child who has not been recognized and accepted by the father. The father is rich and the mother poor. The child would want to go and live with the father who does want to receive him/her. S/he cannot identify him or herself with either the father or the mother. Other such cases we have been receiving since I was here are cases related to the situation prevailing in our country. For example a child was born from a mother belonging to the category of those who committed the genocide, the so-called Hutus and the father belonging to the category of those who were targeted by the killers or vice-versa the father is from category of the killers and the mother from the category of the

killed or perhaps the family members on the mother's side have been killed by the family members on the father's side or perhaps within the framework of justice, the family on the mother's side has caused the father to be imprisoned if I could say that. When going to the father's family, the child would be rejected for belonging to the ethnic group that made the father go to prison, and when joining the mother's s/he would also not be accepted for being member of the killers' side and the child has nobody to identify with.

Prob: Are there any cases you have admitted here that have been traumatized due to such causes?

4. Yes we sometimes receive some here

2. I could also add here the case of young girls that get unwanted pregnancies from older people. They are then left in a confused situation with no place where to stay. They are then brought to us for being tested to see if they are mentally ill and finally we find out that the real cause for the trauma is the fact of having been rejected by their fathers because of pregnancy.

Prob: What about those who get psychic problems that have been caused by physical problems?

3: There are people who have been physically wounded during the genocide and are still carrying scars of wounds that have been treated and healed. But during the commemoration period of the 1994 genocide, although the scars have been treated and disappeared physically, mentally they have not, those people carriers will get headaches, trauma stressed and start complaining of pains in the scar whereas there is no pain at all. That situation is just due to the recall of the injury they had in that body area. Another case is of people who underwent sexual violence and during the commemoration period, they remember the event and start complaining that their sex organs are bleeding, when in reality there is no blood at all and they would tell you that they have a pain in the abdomen. They just go stage by stage trough the events that occurred to them. After analysis, you discover that the cause for the trauma is the whole of those events that are behind their constant headaches, insomnia and all sorts of complaints, as lab tests reveal negative. Those are the cases of trauma we usually have here.

Prob: Are there any other types of traumatized people apart from those who have been affected by violence from the genocide that you receive here?

5: There are some who have been traumatized by violence during the ordinary war such as the demobilized soldiers and others who are raped in today's everyday life. When raped, they get infected with HIV and sometimes get pregnant. Rape, HIV infection and pregnancy will be the triggers of the trauma. We therefore have cases of the demobilized persons and the raped

7: There are others who could be traumatized because of disappointment in love. A young girl could be traumatized when she falls in love with a boy who pretends to love her, is deflowered and then abandoned by the boy.

1: Other common traumatized cases are those who have feelings of guilt about their wrongdoings those cases are mostly found among prisoners. They present themselves as being mentally ill when in reality they suffer from the consequences of their possible wrong acts.

Q: Are those cases the same or different among young boys and young girls?

1: Yes

Prob: How could they be differentiated?

3: In general young boys are less affected than young girls because their number is lower. Moreover young boys will be trying to find a solution to the problem even if they may fail to. Young boys will try to find ways of escaping the trauma by indulging in heavy drinking, smoking. You will discover the reality when looking into the boy's history, you will know the cause for the trauma and notice the symptoms much later. Because the number of females is higher, the trauma problem is quicker identified among females than among males. They differ in that as the females' number is higher, you do not need to do a lot to identify their problems as is the case with males. Apart from their higher number, the nature of females is also different from that of males. Females are very emotional towards exceptional situations. They will tend not to accept them and try to find solutions to them. This seems to be one of the reasons why there are more trauma affected females than males.

3: Another element is that when females come to see us we try to help them and give them appointments. And females respect appointments, come back to us and get help. But as for males we could reach, we give them appointments so that we could take care of them, but it becomes hard to get them back unless we call the police to help us bring them back, unlike females who come back easily and respect appointments

Prob: Why are males not willing to come back?

3. This is linked to their nature. Males are difficult to counsel. They feel self-important and tend to minimize problems and do not admit that they are ill.

4: From my working experience, I could say that males try to deny that they have problems and are less open to express them and will try to develop strategies for escaping. Very often we receive them when they have become addicted to drugs. A drug addict will often withdraw into himself and will show that he has no problem. That is why very often when they come here they seem to be in good health because they do not find drugs. They will leave without admitting they are ill. That is why they will not come back. Because of that drug addiction state they will not find where to live and will live on the street. Probably the reason for not coming is that they cannot admit that they have a problem. Unlike the females who, once they have been counseled, they discover that they have a real problem and when they get family support to do the follow up, they come back to see us.

6: Another element that could trigger trauma among the youth is family conflicts. A child from a family where there are constant conflicts will develop a feeling of fear which will affect him later when he/she gets older. Those are also cases that are quite common.

Q: Are the problems the same or different at your place of residence and at your place of work. Probably before telling us where you live could you mention the main types of problems you encounter here, then those from the community where you live?

4. I do not know whether the question is clear enough, could you be more specific?

Prob: I want you to tell me the kind of problems that people have

4: Dou you mean illnesses?

Prob: Yes illness. Then could you tell us whether such problems prevail in your area of residence, the situation in general?

5: Are you probably asking about illnesses or diagnostics of patients we have received and all sorts of diagnostics?

Prob: We want to know what could be seen in general

5: Here we receive a lot of patients, but according to our statistics the largest number is made up of those who are chronically ill, suffering from what we call schizophrenia. The second large group includes those thought to have a neurological problem, those suffering from epilepsy. Epileptics are among the categories of patients we receive here. Others are those suffering from psychotic disorders at their initial stage with headaches before the illness becomes severe. When patients are quickly taken care of and treated, they could recover from the illness. Those constitute the third category of patients we usually receive. There is also another category we receive, that is, those suffering from PTSD and those having anxiety disorders who, if not treated quickly, could develop depressive disorders. This is the category of patients we very often receive and could be the third large category. Patients go through anxiety disorders and PTSD before reaching the stage of depression. Briefly, those are the most common cases we receive.

7: Other patients we often receive are those having mood disorders, i.e, one day they are very happy, another they are very sad (bipolar disorder). Their mood keeps changing.

2: There are others, experts in Karate, who spend their time smoking all types of cigarettes and drinking all kinds of alcoholic beverages unknown to us. This may be the cause for those young patients to be among those cases.

3: Another thing I would like to explain is that any of the individual cases has his/her own personality, his/her own nature and he/she could develop a mental illness according to his/her personality. That means that people could experience the same stressful event and some of them may develop schizophrenia, others may develop mood disorders. You could develop a specific illness according to your personality. People will suffer from different illnesses but resulting from the same history and the same problems. For example when we see someone who likes laughing, we would tease him/her saying that he/she will develop the illness of mood disorders. An individual will develop a mental illness according to the way he/she is, how he/she is, not because of the type of events he/she has gone through.

Prob: Someone mentioned that you receive epileptics; do you receive many of those cases?

2: They are those who consume most of the drugs in this hospital

3: Often people have developed epilepsy as a result of injuries received from the genocide, for having been hit at the head. As it is a neurological illness, the nerves might have been damaged. However there are other causes for that neurological disease, a child may develop that illness as a result of a trauma during childbirth.

7: All of these are associated with other disorders that we could call personality disorders which result from epilepsy. This mental state will lead him/her to be always angry and aggressive and do things that society cannot tolerate, but the main cause for that being epilepsy.

Q: Ok based on what you could see among the youth between 20 and 35, how do they cope with mental illnesses?

4: Do you mean how they support themselves?

Prob: How they manage to get treatment and assistance

4: Based on what we generally see and from my work experience, a small number of the youth get free information on their illness state so that they could request medical assistance or orientation for coming here. There are cases that are noticed by the community or their close families. They are then brought to us by the families who fill in the application form for him and we then receive them according to his/her illness. There are also those who immediately indulge in drug abuse and this is no more a family problem but it becomes a public concern and when things get worse, the police and other relevant authorities get involved in dealing with them. They also cases we receive in large numbers. There are others who are brought to consult us and those who begin with consulting tradi-practitioners because of they are unable to understand that the problem of mental illness is prevailing in Rwanda although it is being adequately addressed but they have another perspective about the issue. There are also among the large number of patients we receive, those who tell you that they first joined prayer meetings that prayed for them to be delivered from evil spirits. They often do this because of their poor understanding of mental health. Those are the cases we usually have and talk to.

Q: Apart from those who join prayer meetings, where do others go?

4: To the traditional medicine

3: In churches, to the tradi-practitioners.

Prob: Others, elsewhere?

4: Others come and see the doctor

3: They are not aware of their illness so that they could themselves decide to come to us but when they are lucky to have family members who are informed about the illness, they would bring them to us.

7: Sometimes due to ignorance, parents or family members do not know where to take them for treatment and may even think that mental diseases are incurable and they keep them locked up in the home so that nobody knows where they are. Because they do not move enough, they are curled up, lose weight probably because they do not eat. In last minute, they are brought to us in a terrible shape. Sometimes families disregard the fact that we treat those illnesses, in that case we then try to sensitize and inform the family about the consequences of such illnesses and how they should take care of such persons and bring them to us for treatment when they notice abnormal signs.

Prob: Ok, is there any specific person in charge of dealing with those persons of that range of age who have such problems? Are there one or more persons to whom they submit their problems?

I wonder if I understand your question well, do you want to know the person they first submit their problem to for assistance?

Prob: Yes

2: As has been mentioned by others, we often encounter cases whose illnesses have been noticed by other people. Apart from those who are conscious of emotional crises they go through with visible agitations followed by periods of calmness, in general all of these elements are often noticed by other people. When the situation is no more normal and reaches the stage of pathology when they cannot eat or sleep, then family members will ask them to make an application. Very few of them will apply themselves but will do it through others.

5: As he says, there are people who are ill but ignore that they are. Even when told that they are ill, they do not accept it. Such cases do not tell it to anybody but other people noticed it and bring them to us. But there are others who are conscious of their illness and talk to their home mates or school mates or work mates depending on where they are. Sometimes there are those who learn from one of our former patients that we deal with such cases of illness and are told to come to Caraes for treatment. There are some who come saying they have been recommended to me by someone and after being treated they quickly feel much better.

3: Often those people come to us when their illness state could be noticed even by the community members. But before reaching that stage, they have had some signs of the disease which could not be seen by the community members and which they themselves did not pay attention to. Those signs may indicate that there is a problem but one may choose to ignore it as people do not fall ill immediately. Another factor is that in Rwanda people are not quite familiar with mental illnesses as they are with malaria. When people get malaria they know they have it as they start having shivers and fever until the disease lays them low. The same thing happens with mental illnesses. There are signs that precedes those illnesses and people who are not familiar with those mental illnesses when they come to see us will mention signs of little importance as they do not know much about mental diseases individually. The illness will be known only when the ill person starts getting seriously ill and the community notices it.

Q. Before moving forward, a question was put to know about your place of residence. What are the problems that you usually encounter elsewhere than here at work? Are there any trauma-related problems you encounter elsewhere which are different from those encountered here?

2: When I go back to my place of residence, I could encounter very complicated cases on my way or elsewhere I go to which do not seem to be much different from the cases I deal with in the hospital where we receive ill people from various districts. We actually observe the same symptoms, the same signs as the ones we notice at the hospital. There is no difference.

Prob: Is it the same situation for others?

4: We live in a society, as my colleague put it. We see some mentally ill persons whose families ignore that those persons are ill: the latter are thought to have been poisoned and they are then

simply maintained in that state. We also see the same kind of ill people in our neighborhood moving about aimlessly with no one to help them get appropriate medical care. Then we pick them and bring them here for treatment as part of our responsibilities to take them to hospital for treatment. That is where one could notice a difference. We also find other people having acute crises during the genocide commemoration period, we then deal with those crises and they return home. I call them crises because they are more serious than those found in our neighbour and could be qualified as exceptional cases.

Q: You said that when those young people get a trauma problem they start dealing with it on their own and will come to see you when the situation worsens but let's talk how their families cope with those situations? How do the families cope with those cases?

7: It could be difficult for some families to accept that state of affairs as having a mentally ill person in a family could be a shame or a stigma in our society which has poor understanding of mental health. Then when a parent brings a child for treatment, he/she decides to take the child back home even if medical caregivers think that the child still needs a longer stay to be proper treated. Once back home, the child has a relapse. As is said, males do not easily recognize that they are ill, that's why they take refuge in drug abuse and engage in uncontrolled acts. Families need to be informed about mental illnesses which are not heredity-linked but could occur in any individual family.

5: Those cases are first thought to be poison-related. A family starts suspecting a neighbour with whom they are in conflict who may have poisoned one of the family members. The first thing they do is to take the ill person to churches or to trade-practitioners as said above. Sometimes it may take very long before they come to see us! They keep him/her there and bring him/her food there hoping that he/she will get better. To give you an example, cases brought here or to Ndera are cases that have been locked up in a small room and are already curled up. We have such a case here. When no improvement is noticed after one or two years, families decide to bring their case here so as to get rid of him/her when the latter has reached the very serious state, when he/she has starts destroying the house, beating everyone in the home, when it has become impossible to keep him/her at home. You could hear people saying that they would wish to find a place where to send him/her to even if he/she could die there. They would be providing for his/her needs there. When they bring them here, they beg us to take our guy and keep him/her in hospital as they are not willing to take him/her back home. That is the way the community deals with the situation. Another experience I found in families that often come to see us is the case of young adolescents who at the initial stage have a kind of delinquency or behaviours deviation and become impossible to manage. This behavior is one of the elements that will refrain them from developing into an adult, and instead of going to school they become delinquent whereas that could be the beginning of the illness. There might be some causes for those behaviours but most families would describe it as a kind of ordinary delinquency for boys and prostitution for girls. Families find it difficult to consider it as the beginning of the disease.

Prob: Is it that they consider as delinquency or as prostitution?

4: No they do not consider that as a disease. They keep on saying that they do not know but when the case turns out to be more serious, they then start considering it as a disease, probably

due to poisoning or as a case of delinquency when the young person prefers playing truant to going to school and wandering around whereas it is actually the beginning of the disease.

Prob: What about his/her friends? What do friends do?

4: Sometimes what they call friends are bad friends they usually share beer with instead of being good friends who could help them turn into productive people. Young people we know as drug addicts when they get money they would take it to buy drugs from their gang members who call them "our man" as most of these young people or their friends tell us when we talk to them.

Prob: What happens when he/she reaches the stage of PTSD?

4: When he/she starts withdrawing into him/herself, he/she will have two categories of friends. Those who will stigmatize him/her because of the difference existing between him/her and them. There are those who would come to help him/her but when in PTSD, he/she would see them as horrible people who may be coming to kill him/her The crisis is made up of hallucinations or flashbacks and even the medical caregiver is seen as someone planning to kill him/her. This is the way the categories of friends could be identified. At the end of the crisis, the friends are again seen as normal friends. During the crisis, he goes back to the events that triggered the crisis such as war. Those crises often occur during the genocide commemoration period when people recall the events they went through. His/her friends are seen as people at war who want to kill him when he/she is in crisis. Out of the crisis, he/she will continue to live with friends in harmony. However some of the community members will stigmatize him/her.

Prob: Could others add something as members of the committee of medical caregivers or as participants?

5: What I have noticed is that it will depend on the group you belong to. For example, as far as university students are concerned, they bring their friends/classmates who are ill to us and would appeal to authorities' assistance when needed. Even when the ill person has no mutual health insurance (mutuelle), each of the friends will contribute financially so that he/she could be consulted and treated. Another ill person could be belonging to the group whose members meet to share beer and find him/her useless with no more contribution he/she is bringing in to the group. He/she would be rejected by the group and live by him/herself and lonely. There are therefore two categories of friends: those who take care of the ill persons and get them treated and those who neglect them and leave them on their own.

2: There may also be some bad consequences as most of those who are rejected and fail to identify from any group end up killing themselves.

Q: We have talked a bit about people who approach churches when they have a trauma problem, do churches provide some kind of assistance. Do churches or prayers help decrease or increase the trauma??

7: As a believer, churches should help us as caregivers by providing what we could call existential dimension whereby the patient has faith and believe in the existence of God. But when he/she start saying that it is God who came and healed him/her, we consider that as part of mystic-religious delirium because he/she thinks that everything is done by God whereas we, caregivers,

look at various approaches: the role of medicine, the biological dimensions, the impact of drugs we give ad we reinforce the use of psychotherapy by engaging in conversation with them. Therefore having faith is necessary and to that end, religious celebrations are organized here but when things are exaggerated and patients think that they will be only healed by God and spend all their time praying, there is then some kind of deviation. That religious dimension is considered within the framework of the care provision process.

Prob: So within that framework, do churches contribute to decreasing or increasing the trauma?

1: Churches could have two influences. For example to those who tend to give up hope or to be remorseful, churches could help them find peace. But for those whose health deterioration requires intensive use of drugs or medical care for them to regain forces, churches could contribute to the worsening of the situation and the chronicity of the illness.

Prob: Are there many or few people whose healing is hampered by church interference?

2: I do not know but so far I have not heard of any case that has been healed in church. On the other hand, churches help us reinforce the care we provide but do not heal illnesses. The church will teach the patient how to respect the schedule set such as taking medicine as required, going to church so that he could live in peace at home.

Prob: So do they help them or what kind of care do they provide for them?

2: I cannot say that they do not help or they provide treatment. There are two dimensions. A person seriously ill should go and see a medical caregiver but if he/she is on treatment he/she could go to pray but take medicine on time as required, respect medical appointments and follow schedules. Prayers are quite easy to say for a believer.

6: What I could add is they are often taken to church by their relatives who are believers and think that when a person is affected by a trauma it is because he/she has been attacked by evil spirits. That is why they take him/her to church so that God could save him/her from those spirits. That does not mean that they have been taken there because they believe that God will heal them. It is a kind of refuge where they hope to find God who could help them get rid of those spirits but it does not mean that the ill person will be healed. I would like to say that when he/she goes there, the acute signs of the illness could decrease as they could do so without going there. They would then say they feel better thanks to the prayers; they would go back home and over time, the signs will develop and show back again. We cannot say prayers are a cure for mental illnesses. That is what I wanted to add

4: I would like to add something that I have noticed from two perspectives. Most of the people we receive have had recourse to prayers, witch doctor tradi-practitioners if I could combine those two elements and I will explain why I combine them. Most of those we receive first appeal to those elements: prayers and witch doctors. Both going to pray and to see witch doctors have something in common which I could call a psychotherapy element. In prayers there is what could be called spiritual psychotherapy and even experts recognize that fact and medical caregivers use psychotherapy which is faith-based. If I say that such faith-based psychotherapy is not helpful, some people could get upset. There could be cases that will be concerned with only such psychotherapy which will address an ill person’s sadness. If they pray for him/her depending on

his/her faith in God, his mental health problems will be solved and his/her crises will come to an end. A mental illness is different from malaria; it is a chronic disease. It should be treated by the combination of psychotherapy sessions and drug-based therapy. It is in that framework that churches could be helpful as they operate only that side. If the ill person needs the combination of the two, he will have a short-term remission, he will have a short rest. If he/she is not in the hands of informed religious people who are able to advise him/her that besides prayers, they should also go and see medical caregivers, the community as a whole will gain nothing and the delay will worsen the situation and the illness will become chronic. Churches will have failed to help us. I believe that churches could help us or not from that perspective: a kind of spiritual psychotherapy combined with another approach. It means that acts from those who use prayers and of witch doctors are all faith-based. When a witch doctor gives you a piece of wood or stone picked from water to carry on you, he/she wants it to be your protector and the accompanying words he/she utters are a kind of psychotherapy. If you really believe that you are protected by the small stone you will also need another approach which is biology-based. This approach is going to help solve another part of human problems which cannot be dealt with by prayers and witch doctors. It is in that sense that they could help us or complicates the situation.

5: As many experts say, for a human being to lead a harmonious life, he/she should be in good physical, mental, social and spiritual condition. For all those needs to be fulfilled there are religious people who should help us with their spirituality and give the human being hope for life. You have probably been in church and heard the priest or pastor saying: if you face problems of disease or poverty, (these are actually the main cause for mental diseases), know that God is there to put an end to them and you then become healthier and thank God. The ill person of course has the feeling that soon he/she will recover from his/her disease. There are some pastors and priests who say in their sermons: if you take medicine from medical caregivers and listen to the word of God you will recover from your illness soon and will go to haven. The ill person will regain hope that he will recover and will believe in that. Sometimes believing could create and that word of god could contribute to the change of mindset and the way of thinking

Prob: Is this the way you see things or you understand them?

5: That’s how I see them

Prob: Ok is this as you see them or as you think they should be? is spirituality providing support to medical treatment or vice versa or do they both contribute to the treatment of an ill personal?

2: they are complementary.

4 They should be complimentary but people should live their lives. Although they are complementary but it is normal that when a believer gets ill he/she should go to hospital and also pray by saying: God I know that it is you who heals all diseases even the incurable ones, get me touch the lights and healed but I am going to hospital.

Q: I would like to ask you a question which is quite similar to the one we are talking about, in your work, what are the main big challenges that hinder people from getting or seeking for any kind of medical care as we saw that they could go wherever they want to get it, what are the main big obstacles that could prevent the youth from getting the medical care they need according to cases?

3: The first obstacle is poor understanding from all categories of the population, the educated and the uneducated; in general the Rwandan population does not understand all that are related to mental illnesses. But after 1994, mental ill cases increased but mental health still remained not well known. In brief that is what I could add.

2: Another element is poverty. I mention poverty because a mentally ill person is a kind of parasite in the family because he/she is there to consume without bringing in any contribution of any kind. That means that every month the ill person should present him/herself to attend helpful counseling sessions if he/she is not given drugs. Poverty is an element that could have an impact on the success of the follow up process.

4: Another element that hinders the youth from healing is the problem of social integration: the fact of being rejected by families. An ill person comes to hospital and is treated and gets better and returns back to hell. Such patients who are in a very bad shape are called resigned patients by technicians. They are resigned because after we have treated them, we send them back to their families who have rejected them because of poverty. As we have not educated their families who have poor understanding of mental health, they are going to increase the number of chronic ill mental persons we see wandering around on the road. The lack of family support is another major challenge we have. Another important obstacle is poor understanding of mental health from the higher to the lower state organs. For example, if statistics show that the mortality rate is high in Rwanda, that rate is due to suicide and homicide cases, while the Ministry of Health which is in charge is incapable of entering those data. Let's take a clearer typical example to illustrate the poor understanding which could make things worse. Recently the Ministry of Health has written to the Ministry of Education requesting to cancel the department of clinical psychology from university. Here there is a problem. The high mortality rate is due to suicide and homicide which result from poor mental health. If there are no trained professionals to provide orientation and counseling to the affected people and their families it is going to be difficult to address the issue of mental health. Now there are only mental health staffs who deal with cases in hospitals but not from the grassroots level. All of those challenges contribute to increasing the cases of ill mental people instead of decreasing them.

Prob: Do you do anything with families?

4: Yes we talk with them

Prob: You treat people but what else do you do when they leave the hospital so that they could recover from illness?

4: We talk with them when they are still in hospital. We invite those we can reach to come and talk with us but again because of poor understanding, there are families who do not respond to our invitation. There is one case found by the police and his family came to see him whereas it had rejected him forever. As nobody but ill persons could stay here, he would then have to go back to a family who does not want to understand him or to come and get support from us so as to be taken care of as an ill person

Prob: Where do you send him/her to? Who tells you where to send him/her to?

4: Sometimes we accompany him/her to his/her home and we talk with the family. You understand that if a family does not visit a relative in hospital for two months, even if we accompany him/her, the one-day session of talk with the family may not be enough. The latter could agree to keep and help him/her when we are there and two months later, the same person could be brought back to us again rejected by his/her family

1: There are such many cases in families who do not come to visit their folks at the hospital. They are then brought here by the police who have found them moving aimlessly on the road with no family to help them. They are unable to say why they have been brought here what is also a big problem. And when they get better, they find it hard to get back to normal life without having any support. There are even cases that resume abusing drug which will trigger their mental illness crisis

Prob: Ok you have mentioned the political decision regarding the police intervention. I am wondering whether such a decision is not causing any problem to you, the centre and the whole community. There are ill mental people who stay in your community and fail to come and get treated here, what hinders them from coming and get treated here, a health facility they know quite well.

1: This is linked to what we have already said. Most of them get ill but do not come to get treated because they have no financial means. There are still people who have neither the mutual health insurance card even if they now that it is the most dominant health insurance scheme nor money to immediately pay cash for treatment. Because of the problem of awkward beliefs and poor understanding of their ill mental health, they will consider their illness as being a surnatural phenomenon they cannot do anything about. They could either go and consult trade-practitioners or resort to prayers meetings or they will just wait and see. Those are the situations that mostly prevail.

Prob: Do those hindrances to getting treated affect young boys and young girls in the same way?

7: In my opinion, females are more vulnerable to and more affected by those illnesses than males. They have difficulty recuperating and supporting the illness. If they get HIV or pregnant, the illness gets more acute. But when they take medicine as required and follow advice given to them and their families, the illness status could remain more or less stable.

Prob: How do those hindrances differ from males to females?

5: They are quite different as my colleague put it. When a male decides to seek medical treatment of his illness, he will do it. He will work and earn enough money to continue to get his illness treated and could also easily get married while being mental ill. But this is not the case for females. When a mental ill female gets married, her illness will get worse. She will often seek advice to know if it would be suitable to reveal her illness to her future husband, if the latter would not reject her if she did so! Probably, that female is in a period of remission and seems to be getting better whereas she could have a relapse at any time. There are some female cases after getting married have a relapse, then get rejected and are incapable of working to earn money so that she could come get medicine. They then start wandering about on the road. When a male decides to seek treatment he managed to get financial means by doing petty jobs such carrying loads from fields. Males manage to earn money but females do not get any money at all.

Prob: How do hindrances differ from married to single people?

2: In my opinion, hindrances differ according to sex as others said. We have had female cases that have had mental crises when preparing for marriage. Those crises are due to the fact that they panic when they fail to find what they need fo their marriage as they are orphans and have nobody to help them. They fall mentally ill and are brought here to hospital. We have noticed that when they leave hospital, the marriage fails to occur. We have also had cases of drug addict males who, before going back home after treatment, have sought advice from their caregivers in the following terms "Now I am going to get married and have a partner who is going to help me with my illness and she will even be coming to collect medicine for me but she will not know that I have had acute crises before". Those are male cases that have understood and even if they have no families, they eventually decide to get married and have a partner to help them. We have noticed that their spouses came to collect medicine for them for example people like Manuel who is sitting there. Such males are better taken care of than females. In my opinion, females are very vulnerable when it comes to take care of them especially when they have no family to follow them up.

4: I was thinking of the type of problems encountered by both married and single persons. I have no statistics but mental illness-related problems of the married and the single persons are mainly due to poor understanding of the community. When a single person shows signs of a mental illness, he/she will have difficulty finding a partner. We receive a lot of female cases who have never got married because they have been rejected because of their mental illness. Or they get married, thus fulfilling a basic human need, for a short time and get divorced because of their mental disease. As to the married they continue leading their married life with their illness. The unlucky ones split up due to the mental illness but there others who continue to live together. But very often when someone gets ill before getting married, he/she has little chance to get married and in a married couple when one of the partners gets ill, they split up. Most cases we see are those who have been rejected after getting ill. And it becomes difficult to summon any of them: when it is the husband who gets ill, the wife has already abandoned him and joined her family and if it is the wife who gets ill she has already been rejected by her husband. I I think that those are most of the cases we see but I have no statistics comparing the number of the married and the single people. Single people have problems and there are among the married those who are lucky and continue to be taken care of by their spouses and most of the cases I receive here are divorced or about to divorce due to tremendous problems they have

Q: OK, if it was necessary to propose an extra health service to improve the existing ones you provide, which one would you add or establish?

7: As far as we are concerned, we could say that we are still limited as we are a branch of Ndera Hospital. Our hospital services are not computerized and it becomes a real problem to find data about a patient who loses his/her identity documents. It may take us a long time to locate where their documents are or we may establish new ones when we cannot find them. It means that if new documents are established, some previous information on the patient may be lost. The situation is being improved but we are still limited but there are still problems in the neurology service for those have neurology health problems.

Prob: Ok could you think of something that could be added so as to improve the good functioning of your services without considering the problem of limited means?

7: Awareness rising is needed so that the population could understand what mental illness is as most of them ignore what it is because of lack of information. We should tell them the causes for mental diseases, their symptoms, how to get treated. We have got to reinforce care education in the community

Prob: In the community or?

7: Even in villages and institutions like health centres, schools and in places where many people meet, but our time is limited as we have other duties to carry out.

5: Service that could be added here in hospital!

Prob: Not only in hospital but in general what could you wish to add to improve the quality of the health mental services you currently provide?

5 : Our services are adequate but what is missing is sensitization so that all people could understand what mental health is, identify a person suffering from a mental illness as they have been well informed about that. Due to lack of qualified mental health caregivers, a significant number of mental ill people die. The study carried out in 2000 shows that out of 100 who committed suicide, 60 were mental ill. They had been suffering from depression and some of them may not have been to hospital for consultation. Why didn't they go there? Because neither themselves nor their families nor local authorities knew that those people were ill. If all community members knew to recognize those mental diseases and their symptoms, the situation would be better. Another example is that in some locations we visited within the framework of mutual health insurance campaign it is required for the whole family to subscribe to a mutual health insurance subscription. No family member could subscribe individually. When on the field visit, we clearly explain and inform people about the policy, they agree to deliver the mutual health insurance to individual family members who are ill. They even provide a mutual health insurance for the category of poor people so that they could go to hospital for treatment. Once they get better understanding, they would even encourage and remind families to take their ill persons to hospital, why? Because we have been there. What is needed most is visiting those communities and providing them with IEC psycho-education sessions to help them acquire better understanding of mental health. That is what is really needed.

Prob: You do not do that, do you?

5: We do

Do you?

3: But not enough

5: This is the way we do it here: In the morning IEC sessions are given to patients or those sent by ill persons who have been unable to come when they are still outside waiting for consultation. In IEC sessions we first talk to them about mental illnesses we have mentioned above such as schizophrenia and its symptoms, epilepsy and its symptoms. Finally, we tell them that when they

see persons from their families and neighborhood showing symptoms of one of those illnesses to inform them that they are suffering from such or such an illness and advise them to come and get treated at Caraes. They will have been very helpful to them. And if necessary they could also guide them or even bring them to Caraes. We also tell them what ill persons are required to bring with them when they come here, such as the Mutual or Rama Health Insurance and money to pay for drugs like in other health facilities. This is what we usually do in the morning.

4: As for me I would like to talk about mental health not at the centre level but at the national policy level. This is an example of what I would wish from authorities. Let's take the case of malaria. All Rwandans know that in the last two years, malaria caused a very high mortality rate, but for the moment, malaria has been eradicated thanks to effective strategies that have been put into place. Two days ago I had a chance to attend a workshop and was surprised to learn that the mortality rate was high in Rwanda. I would therefore like the Internal Ministry to make data available because the police have more data probably than the Ministry of Health. During the training the doctor working for the police told us that they register more than fifty cases of both suicide and homicide per day. We have been conducting studies on that issue but he told us that he will have obtained the figures in a matter of six months

2: In a short period

4: What then should be done? I think that the health policy and the Ministry of Health should put much more effort into dealing with mental health illnesses as they did with malaria so as to improve mental health as my colleague put it. During talks and community work, the population is taught how to eradicate malaria and is distributed mosquito nets, free of charge. A lot of money has been spent on that exercise, as my colleague said; there are still problems as regards mentally ill persons. As they have been rejected by their families, it becomes difficult to get a mutual insurance for them as they have no address and they have not been recorded during the ubudehe programme census and there is no record of them in state organs. It is obvious that no much effort was put into dealing with mental health as was done in other areas of health whereas it is associated with a very a high mortality rate. It is as mental health contributes most to the high mortality rate. The health policy should set up appropriate strategies for addressing mental health issues from the central level down to the grassroots level. This is what I think should be done so as to improve the welfare of the population in terms of mental health.

Prob: What should be done to set up such strategies?

5: What I think should be done is carrying out studies showing the statistics of deaths caused by mental illnesses similar to the reports made on the deaths caused by malaria in CHUK or CHUB. Those figures will raise the community's awareness about the gravity of the situation and the whole country will feel concerned about the problem and find appropriate action to take to address it.

4: I could add that in matters of health, the Ministry of Health should work in collaboration with the Ministry in charge of Security. For example, the mortality rate does not include people who die out of hospital which has no record of them. The cases of people who die from suicide will be recorded by the police, not by the hospital. They will not be part of the statistics of the hospital because no medical expertise has been carried out to know the cause for their death whereas

most of the figures should be recorded by the Ministry of Health, This is the reason why the Ministry of Health should work hand in hand with the Ministry in charge of Security which should provide it with data on all the people who die out of hospital. How come a person kills a relative for land conflicts? That means that the person has a mental problem even if he/she has not been to the centre for consultation. That is the reason why there is a need for decentralization whereby adequate explanations could be provided for better understanding of areas like this one. There are some errors that are made in areas such as the departments of clinical psychology or of social work or sociology where students in those departments have poor understanding of how to identify a mentally ill person. What is a mentally ill person? It is the person who has already been recognized as an ill person by the clinical psychology. When is he/she called a mentally ill person? When he/she recognizes that he/she is ill. However there are other signs such the alarm sign as you have said. If there are permanent conflicts in a given family, they need an experienced person who could approach and talk with them. A family where there are permanent conflicts may cause one of its members to be depressed. A healthy wife for example who is always threatened by her husband could end up developing sudden signs of depression. There is therefore a need for decentralization whereby psycho-social centres could be established at the sector level for example to deal with such families where there are such conflicts. That health service used to be called psycho-social consultation. Its denomination is quite adequate but in some places, the service is also involved in providing medical care instead of dealing with preventing the causes of diseases such as family conflicts and depression crises. There will be conflicts, wives will be beaten and threatened. There is therefore a need for preventive measures such as sending workers on the field. As my colleague put it, even Caraes has no capacity to go to the field to follow up the person who has come here and admitted that he/she is ill. Those are the strategies that should be set up: going to the field and teaching how to identify mental problems and when to come here.

Prob: Has anyone any comment to make?

1: As has been highlighted, prevention against mental illnesses without neglecting the cases of vulnerable people who have handicaps. If rehabilitation centres are created to deal with some diseases such as multi resistant tuberculosis centres, would it not be possible to establish psycho-social rehabilitation centres for mentally ill persons. Such centres could help improve the life quality of those mentally ill persons.

Q: Ok I have another question to ask. Is there anything we forgot to mention in relation to mental illness?

5: You have talked about illnesses and ill persons but you did not talk about drugs that are used in the treatment of those illnesses. Are drugs available? Do persons in charge of procurement try to get modern drugs? Drugs used in the treatment of mental illness which are no more used in Europe are sent to Africa. Although those drugs could be efficient in the treatment of mental illness, they could also have side effects on patients. However, in Europe there are drugs given to human beings and which have no side effects at all. Much effort should be made so that people get appropriate and modern medication which has no side effects on ill people.

4: I would like to add another point. So far there is one neurological hospital in Rwanda. However, as there are a small number of mental health agents in districts, they do not hospitalize

patients but transfer them in our unique hospital of Ndera. One could wonder why the ministry or the government does not think of allocating a hazard duty allowance to the hospital workers. Although workers may take protective measures, they may also be infected by a patient having a virus. Furthermore very often we receive mentally ill persons who are brought to us handcuffed by the armed police who find them dangerous and hand them over to us. When they reach here we, unarmed, remove their handcuffs for treatment. Authorities should consider the risks run by caregivers who are beaten or have their limbs broken. When a caregiver is in trouble he/she gets assistance from volunteers. There is no law providing for insuring those workers against those hazards and have nothing to claim in such situations. However I know some cases who had been infected as a result of handling ARV and whose medical treatment was taken care of by the Ministry of Health. But mental health has been left in the hands of charity organisations whereas the Ministry should be aware that we are also government workers and treat us as such.

Prob: Thank you. Sister, do you have anything to add which has not been mentioned?

2: What I would like to highlight is our concern for those people aged between 32 and 35 who are criminals, therefore have a behavior they cannot control. They kill neighbours and their relatives and instead of being imprisoned by state organs they are brought to the centres for treatment because they are suffering from mental illnesses. As far as we are concerned, we have no protection against those people's violence brought here by an armed police who may frighten them. Sometimes they jump over the fence and vanish into nature. Then what could happen if such an individual fell down and died when there is no law protecting hospital caregivers who take care of such people or patients? Is this of no consequence on caregivers who should have a law to protect them in such situations or protect the person who dies?. There should therefore be good working relationships between the hospital and the government and have measures to protect caregivers especially against drug addicts.

7: In that context, we could add the fact that authorities should get more involved in mental healthcare provision. As most often mentally ill persons often wander around and have no fixed residence, they live in places where they were not born. They may be living in Huye when they were born in Nyaruguru or Nyamagabe. Very often people think that it is the police that should have them treated but the district authorities should also have a role to play in this process. As we have no identification of the ill person and do not know his/her name, after treating him/her, we approach the social service so that they could try to find his/her families as the police have failed to do so. In that case, we think that the district should get involved in that exercise.

Q: I do not know if there is any comment. Otherwise I would like to add the fact that I have heard people saying there may be a link between mental health-related issues and sexual violence. Is there any relationship between mental health and sexual violence?

3: Of course! in the community?

Prob: I mean in the community

3: A person with a deep mental trauma who consumes drugs, abuses children, beats anyone he/she sees, will cause insecurity and the police will end up handcuffing him/her and bringing him/her here. He/she is among the cases that are not picked by the police but we hear talked

about on radio: a boy killed his mother, a husband killed her wife. Those are violence-related acts. No normal healthy human being would kill a parent.

Prob: Are there here cases of trauma or mental illness caused by sexual or any kind of violence?

4: We have a lot of depression cases due to wives' harassment by husbands or mistreatment by families. There are a lot of cases of trauma due to all kinds of psychological abuse. We have many cases of mood disorders of depressive persons often due to violence they undergo in the community

Prob: Are there many of those cases?

4: Yes, there are

Prob: No sexual violence?

4: There are indeed

Prob: Are there traumatized cases due to gender-based violence?

2: There are indeed

Prob: Do you have now among those we talked about who have been traumatized because of those problems?

7: There are those with unwanted pregnancy because they have been raped; there are other children who have been raped and infected by a disease.

Q: Is there any difference between married and single people or do those problems affect the married and the single in a similar or different way?

5: There could be a difference. Sexual violence which is physical and psychological is usually found among married people. The most frequent cases are those who are beaten, harassed, insulted and forced to have sex after being beaten by their partners. Then little by little they develop mental illnesses which will later turn into acute trauma disorders. For people who are still young and living with parents they usually face property-related problems. Instead of being provided with adequate living means, the child is deprived of his/her property by family members who have sold off part or all his/her property. The child will be short of financial means and start hating life and drop out school. When it is time for him/her to build his/her house, he/she has no plot or land to build the house on. This is a kind of property-based violence which is different from sex-based violence.

Prob: Some people face such problems but do not get to you. Are there those who may have trauma problems or other health problems but do not get treatment??

5: There are many

Prob: Are there more than those who come here?

5: Many of them are those children you would find at commercial centres where meat is roasted, places where drugs are consumed, gambling games are played. In general, those children are ill due to property-based violence.

Prob: Why do you think they do not come to get treatment?

5: That is due to the reasons we have talked about: they do not know that they are ill, they do not know the signs of their illness, or due to poverty and lack of orientation they do not know where to submit their problem.

6: What I could add here is that we receive those who have reached the stage of illness. They do not really care about their illness and let it develop until it gets worse and then come here.

Prob: How do you know that such or such mentally ill person has been sexually assaulted? Do you use a special technique to know that a mentally ill person has been sexually assaulted or do you get informed by the police?

3: We follow up his/her case when he/she gets better, talk with him/her and then she tells us his/her story

Prob: Do you finally manage to know his/her problem?

2: Or he/she brings sex-based evidence to us, then we test him/her and carry out investigations and analyze gestures made during the act and then we treat him/her. There are also cases of sexual transmitted diseases.

Prob: I think that is all unless.

7: I would like to say that from experience of hard work with mental health, we encounter some cases which could seem strange to people who are not quite familiar with mental illnesses and they do not work in that area. They see mentally ill persons as people who are used to beating and hating others without any reason. They ignore the fact that they do that because they are mental ill. If you had stayed here longer, you would have noticed that most of them want to get out of here and return home because they feel being in prison and not free. They call the hospital a prison. But as they are recovering from their crises, they discover the good side of their being in hospital. There is another very good thing. There are times we receive a case of a person who refuses to talk, who is completely mute and does not make any gesture at all and you start wondering if he/she will ever recover. He/she is then put in hospital and given appropriate treatment and one or two weeks later, he/she gets better and starts talking and will talk too much. He/she will then start telling his/her story as communication is re-established. That is the symptom of mental disease which will help provide appropriate treatment.

Prob: The last question of curiosity, do mentally ill persons live alone where they are found or is there anybody else who stays with them?

7: Haven’t you seen a person wearing a green apron?

All: Yes, we have

7: He/she is a member of the health worker team. There are two of them. That one is in charge of hygiene and there is another one in charge of security who would intervene in case a patient engages in fighting. There is another worker who assists the nurse and comes down to make a report to the nurse. There is always someone to oversee patients. When somatic problems occur, we refer the patients to our part-time doctor who consult them and if necessary decide to transfer some cases to psychiatric hospitals.

Q: Another question, do you work with health mobilisers so as to sensitize community members about coming and getting treated here?

7: We are working in collaboration with many people and we have their telephone numbers but there is no integrated health system. The mental health policy advocates that when a patient is treated and gets better he/she should return in his/her community so as to make room for other patients When a mentally ill person receives no visit from a family member, we do all we can to find from health coordinators, persons in charge of social welfare, executive secretary of cells or sectors information about the ill person. There are however some mental ill cases who lack mental security because we have failed to find the addresses of their families and have been here for a long time. For such cases we try to find a way of integrating them in such places as orphanages or other centres that could provide care to such persons. There are some unidentified cases of mentally ill persons brought by the police. When you ask them who their father is, they answer something like it is a sweet potato. They are unable to give you adequate information about their place of residence; on how they used to live…You cannot take them back to the police who have brought them to you unless each of you tries to avoid responsibility. We try to request the social service operating here to find a solution to this problem by seeking information on how and who brought the ill person and where the latter was brought from. The service carries out investigations and by chance they manage to find the family of some cases but there other cases whose families are difficult to find out. But the process continues.

4: There is no specific way of coordinating and collaborating with other health structures so that training in the area of mental health could be provided to them. For the time being, we make some arrangements to get together with them but there is no specific formal way of meeting with health advisors and other structures that work with them.

We thank you for the time you devote to this interview about mental illnesses We are really grateful and promise that you will be sent the findings from this study so as to improve mental health services in Rwanda

Thank you.
